# Natural and synthetic flavonoid modulation of TRPC5 channels

**DOI:** 10.1111/bph.13387

**Published:** 2016-01-13

**Authors:** Jacqueline Naylor, Aisling Minard, Hannah J Gaunt, Mohamed S Amer, Lesley A Wilson, Marco Migliore, Sin Y Cheung, Hussein N Rubaiy, Nicola M Blythe, Katie E Musialowski, Melanie J Ludlow, William D Evans, Ben L Green, Hongjun Yang, Yun You, Jing Li, Colin W G Fishwick, Katsuhiko Muraki, David J Beech, Robin S Bon

**Affiliations:** ^1^ School of MedicineUniversity of LeedsLeedsLS2 9JTUK; ^2^ School of ChemistryUniversity of LeedsLeedsLS2 9JTUK; ^3^ Clinical Physiology Department, Faculty of MedicineMenoufiya UniversityShibin Al KawmEgypt; ^4^ Institute of Chinese Materia MedicaChina Academy of Chinese Medical SciencesBeijingChina; ^5^ School of PharmacyAichi‐Gakuin UniversityNagoya464–8650Japan

## Abstract

**Background and Purpose:**

The TRPC5 proteins assemble to create calcium‐permeable, non‐selective, cationic channels. We sought novel modulators of these channels through studies of natural products.

**Experimental Approach:**

Intracellular calcium measurements and patch clamp recordings were made from cell lines. Compounds were generated by synthetic chemistry.

**Key Results:**

Through a screen of natural products used in traditional Chinese medicines, the flavonol galangin was identified as an inhibitor of lanthanide‐evoked calcium entry in TRPC5 overexpressing HEK 293 cells (IC_50_ 0.45 μM). Galangin also inhibited lanthanide‐evoked TRPC5‐mediated current in whole‐cell and outside‐out patch recordings. In differentiated 3T3‐L1 cells, it inhibited constitutive and lanthanide‐evoked calcium entry through endogenous TRPC5‐containing channels. The related natural flavonols, kaempferol and quercetin were less potent inhibitors of TRPC5. Myricetin and luteolin lacked effect, and apigenin was a stimulator. Based on structure–activity relationship studies with natural and synthetic flavonols, we designed 3,5,7‐trihydroxy‐2‐(2‐bromophenyl)‐4*H*‐chromen‐4‐one (AM12), which inhibited lanthanide‐evoked TRPC5 activity with an IC_50_ of 0.28 μM. AM12 also inhibited TRPC5 activity evoked by the agonist (−)‐Englerin A and was effective in excised outside‐out membrane patches, suggesting a relatively direct effect. It inhibited TRPC4 channels similarly, but its inhibitory effect on TRPC1–TRPC5 heteromeric channels was weaker.

**Conclusions and Implications:**

The data suggest that galangin (a natural product from the ginger family) is a TRPC5 inhibitor and that other natural and synthetic flavonoids contain antagonist or agonist capabilities at TRPC5 and closely related channels depending on the substitution patterns of both the chromone core and the phenyl ring.

**Abbreviations**
LPC: lysophosphatidylcholine
S1P: sphingosine 1‐phosphate

## Tables of Links

**Table:** 

**TARGETS**
**Ion channels**
TRPC1
TRPC3
TRPC4
TRPC5
TRPM2
TRPV4

**LIGANDS**
Apigenin
Englerin A
Galangin
LPC, lysophosphatidylcholine
Luteolin
Quercetin
S1P, sphingosine 1‐phosphate

These Tables list key protein targets and ligands in this article which are hyperlinked to corresponding entries in http://www.guidetopharmacology.org, the common portal for data from the IUPHAR/BPS Guide to PHARMACOLOGY (Pawson *et al*., 2014) and are permanently archived in the Concise Guide to PHARMACOLOGY 2013/14 (Alexander *et al*., 2013).

## Introduction

In mammals, twenty‐eight genes encode transient receptor potential (TRP) proteins (Damann *et al*., 2008). These proteins assemble to form homotetrameric or heterotetrameric cationic channels, which are most commonly localized to the plasma membrane. Although there are similarities between different TRPs, they are diverse in sequence and the assembled channels are differentially activated or inhibited by physico‐chemical signals, including hot and cold temperatures and a plethora of chemicals, some of which are natural products such as capsaicin and menthol (Vriens *et al*., 2008). Several of the TRP channels have attracted attention as potential targets for drug discovery efforts, for example, TRPV1 or TRPA1 in the analgesia field.

The C subfamily of TRPs has seven members, TRPC1–7, although one of them, TRPC2, is not expressed in humans (Abramowitz and Birnbaumer, 2009; Beech, 2013; Birnbaumer, 2009; Bon and Beech, 2013). The TRPC subfamily is the one that is most closely related to the first‐identified TRP of photo‐transduction in *Drosophila melanogaster*: hence C, for canonical. The properties of the TRPCs have been reviewed (Abramowitz and Birnbaumer, 2009; Beech, 2013; Birnbaumer, 2009; Bon and Beech, 2013). Here, we focused on transient receptor potential canonical 5 (TRPC5), which, like most other TRPCs, assembles as homotetramers to form non‐selective Ca^2+^‐permeable cationic TRPC5 channels, but it also heteromerizes with other TRPCs such as transient receptor potential canonical 1 (TRPC1) (Beech, 2007; Zholos, 2014). TRPC5 is often noted for its expression in the CNS and is sometimes indicated as being exclusively neuronal. In this context, innate fear and pro‐epileptic roles have been suggested for TRPC5 as well as roles in growth cone formation and other neuronal functions (Greka *et al*., 2003; Phelan *et al*., 2013; Riccio *et al*., 2009). TRPC5 is, nevertheless, also expressed in peripheral tissues where non‐neuronal roles have been suggested, such as in podocyte barrier function, cancer cell multidrug resistance and adiponectin secretion from adipocytes (Ma *et al*., 2014; Schaldecker *et al*., 2013; Sukumar *et al*., 2012). TRPC5 channels can exhibit constitutive activity but are also modestly or strongly stimulated by various externally applied factors that are not specific to TRPC5 but include lanthanide ions (Gd^3+^ and La^3+^), sphingosine‐1‐phosphate (S1P) and lysophosphatidylcholine (LPC) (Flemming *et al*., 2006; Jung *et al*., 2003; Xu *et al*., 2006; Zeng *et al*., 2004). Lanthanides appear to act as direct activators or facilitators of channel opening, whereas S1P acts indirectly via G protein signalling (Jung *et al*., 2003; Xu *et al*., 2006). There is a view that TRPC5 forms a receptor‐activated channel and that this is its physiological purpose, but there is also the view that it is activated by stress factors without the need for receptor activation (Birnbaumer, 2009; Jiang *et al*., 2011). Both may be true and, indeed, TRPC5 channels, like several other TRP channels, show what is variously described as versatility, promiscuity or multiplicity of activation (Birnbaumer, 2009; Jiang *et al*., 2011; Vriens *et al*., 2008; Zeng *et al*., 2004).

As with other members of the TRPC subfamily, the pharmacology of low MW ligands for TRPC5 channels is relatively underdeveloped, often lacking potency and specificity and often not acting directly; various modulators of this type have been reviewed (Bon and Beech, 2013; Jiang *et al*., 2011). There is emerging evidence for synthetic low MW modulators, which include sigma‐1 receptor ligands, the antihistamine clemizole hydrochloride, riluzole, the 2‐aminoquinoline ML204 and the 2‐aminobenzimidazole derivative M084 (Amer *et al*., 2013; Miller *et al*., 2011; Richter *et al*., 2014a; Richter *et al*., 2014b; Zhu *et al*., 2015). There is also evidence for antagonist capability in dietary substances that include ω‐3 fatty acids and antioxidant chemicals, such as vitamin C, gallic acid and the polyphenol resveratrol (Naylor *et al*., 2011; Sukumar *et al*., 2012). Conversely, a remarkably potent and selective activator exists in (−)‐Englerin A, which derives from the plant *Phyllanthus engleri* (Akbulut *et al*., 2015). Such sensitivity to natural products aligns with the findings for other TRP subfamilies and a general concept for TRP channels as integrators of animal biology with physical factors of the external environment (Vriens *et al*., 2008). Here, we sought new information on TRPC5 channel modulators by testing a small set of natural products from traditional Chinese medicines.

## Methods

### Plasmids for TRPC1/TRPC5 and HEK‐TRPC3 experiments

Human TRPC3 was cloned into the pcDNA™4/TO expression vector (ThermoFisher Scientific, Waltham, MA, USA) between KpnI and XbaI restriction sites using hTRPC3/pcDNA3 (from M Zhu, Ohio State University) as a PCR template (forward primer: 5′ CAGTGGTACCGCCACCATGGAGGGAAGCCCATC 3′, and reverse primer: 5' ACACTCTAGATCATTCACATCTCAGCATGCTG 3′). To facilitate cloning of SYFP2–TRPC1 and mTurquoise2–TRPC5, a four amino acid linker (ASAS) flanked by AgeI and SacII restriction sites was introduced into pcDNA™4/TO between EcoRI and XhoI restriction sites using Gibson Assembly® (New England Biolabs, Ipswich, MA, USA) (forward oligonucleotide: 5′ CCACTAGTCCAGTGTGGTGGAATTCACCGGTGCCAGCGCATCCCGC 3′, and reverse oligonucleotide: 5′ GTTTAAACGGGCCCTCTAGACTCGAGCCGCGGGATGCGCTGGCACC 3′). Fluorophores were inserted upstream of the linker between KpnI and AgeI restriction sites using pSYFP2‐C1 (Addgene plasmid # 22878; Kremers *et al*., 2006) and pmTurquoise2‐C1 (Addgene plasmid # 60560; Goedhart *et al*., 2010), gifts from Dorus Gadella, as PCR templates (mTurquoise2/SYFP2 forward primer: 5′ TAATGGTACCGCCACCATGGTGAGC 3′, and mTurquoise2/SYFP2 reverse primer: 5′ TATTACCGGTCTTGTACAGCTCGTCCATGC 3′). Human TRPC1 (forward primer: 5′ ACTTCCGCGGCATGATGGCGGCCCTG 3′, and reverse primer: 5′ TTGCTCT AGAAAATGGTTAATTTCTTGGATAAAAC 3′) or human TRPC5 (forward primer: 5′ GATCCCGCGGAATGGCCCAACTGTACTACAAAAAG 3′, and reverse primer: 5′ GGGTCAAGGAAGGCACG 3′) was inserted downstream of the linker between SacII and XbaI restriction sites using hTRPC1/pIRES (Xu *et al*., 2006) and hTRPC5/pcDNA™4/TO (Zeng *et al*., 2004) as PCR templates. All constructs contained an N‐terminal Kozak sequence.

### Cell culture

HEK 293 cells stably expressing tetracycline‐regulated human TRPC5 (Zeng *et al*., 2004) or TRPC4 (Akbulut *et al*., 2015) have been described. An equivalent cell line expressing TRPC3 was established. Cells were maintained in DMEM‐F12 + GlutaMAX‐1 (ThermoFisher Scientific) supplemented with 10% fetal calf serum (FCS) and penicillin/streptomycin at 37 °C in a 5% CO_2_ incubator. For selection, 400 μg·mL^−1^ zeocin and 5 μg·mL^−1^ blasticidin S were included in the cell medium. To induce expression, cells were incubated with 1 μg·mL^−1^ tetracycline (Sigma‐Aldrich, Gillingham, UK) for 24 h prior to experiments (Tet+). Non‐induced cells without addition of tetracycline (Tet−) were used as control. HEK 293 cells stably expressing tetracycline‐regulated human TRPM2 were prepared similarly and have also been described previously (McHugh *et al*., 2003). TRPV4 was studied in CHO K1 cells stably expressing human TRPV4 and maintained in Ham's F12 (ThermoFisher Scientific) in the presence of 1 mg·mL^−1^ G418 (Sigma‐Aldrich).

The 3T3‐L1 cell line was obtained from the American Type Culture Collection and cultured in DMEM‐F12 containing 10% fetal calf serum (FCS), 100 U·mL^−1^ penicillin and 100 μg·mL^−1^ streptomycin. To induce differentiation, cells were grown to confluence, and 2 days post‐confluence, the medium was changed to a medium containing 5 μg·mL^−1^ insulin, 0.25 μM dexamethasone and 0.5 mM IBMX with 10% FCS and antibiotics. After 48 h, medium was changed to a maintenance medium containing 5 μg·mL^−1^ insulin, 10% FCS and antibiotics. Cells were fed with fresh maintenance medium every 2 days until the day of experiments. For all experiments, cells were differentiated for 12–16 days.

### Intracellular Ca^2+^ measurement

Induced (Tet+) and non‐induced (Tet−) cells were plated in poly‐d‐lysine‐coated black 96‐well plates (Corning, Corning, NY, USA) at a confluence of 90% 24 h before experimentation. Cells were incubated for 1 h in 4 μM fluo‐4‐AM, 2 μM fura‐2‐AM or 4 μM XRhod‐1‐AM in standard bath solution (SBS) at 37 °C in the presence of 0.01% pluronic acid (ThermoFisher Scientific) and, for fluo‐4‐AM and XRhod‐1‐AM, 2.5 mM probenecid. SBS contained (mM) the following: 130 NaCl, 5 KCl, 8 d‐glucose, 10 HEPES, 1.2 MgCl_2_ and 1.5 CaCl_2_; the pH was titrated to 7.4 with NaOH, and the osmolarity was ~290 mOsm. Cells were washed three times with SBS before measurements were made at room temperature (21 ± 2 °C) on a 96‐well fluorescence plate reader (FlexStation II^384^, Molecular Devices, Sunnyvale, CA, USA). Fura‐2 was excited at 340 and 380 nm, and emitted light was collected at 510 nm. Fluo‐4 was excited at 485 nm, and emitted light was collected at 525 nm. XRhod‐1 was excited at 580 nm, and emitted light was collected at 610 nm. Readings were made every 10 s. Fura‐2 measurements are shown as the fluorescence (F) ratio or change (Δ) in this ratio. Fluo‐4 and XRhod‐1 measurements are shown as absolute fluorescence in arbitrary units or changes in this fluorescence (ΔF). For experiments requiring no extracellular Ca^2+^, BaCl_2_ replaced the CaCl_2_ in SBS. When required, pretreatments with flavonoids were for 30 min at room temperature prior to recordings and maintained throughout. Control cells were treated with DMSO (vehicle) as appropriate.

### Electrophysiology

Current recordings were made under voltage clamp using the whole‐cell or outside‐out configuration of the patch clamp technique at room temperature. Cells were seeded on glass coverslips at 20–30% density. Signals were amplified and sampled using an Axopatch 200B amplifier and pCLAMP 8 or 10 software (Molecular Devices). Data were filtered at 2 kHz and digitally sampled at 4 kHz. The voltage protocol comprised voltage ramps applied from −100 to +100 mV or every 10 s from a holding potential of 0 mV. The extracellular solution was SBS, and the patch pipette solution contained (mM) the following: 135 CsCl, 2 MgCl_2_, 1 EGTA, 10 HEPES, 5 Na_2_ATP and 0.1 Na_2_GTP, titrated to pH 7.2 with NaOH. All solutions were filtered using a 0.2 μm filter (Sartorius, Göttingen, Germany). TRPC4 or TRPC5 cells were induced by tetracycline 24 h before experiments. For TRPC1–TRPC5 whole‐cell recordings, HEK 293‐MSR cells were transiently transfected with SYFP2–TRPC1 and mTurquoise2–TRPC5 according to the manufacturer's instructions, with the following modifications: 4.5 μg of each construct and 5.5 μL Lipofectamine®2000 (ThermoFisher Scientific) were used. Cells were transfected at 90–95% confluence in a 35 mm culture dish, and transfection was performed for 4 h. Patch clamp experiments were performed on the cells 24–48 h post‐transfection.

### Chemical syntheses

A library of 41 mono‐substituted flavonols **4** was prepared using a two‐step synthetic procedure (Scheme S1). The galangin analogue, 3,5,7‐trihydroxy‐2‐(2‐bromophenyl)‐4H‐chromen‐4‐one (AM12) was synthesized in four steps (Scheme S2). All synthesized chemicals were >97% pure according to ^1^H NMR and ^13^C NMR analyses. Synthetic and analytical details are reported in the Supporting Information.

### Chemicals and stock solutions

Commercially available chemicals were purchased from Sigma‐Aldrich, unless stated otherwise. Stocks of chemicals were reconstituted in an appropriate vehicle: fluo‐4‐AM, fura‐2‐AM and X‐Rhod‐AM (ThermoFisher Scientific) were dissolved at 1 mM in DMSO; pluronic acid F‐127 (ThermoFisher Scientific) was stored at 10% *w/v* in DMSO at room temperature; probenecid was freshly prepared at 0.5 M in 1 M NaOH and diluted to 1:200 to give a working concentration of 2.5 mM; galangin, apigenin, kaempferol, quercetin, myricetin and luteolin were used as 10 mM stock solutions in ethanol. All other flavonols were synthesized and purified (for details, see the Supporting Information) and used as 10 mM stock solutions in DMSO. Stock solutions were diluted to 1:1000 into the recording solution, giving a final working concentration of 0.01% solvent. Gd^3+^ and La^3+^ were used as aqueous solutions of GdCl_3_ and LaCl_3_ respectively. 1‐Oleoyl‐2‐acetyl‐*sn*‐glycerol, thapsigargin and 4α‐phorbol 12,13‐didecanoate were all dissolved in DMSO and stored as 50, 5 and 10 mM stocks respectively. l‐α‐LPC from egg yolk and S1P were dissolved in methanol and stored as stock concentrations of 5 and 10 mM respectively. ATP and H_2_O_2_ were stored as aqueous stock solutions. Englerin A was prepared as a 10 mM stock solution in DMSO, stored in aliquots at −80 °C and diluted to working concentrations in experimental buffer (e.g. SBS) containing 0.1% DMSO and 0.01% pluronic acid. Pluronic acid was used as a dispersing agent to minimize aggregation of Englerin A.

### Data analysis

Data are presented as mean ± SEM, where n represents the number of independent experiments and the *N* represents the total number of wells of a 96‐well plate used for n experiments. For patch clamp experiments, *n* was the number of recordings from individual cells. For patch clamp experiments, currents were normalized to the maximum current. Data subjected to statistical analysis are based on at least five individual experiments (*n*). Data points in individual calcium imaging experiments were based on at least four replicates each. Student's t‐tests were used for comparisons between two sets of data and statistically significant differences are indicated when *P* < 0.05; no significant difference by NS. For IC_50_ determinations, data were normalized to the vehicle controls (DMSO or ethanol), and the Hill equation was fitted using Origin software (OriginLab, Northampton, MA, USA).

## Results

### Galangin inhibits TRPC5 channels overexpressed in HEK 293

**Figure 1:**
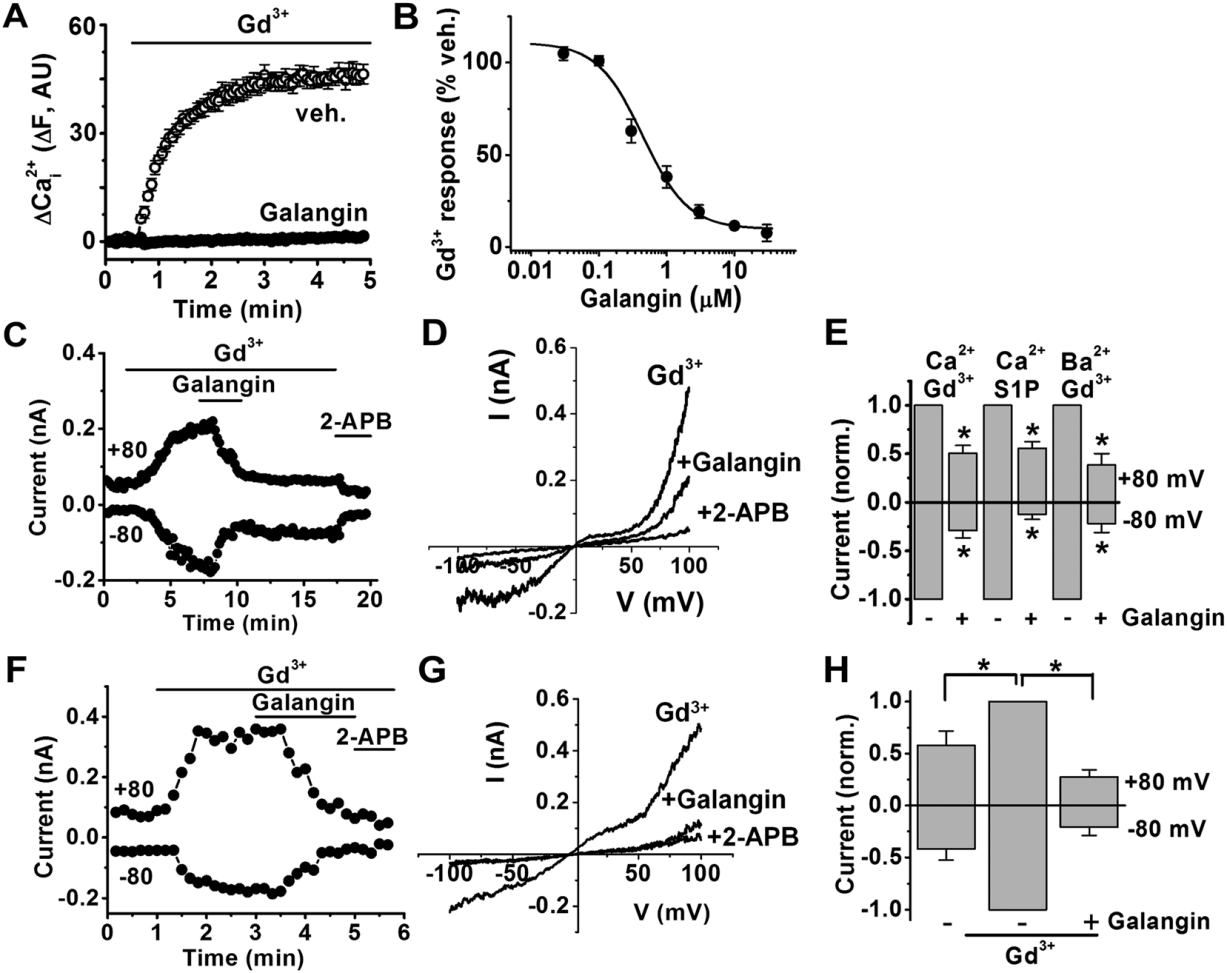
Galangin inhibits TRPC5. Recordings were from TRPC5‐expressing (Tet+) HEK 293, and extracellular Ca^2+^ was present at 1.5 mM unless indicated otherwise. (A) Free intracellular calcium ion (Ca^2+^i) concentration shown by fluo‐4 fluorescence intensity (F) in arbitrary units (AUs). Cells were incubated with 10 μM galangin or ethanol vehicle control (veh.) for 30 min before 50 μM Gd^3+^ was applied. (B) As for (A) but concentration–response data, showing an IC_50_ of the fitted Hill equation of 0.45 μM (*n/N* = 5/22). (C) Whole‐cell currents obtained during ramp changes in voltage from −100 to +100 mV every 10 s and during application of 30 μM Gd^3+^ and 10 μM galangin and then 75 μM 2‐aminoethoxydiphenylborate (2‐APB) alone. Ba^2+^ was present in the bath solution rather than Ca^2+^. (D) Example I‐Vs for the experiment in (C). (E) Normalized (norm.) data for whole‐cell currents evoked by 30 μM Gd^3+^ or 5 μM S1P in the absence (−) and presence (+) of 10 μM galangin. Ba^2+^ indicates when Ca^2+^ was substituted by barium ions in the bath solution. Currents were normalized to the amplitude prior to galangin application (*n* = 5, *n* = 5, *n* = 5). (F) Example outside‐out patch currents during application of 100 μM Gd^3+^, 10 μM galangin and 75 μM 2‐APB. (G) Example I‐Vs for the experiment in (F). (H) Normalized (norm.) data for outside‐out patch currents before and after application of 100 μM Gd^3+^ and after the addition of 10 μM galangin (*n* = 6). *P < 0.05.

We screened natural products from traditional Chinese medicines for effects on Ca^2+^ entry in HEK 293 cells overexpressing TRPC5 (Figure S1). Each natural product was pre‐incubated with cells for 30 min and maintained throughout each recording at 10 μM. During the recordings, the lanthanide gadolinium (50 μM Gd^3+^) was applied to stimulate the TRPC5‐mediated Ca^2+^ entry in the presence of each natural product. Through this screen, galangin was found to be inhibitory against the Gd^3+^‐evoked signal (Figure 1A). Galangin is from *Alpinia officinarum* and other members of the ginger family.

Galangin had a concentration‐dependent inhibitory effect against the Gd^3+^‐evoked Ca^2+^ signal, acting with an IC_50_ of 0.45 μM (Figure 1B). It was also effective against Gd^3+^‐evoked TRPC5‐mediated current in whole‐cell voltage clamp recordings (Figure 1C). Its effect occurred within 2 min and was not readily reversed on washout (Figure 1C). The TRPC5 current–voltage relationship (I‐V) characteristically showed inward rectification at negative voltages and outward rectification at positive voltages with a plateau between 0 and +40 mV, which gave an approximate inverted S‐shape and seat‐like effect at positive voltages (Figure 1D). This signature I‐V was suppressed by galangin, consistent with it acting as a TRPC5 channel inhibitor (Figure 1D). Subsequent application of the TRPC5 inhibitor 2‐aminoethoxydiphenylborate (75 μM) (Xu *et al*., 2005) further inhibited the current (Figure 1C and D). Galangin was effective whether Gd^3+^ activated the channel in the presence or absence of Ca^2+^ (Ca^2+^ was substituted by Ba^2+^), suggesting that its action was Ca^2+^ independent (Figure 1C–E). Galangin was also effective against TRPC5 current stimulated by S1P, suggesting that its effect was not restricted to inhibition of the Gd^3+^ effect (Figure 1e). Galangin inhibited the TRPC5‐mediated current evoked in excised outside‐out membrane patches, suggesting a relatively direct effect (Figure 1F–H). The data suggest that galangin is an inhibitor of TRPC5 channels.

### Galangin inhibits endogenous TRPC5‐containing channels

**Figure 2:**
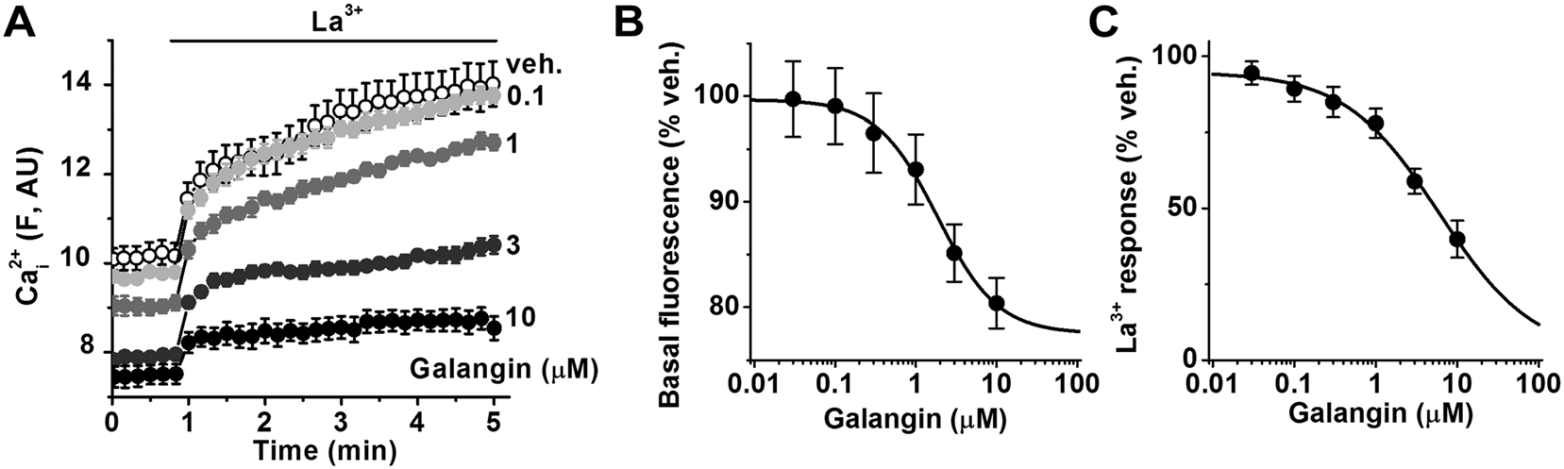
Galangin inhibits Ca^2+^ entry through endogenous channels in differentiated 3T3‐L1 cells. Intracellular Ca^2+^ was measured using fluo‐4. (A) Example data from a single 96‐well plate showing basal Ca^2+^ and then 20 μM La^3+^‐evoked Ca^2+^ entry in the presence of vehicle (veh.) and 0.1, 1, 3 and 10 μM galangin. (B, C) Summarized concentration–response data for experiments of the type shown in (A) for (B) basal Ca^2+^ and (C) La^3+^‐evoked Ca^2+^ entry (*n/N* = 10/59). The IC_50_s for inhibition of basal and La^3+^‐evoked Ca^2+^ entry were 1.85 and 6.05 μM respectively.

To determine if galangin also inhibits endogenous channels, we investigated differentiated 3T3‐L1 cells, which are a model of mature adipocytes and contain Ca^2+^ signals mediated by TRPC5‐containing channels (Sukumar *et al*., 2012). These endogenous channels exhibit constitutive activity leading to elevated basal intracellular Ca^2+^ concentration and further elevation in response to lanthanum (La^3+^), another lanthanide ion that was previously used in place of Gd^3+^ to activate TRPC5‐containing channels in 3T3‐L1 cells (Sukumar *et al*., 2012; Xu *et al*., 2008a). Galangin suppressed the basal Ca^2+^ signal and the La^3+^ response with estimated IC_50_s of 1.85 and 6.05 μM respectively (Figure 2A–C). The data suggest that galangin is an inhibitor of endogenous channels that contain TRPC5.

### Diverse effects of different but closely related natural flavonoids

**Figure 3:**
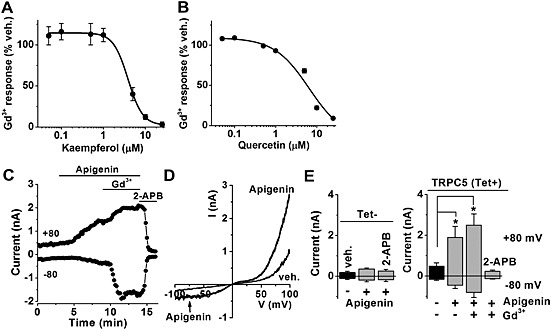
Negative or positive modulation of TRPC5 by natural flavonols. Recordings were from TRPC5‐expressing (Tet+) HEK 293 cells (A, B). Intracellular Ca^2+^ was measured using XRhod‐1. (A) Concentration–response data for kaempferol (IC_50_ 3.9 μM, *n/N* = 5/22). (B) Concentration–response data for quercetin (IC_50_ 6.5 μM, *n/N* = 5/25). (C–E) Whole‐cell data obtained from ramp changes in voltage from −100 to +100 mV every 10 s. (C) Example single‐cell recording showing responses to 10 μM apigenin and 30 μM Gd^3+^ and then 75 μM 2‐aminoethoxydiphenylborate (2‐APB) alone. (D) Example I‐Vs for the experiment in (C) [vehicle (veh.) indicates current before the application of apigenin]. (E) Mean current amplitudes for experiments of the type shown in (C) (*n* = 5 each). *P < 0.05.

Galangin is one in a series of compounds found in plants and commonly in diets and traditional remedies. A screen of natural flavonoids revealed that kaempferol and quercetin were inhibitors of Gd^3+^‐evoked Ca^2+^ entry in HEK 293 cells overexpressing TRPC5 but myricetin, apigenin and luteolin were not (Figure S2). Although kaempferol and quercetin were inhibitors, they were less potent than galangin (Figure 3A and B *cf*. Figure 1B). Apigenin had a stimulatory effect in Ca^2+^ measurement experiments (Figure S2) and was investigated further by whole‐cell voltage clamp recording. Apigenin was able to stimulate TRPC5‐mediated current, which could then be further enhanced by Gd^3+^ and blocked by 2‐aminoethoxydiphenylborate (Figure 3C–E). The apigenin‐activated current exhibited the characteristic I‐V shape of TRPC5, suggesting that it is indeed an activator of TRPC5 channels (Figure 3D). The data suggest that flavonoids inhibit, stimulate or have no effect on TRPC5 activity depending on small differences in substituent pattern.

### Identification of AM12 as a synthetic flavonol that inhibits Ca^2+^ entry evoked by Gd^3+^

**Figure 4:**
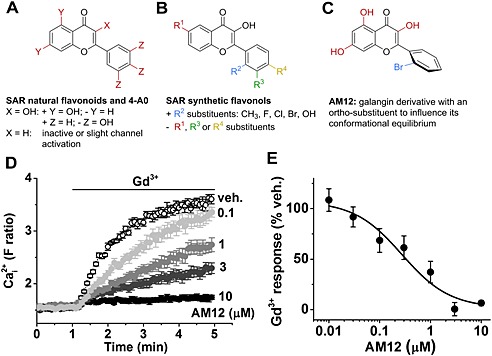
Design of AM12 and its inhibition of Gd^3+^‐evoked TRPC5 activity. (A–C) Structure–activity relationships (SARs) of natural flavonoids (A) and synthetic, mono‐substituted flavonols (B). Comparison of natural flavonoids with compound **4‐A0** reveals the influence of hydroxyl substituents on inhibitor potency. Comparison of compound series **4** (Figure S3) reveals that most TRPC5 inhibitors in this series (inhibition >50% at 10 μM) had an *ortho*‐substituted (R^2^) phenyl ring, while compounds with R^1^, R^3^ or R^4^ substituents had weak or variable stimulatory effects. (C) Flavonol AM12 combines the hydroxylation pattern of galangin, the strongest natural TRPC5 inhibitor found to date, with an *ortho*‐substituted phenyl ring. The structure of AM12 is drawn as a twisted conformer to highlight the proposed effect of an *ortho*‐Br substituent. (D, E) Intracellular Ca^2+^ was measured using fura‐2 in TRPC5‐expressing (Tet+) HEK 293. (D) Example data from a single 96‐well plate showing basal Ca^2+^ and then Gd^3+^‐evoked Ca^2+^ entry in the presence of vehicle (veh.) and 0.1, 1, 3 and 10 μM AM12. (E) Summarized concentration–response data for experiments of the type shown in (D) for Gd^3+^‐evoked Ca^2+^ entry (IC_50_ 0.28 μM, *n/N* = 5/30).

To further investigate structure–activity relationships of flavonols, a library of 41 mono‐substituted flavonols was synthesized using a two‐step procedure (Scheme S1) and screened at a concentration of 10 μM against Gd^3+^‐evoked Ca^2+^ entry in TRPC5 overexpressing HEK 293 cells (Figure S1). Guided by the results with natural and synthetic flavonols (Figure 4A and B), we designed AM12 (Figure 4c), which was synthesized in four steps (Scheme S2). AM12 inhibited the Gd^3+^‐evoked Ca^2+^ signal with an IC_50_ of 0.28 μM (Figure 4D and E). The data suggest that AM12 is slightly more potent than the natural product galangin as an inhibitor of the Gd^3+^‐evoked signal.

### AM12 inhibits TRPC1/4/5 channels relatively directly

**Figure 5:**
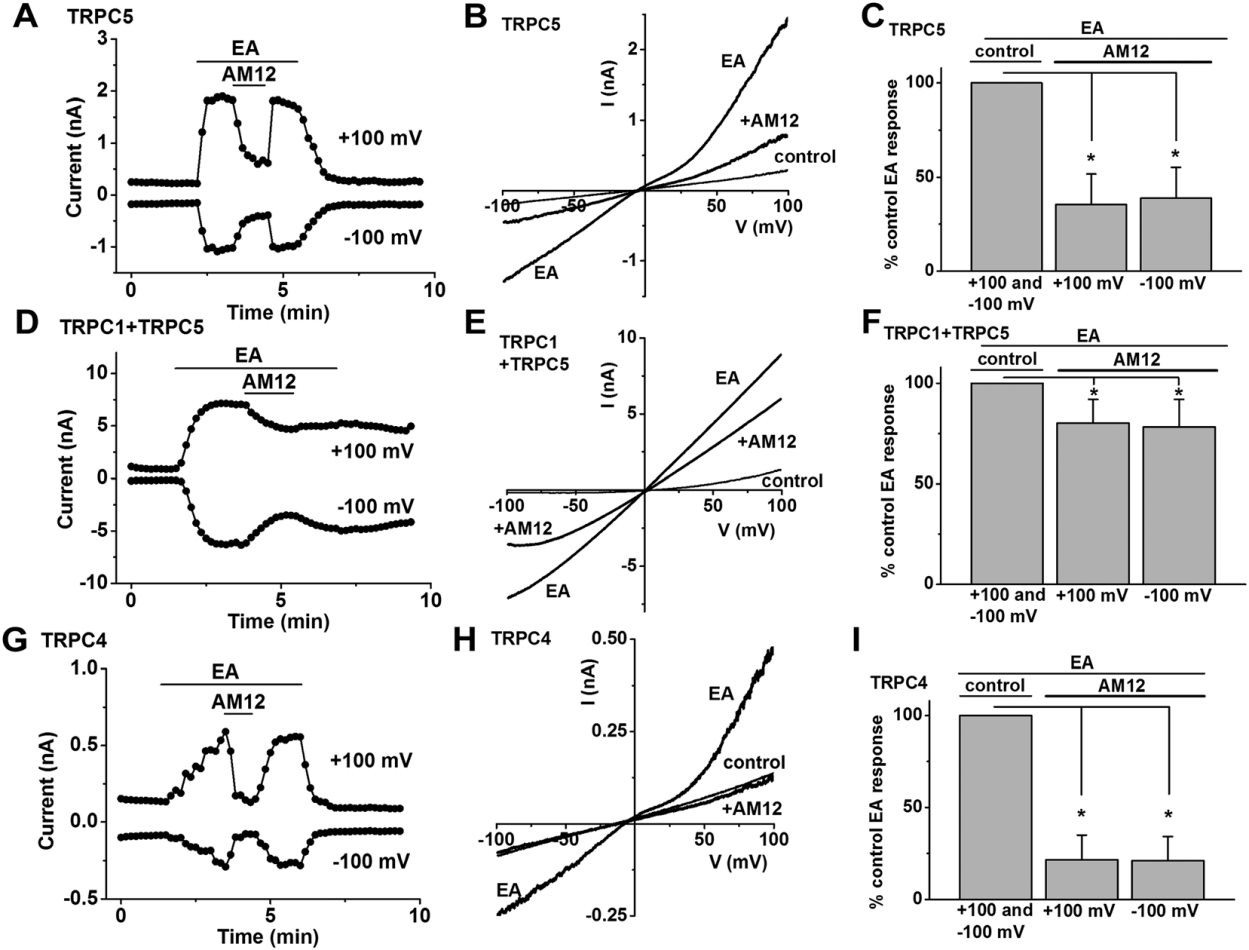
Inhibitory effect of AM12 on TRPC1/4/5 channels stimulated by (−)‐Englerin A (EA). Recordings were made from outside‐out membrane patches from TRPC5 Tet+ cells (A–C) or TRPC4 Tet+ cells (G–I) or from whole‐cell expressing SYFP2–TRPC1 and mTurquoise2–TRPC5 (D–F). (A) Example TRPC5 outside‐out patch currents during ramp changes in voltage from −100 to +100 mV every 10 s and application of 100 nM (−)‐EA and 5 μM AM12. (B) Example I‐Vs for the experiment in (A). (C) Mean normalized data for TRPC5 outside‐out patch currents evoked by 100 nM (−)‐EA and 5 μM AM12 (*n* = 5). (D) Example TRPC1–TRPC5 whole‐cell currents obtained during ramp changes in voltage from −100 to +100 mV every 10 s and during application of 100 nM (−)‐EA and 5 μM AM12. (E) Example I‐Vs for the experiment in (D). (F) Mean normalized data for TRPC1–TRPC5 whole‐cell currents evoked by 100 nM (−)‐EA and 5 μM AM12 (*n* = 5). (G) Example TRPC4 outside‐out patch currents during ramp changes in voltage from −100 to +100 mV every 10 s and application of 100 nM (−)‐EA and 5 μM AM12. (H) Example I‐Vs for the experiment in (G). (I) Mean normalized data for TRPC4 outside‐out patch currents evoked by 100 nM (−)‐EA and 5 μM AM12 (*n* = 5). *P < 0.05.

To investigate if AM12 might directly inhibit TRPC5, we used outside‐out patch recordings and bath‐applied AM12 to the extracellular face of the membrane. Moreover, to address the possibility that the effect of AM12 might be specific to Gd^3+^‐activated channel activity, we used an alternative, newly described, TRPC5 activator, (−)‐Englerin A (Akbulut *et al*., 2015). (−)‐Englerin A is considerably more potent and efficacious than Gd^3+^ (Figure 5A *cf*. Figure 1F). AM12 caused prompt inhibition of (−)‐Englerin A‐activated TRPC5 activity, and there was fast recovery on washout (Figure 5A–C). The average inhibition was ~65% at 5 μM AM12. AM12 also inhibited current through TRPC1–TRPC5 heteromeric channels, which were studied in whole‐cell recordings because of difficulty in obtaining outside‐out patches from TRPC1‐expressing cells (Figure 5D‐F). HEK 293 cells were transiently transfected with SYFP2–TRPC1 and mTurquoise2–TRPC5, and the expression of both proteins was detected by fluorescence microscopy. In whole‐cell patches of cells overexpressing both proteins and stimulated with (−)‐Englerin A, the characteristic seat‐like inflection of the TRPC5 I‐V was missing (Figure 5E cf. Figure 5B), which was consistent with the presence of heteromeric TRPC1–TRPC5 channels (Akbulut *et al*., 2015). AM12 was notably less effective against these heteromeric channels, giving only ~20% inhibition at 5 μM (Figure 5D‐F). Outside‐out patch recordings were also made from cells overexpressing TRPC4 homomeric channels, which are the most closely related to TRPC5 channels (Figure 5G–I). As with TRPC5 homomers, AM12 promptly inhibited TRPC4, and the average inhibition by 5 μM AM12 was ~80% (Figure 5G–I). The data suggest that AM12 inhibits TRPC5 and TRPC4 channels via a site accessible from the extracellular face of the membrane, acting directly on either the channel or a site closely associated with it. AM12 has an effect on heteromeric TRPC1–TRPC5 channels, but it is a relatively weak effect.

### Selectivity of AM12

**Figure 6:**
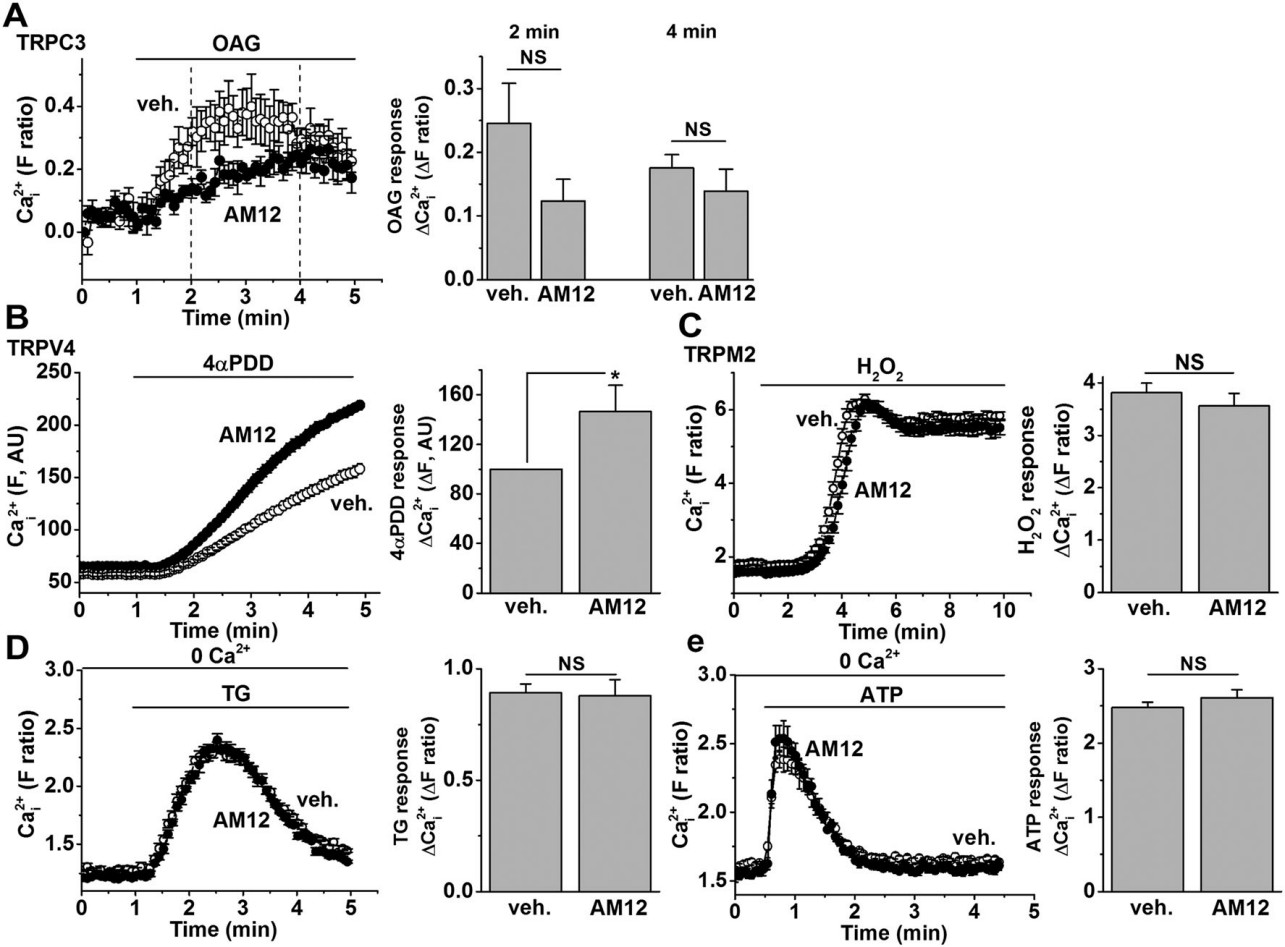
AM12 effects on TRPC3 channels, TRPV4 channels, TRPM2 channels and Ca^2+^ release. Intracellular Ca^2+^ was measured using fluo‐4 (B) or fura‐2 (A, C, D, E). (a) Cells were stably overexpressing TRPC3 and the TRPC3 agonist 1‐oleoyl‐2‐acetyl glycerol (OAG, 50 μM) was used to activate the channels in the presence of the vehicle control (veh.) or 10 μM AM12. On the left are example data from a single 96‐well plate and on the right are mean data at two different time points for multiple plates of this type (*n/N* = 5/26). (B) Cells were stably overexpressing TRPV4 and the TRPV4 agonist 4α‐phorbol 12,13‐didecanoate (4αPDD, 1 μM) was used to activate the channels in the presence of the veh. or 10 μM AM12. On the left are example data from a single 96‐well plate, and on the right are mean data for multiple plates of this type (*n/N* = 5/35). (C) Cells were overexpressing TRPM2, and the TRPM2 activator H_2_O_2_ (1 mM) was used to activate the channels in the presence of the veh. or 10 μM AM12. On the left are example data from a single 96‐well plate, and on the right are mean data for multiple plates of this type (*n/N* = 5/24). (D) Cells were non‐induced (Tet−) TRPC5 HEK cells in the absence of extracellular Ca^2+^. Thapsigargin (TG, 3 μM) was applied to release intracellular Ca^2+^ in the presence of the veh. or 10 μM AM12. On the left are example data from a single 96‐well plate, and on the right are mean data for multiple plates of this type (*n/N* = 5/32). (E) Cells were non‐induced (Tet−) TRPC5 HEK cells in the absence of extracellular Ca^2+^. A TP (100 μM) was applied to release intracellular Ca^2+^ in the presence of the veh. or 10 μM AM12. On the left are example data from a single 96‐well plate, and on the right are mean data for multiple plates of this type (*n/N* = 5/25). *P < 0.05; NS, not significant; AU, arbitrary unit.

At 10 μM, AM12 had a modest inhibitory effect on Ca^2+^ entry through TRPC3 channels in some recordings, but overall, the effect did not reach statistical significance (Figure 6A). There was a significant stimulatory effect on Ca^2+^ entry through TRPV4 channels, but no effect on TRPM2 channels (Figure 6A–C). AM12 had no effect on the endogenous Ca^2+^ release signal evoked by thapsigargin or ATP (Figure 6D and E). Thapsigargin causes Ca^2+^ release by inhibiting the smooth endoplasmic reticular Ca^2+^ ATPase, whereas A T P causes release via a GPCR and inositol 1,4,5‐trisphosphate production, via PLC activity. The data suggest AM12 has a degree of selectivity for TRPC5 and TRPC4 channels but is not completely specific.

### Stimulatory effect of AM12

**Figure 7:**
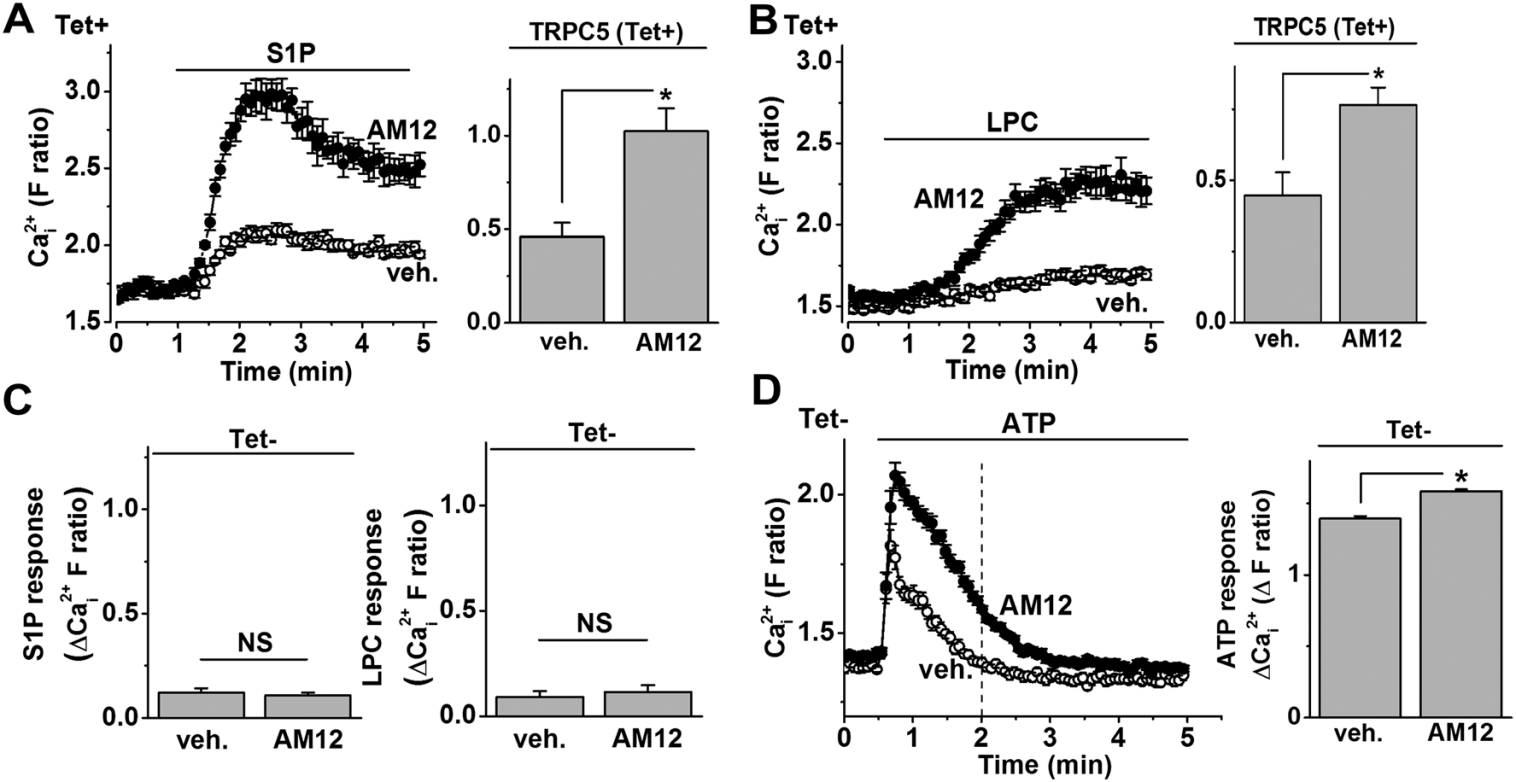
Stimulatory effects of AM12 on TRPC5. Intracellular Ca^2+^ was measured using fura‐2, and recordings were from TRPC5‐expressing (Tet+) HEK cells or control non‐induced (Tet−) cells. (A) Example data from Tet+ cells in a single 96‐well plate showing the response to 5 μM S1P in the presence of vehicle control (veh.) or 10 μM AM12 (left) and mean data for multiple plates of this type (*n/N* = 7/40). (B) Example data from Tet+ cells in a single 96‐well plate showing the response to 10 μM LPC in the presence of veh. or 10 μM AM12 (left) and mean data for multiple plates of this type (*n/N* = 9/56). (C) Mean data for the type of experiments shown in (A) (left; *n/N* = 6/31) and (B) (right; *n/N* = 5/20) for Tet− cells. (D) Example data from Tet− cells in a single 96‐well plate showing the response to 100 μM ATP in the presence of veh. or 10 μM AM12 (left) and mean data for multiple plates of this type (right; *n/N* = 5/30). *P < 0.05; NS, not significant.

Gd^3+^ and (−)‐Englerin A are not endogenous stimulators of TRPC5. Therefore, we next investigated the effect of AM12 against TRPC5 activity evoked by the physiological substance S1P. Unexpectedly, 10 μM AM12 stimulated rather than inhibited S1P‐evoked Ca^2+^ entry in HEK 293 cells overexpressing TRPC5 (Figure 7A). Similarly, TRPC5‐mediated Ca^2+^ entry evoked by the endogenous substance LPC was enhanced by AM12 (10 μM) (Figure 7B). In control cells without induction of TRPC5 expression (Tet− cells), there were no effects of AM12 (Figure 7C), but to further investigate if there were effects of AM12 in Tet‐ cells, we investigated the effect of ATP in the presence of extracellular Ca^2+^ and in the presence or absence of AM12 (Figure 7D). AM12 had no effect on A  TP‐ evoked Ca^2+^ release (Figure 6E) but enhanced the A  T P response in the presence of Ca^2+^ (Figure 7D). The data suggest that AM12 had a stimulatory effect on an endogenous Ca^2+^ entry mechanism. Stimulation of endogenous Ca^2+^ entry could potentially explain the stimulatory effects of AM12 in Tet+ cells (Figure 7A and B) because Ca^2+^‐mediated facilitation of TRPC5 channels has been described previously in these Tet+ cells (Hui *et al*., 2006). Nevertheless, it remained perplexing why AM12 did not act as an inhibitor when S1P and LPC were the agonists.

### Discussion and conclusions

Through a screen of a small number of natural products from traditional Chinese medicines, we found that the flavonol galangin is an inhibitor of lanthanide‐evoked activity of TRPC5 channels overexpressed in HEK 293 cells (IC_50_ 0.45 μM against TRPC5‐mediated Ca^2+^ entry). Galangin also inhibited Ca^2+^ entry through endogenous TRPC5‐containing channels as shown by studies of differentiated 3T3‐L1 cells, although it was 5–15 times less potent in these cells. Related natural flavonols were investigated, and two, kaempferol and quercetin, were also inhibitors of overexpressed TRPC5 but with less potency than galangin. Myricetin and luteolin lacked effect. Apigenin had the reverse effect, stimulating TRPC5. Investigation of a panel of mono‐substituted flavonols led to the design of compound AM12, which inhibited lanthanide‐evoked TRPC5 activity with an IC_50_ of 0.28 μM and showed a degree of selectivity as demonstrated by no significant inhibitory effects at 10 μM on Ca^2+^ release or Ca^2+^ entry mediated by TRPC3, TRPV4 or TRPM2 channels. However, unlike galangin, AM12 potentiated TRPC5 activity evoked by the physiological TRPC5 stimulators S1P and LPC, apparently lacking inhibitory effect in this situation. The data suggest complex modulator effects of natural and synthetic flavonoids on TRPC5 channels.

The modulator effects of natural flavonoids depended on variations in hydroxylation pattern, with inhibition being more prominent in the flavonols compared with the flavones (X = OH vs. H) (Table 1; Figure 4A). The inhibitory potency of natural flavonols decreased with increasing hydroxylation of the phenyl ring (Table 1; Figure 4A, Z = OH), and within this series, increased hydrophobicity (higher cLogP) seems to correlate with higher potency (Table 1). To investigate structure–activity relationships of flavonols as TRPC5 modulators, we made and tested a panel of mono‐substituted flavonols (Supporting Information). Most of the compounds had weak or variable stimulatory effects, and so, we focused on inhibition (Figure 4B). Most compounds that caused >50% inhibition contained ortho‐substituted phenyl rings (Figure S3 and Figure 4B, R^2^ = OH, CH_3_, F, Cl or Br), while most compounds with R^1^, R^3^ and R^4^ substituents (Figure 4B and Figure S3) had weak or mixed effects. For the mono‐substituted flavonols, no correlation between hydrophobicity and potency could be detected.

**Table 1:** Relationships between substituent pattern, hydrophobicity and potency of natural flavonoids and AM12^a^

Compound name	Compound structure	IC_50_ (μM)	cLogP^b^
AM12^c^	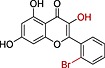	0.28	3.53
Galangin	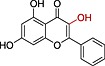	0.45	2.76
Kaempferol	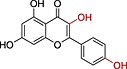	3.9	2.46
Quercetin	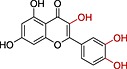	6.5	2.16
Myricetin	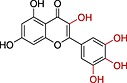	>>10	1.85
Apigenin^d^	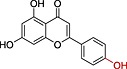	>>10	2.71
Luteolin	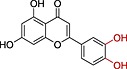	>>10	2.40

a TRPC5 inhibition was measured in TRPC5‐expressing (Tet+) HEK 293 cells treated with ethanolic solutions of flavonoids (10 mM stock solutions). TRPC5 was stimulated with 50 μM Gd^3+^, and Ca^2+^ entry was measured using the calcium‐reactive dye fluo‐4 unless stated otherwise. The positive controls consisted of HEK 293 cells overexpressing TRPC5 and stimulated with 50 μM Gd^3+^, treated with vehicle (ethanol) only. The negative controls consisted of recordings from (Tet−) cells, which do not overexpress TRPC5.

b cLogPs were calculated using chemicalize.org; ChemAxon: http://www.chemaxon.com; accessed October 2015.

c The calcium‐reactive dye fura‐2 was used in experiments with AM12.

d Apigenin acted as a stimulator of TRPC5 instead (Figure 3C–E).

The pronounced effect of *ortho*‐substituents (R^2^) as compared with *meta*‐substituents (R^3^) and *para*‐substituents (R^4^) on the flavonol phenyl ring might be attributed to their influence on the dihedral angle between the chromone and phenyl rings of the flavonol scaffold. *Ortho*‐substituents may cause restrictions on the conformational freedom of such biaryl scaffolds, favouring non‐planar conformers. Therefore, if TRPC5 inhibition is favoured by twisted flavonol conformers, *ortho*‐substituents may offer an energetic advantage by providing a conformational lock. To test this hypothesis, we designed and synthesized AM12 (Figure 4C), a galangin analogue with an *ortho*‐bromine substituent on the phenyl ring (see the Supporting Information for synthesis). AM12 was slightly more potent than galangin as an inhibitor of Gd^3+^‐evoked activity (Figure 4E *cf*. Figure 1B), which could be attributed to restriction of conformational freedom, but also fits the tentative correlation between hydrophobicity and potency as observed for natural flavonols (Table 1). However, the surprising stimulatory effect (Figure 7) and the more rapid reversibility of the inhibitory effect (Figure 5) suggested that subtle changes of flavonol substituents have a major impact on activity, mode of action and/or interaction with other calcium responses in the cell.

Based on simulations of molecular dynamics and functional membrane protein assays, it has been hypothesized that amphiphilic polyphenol phytochemicals – including the polyphenol resveratrol – localize to the membrane/solution interface, thereby reducing the energy required for bilayer adaptations perpendicular to the plane of the bilayer (Ingolfsson *et al*., 2014). Such an effect could alter conformational equilibria of membrane proteins whose function depends on conformational changes that are associated with bilayer perturbations. Quercetin and its metabolites have also been proposed to localize to the membrane/solution interface, enhancing their local concentrations and thereby their antioxidant effect on nearby lipids and membrane proteins (Kosinova *et al*., 2012). In addition, membrane penetration, and therefore local antioxidant effect, of quercetin derivatives was predicted to correlate with polarity of substituents (Kosinova *et al*., 2012). TRPC5 channels are susceptible to perturbations of the lipid bilayer as suggested by effects on channel activity of lipids depending on chain length and general anesthetics (Bahnasi *et al*., 2008; Flemming *et al*., 2006). Therefore, a plausible mechanism of action for flavonoids on TRPC5 is local perturbation of the bilayer, which then modulates channel activity.

The flavonols tested in this study all have a predicted pK_a_1 (first deprotonation) of ~6.4, which means that at physiological pH, their amphiphilic mono‐anions are the most prevalent species (Figure S4). In addition, the predicted octanol/water partition coefficients (cLogP) of galangin and AM12 (2.76 and 3.53 respectively) are in the same range as those of other polyphenol phytochemicals predicted to localize to the membrane/solution interface (Ingolfsson *et al*., 2014), and the apparent correlation between hydrophobicity within a subset of TRPC5‐inhibiting flavonols – but not flavones – (Table 1) is consistent with localization of the (substituted) phenyl ring of these compounds to a hydrophobic environment. However, the observation that subtle changes of flavonol substituent patterns can turn a poorly reversible inhibitor into a readily reversible inhibitor (galangin *cf*. AM12) and a TRPC5 inhibitor into a TRPC5 activator (galangin *cf.* apigenin) suggests that the mechanism of action of flavonols is more complex: activities may depend on membrane affinity, membrane localization and perturbation, and redox potential. In addition, distinct TRPC5 binding sites and/or flavonoid‐mediated modulation of calcium responses (including calcium release) through other proteins cannot be excluded. Moreover, the discovery of inhibitory and stimulatory effects of AM12 suggests a combination of effects.

Apigenin activated TRPC5, and it has previously been reported as a TRP activator, stimulating TRPV4 channels (Ma *et al*., 2012). A related isoflavone genistein also stimulated TRPC5 (Wong *et al*., 2010). Genistein has effects consistent with perturbation of the lipid bilayer (Ingolfsson *et al*., 2014).

The failure of AM12 to inhibit S1P‐evoked or LPC‐evoked TRPC5 activity is perplexing. It appears to be the case that AM12 had a separate stimulatory effect on another Ca^2+^ entry mechanism, which might then have facilitated TRPC5 activity. Nevertheless, despite such a possibility for facilitation, Gd^3+^‐evoked TRPC5 activity was inhibited by AM12, whereas S1P‐evoked and LPC‐evoked activities were not. These observations suggest that AM12 is not a direct inhibitor of the TRPC5 ion pore but a modulator that allosterically affects TRPC5 gating or the ion pore – by binding to TRPC5 or by perturbing the plasma membrane around TRPC5‐containing channels, or by acting more indirectly via a molecule closely associated with TRPC5. The data suggest the possibility for differential modulation of TRPC5 depending on its activation state; for example, it seems possible to inhibit lanthanide‐evoked and constitutive channel activity without affecting lipid‐evoked activity. We suspect that this complication is also involved in (−)‐Englerin A‐evoked activity. Although 5 μM AM12 inhibited (−)‐Englerin A‐evoked TRPC5 activity, the effect was on average less than that observed against Gd^3+^‐evoked activity, and in some recordings, the effect against (−)‐Englerin A‐evoked activity was surprisingly small, suggesting that a mixture of inhibitory and stimulatory actions of AM12 can affect the (−)‐Englerin A response.

The suppressive effect of galangin on lanthanum‐evoked Ca^2+^ entry in differentiated 3T3‐L1 cells suggests that it inhibits native TRPC5‐containing channels, which include other TRPC proteins such as TRPC1 (Sukumar *et al*., 2012). Consistent with the weaker effect of galangin on native channels (as compared with TRPC5 homomers), 5 μM AM12 had a small inhibitory effect on TRPC1–TRPC5 heteromeric channels (Figure 5D–F). Activity of galangin and other flavonoids in adipocytes has been previously reported. Ethanolic *A. officinarum* extract has been reported to inhibit adipocyte differentiation and high‐fat diet‐induced obesity in mice, and galangin (its major component), and had anti‐adipogenic effects in 3T3‐L1 cells (Jung *et al*., 2012). Genistein inhibited proliferation and subsequent differentiation of 3T3‐L1 cells (Harmon and Harp, 2001). Quercetin inhibited 3T3‐L1 cell growth and apoptosis (Hsu and Yen, 2006). TRPC5‐containing endogenous channels were up‐regulated in differentiated 3T3‐L1 cells, and inhibition of channel function *in vivo* by a dominant‐negative mutant TRPC5 raised circulating adiponectin levels, which is expected to have a cardioprotective effect (Sukumar *et al*., 2012). Therefore, flavonoids may act as natural regulators of adipocyte biology at least in part via modulation of Ca^2+^ entry through TRPC5‐containing channels, conferring a mechanism for integration with the environment via dietary intake. It should be noted, however, that flavonoids are not specific for TRPC5 channels. Galangin inhibited Ca_v_1.2 channels in smooth muscle, while quercetin, myricetin and kaempferol were stimulators (Saponara *et al*., 2011). Kaempferol and quercetin stimulated BK_Ca_ channels (Cogolludo *et al*., 2007; Xu *et al*., 2008b). Apigenin and quercetin inhibited GABA‐evoked ionic currents (Goutman *et al*., 2003).

In summary, the study suggests that naturally occurring flavonoids can modulate TRPC5 channels and that one consequence *in vivo* might be modulation of adiponectin secretion. The effects of synthetic flavonols on TRPC5 activity show that potency and mode of action of flavonols on TRPC5 channels can be tuned by subtle changes of substituent patterns and suggest future directions for the development of more potent flavonol‐based TRPC5 inhibitors. Nevertheless, effects of flavonols are difficult to predict, and their numerous biological activities render them potentially problematic for drug discovery efforts (Baell and Walters, 2014).

## Conflict of interest

The authors state no conflict of interest.

## Author contributions

J. N., A. M., H. J. G., M. S. A., L. A. W., M. M., S. Y. C., H. N. R., N. M. B., K. E. M., M. J. L., W. D. E., B. L. G., Y. Y., J. L. and K. M. performed the research. H. Y., C. W. G. F., D. J. B. and R. S. B. designed the research study. J. N., A. M., H. J. G., M. S. A., L. A. W., S. Y. C., H. N. R., B. L. G., J. L., K. M., D. J. B. and R. S. B. analysed the data. D. J. B. and R. S. B. wrote the paper.

## Supporting information


**Figure S1** Overview of chemicals from traditional Chinese medicines screened against Ca^2+^entry in HEK 293 cells overexpressing human TRPC5.
**Figure S2** Screen of natural flavonols against Ca^2+^ entry in HEK 293 cells over‐expressing human TRPC5. Intracellular Ca^2+^ was measured using XRhod‐1. Mean data comparing responses to 50 μM Gd^3+^ in the presence of 10 μM galangin, kaempferol, quercetin, myricetin,apigenin, luteolin or vehicle control (veh.) (*n/N*=3/12 each). Data were normalized to the Gd^3+^ response in vehicle and Tet+ cells.
**Figure S3** Overview of syntheticmono‐substituted flavonols that were screened for TRPC5 inhibition at 10 μM. Compounds that inhibited Gd^3+^‐evoked calcium entry in TRPC5‐expressing HEK293 cells by > 50% are highlighted in red.
**Figure S4** pKa1 values and structures of major microspecies (mm) of tested natural flavonoids and AM12 (predicted using Marvin Beans; downloaded from ChemAxon: http://www.chemaxon.com).
**Scheme S1** General synthetic route towards a library of mono‐substituted flavonols **4**. Aldol condensation of 2‐hydroxyacetophenones **1** with benzaldehydes **2** was followed by oxidative cyclisation of the intermediate chalcones **3** by use of an Algar‐Flynn‐Oyamada reaction.
**Scheme S2** Synthesis of synthetic flavonol AM12. Benzoic anhydride **6** was prepared from its corresponding benzoic acid **5**. Friedel‐Crafts acylation of phloroglucinol with acyl chloride **8** gave intermediate **9**. Combination of building blocks **6** and **9** in an Allan‐Robinson reaction followed by boron tribromide‐mediated demethylation afforded AM12. THF: tetrahydrofuran.

Supporting info item
