# Supplementary material for: Natural and synthetic flavonoid modulation of TRPC5 channels
Source: Br J Pharmacol. 2016 Jan 13;173(3):562–74. doi: 10.1111/bph.13387 (PMC4728423; doi:10.1111/bph.13387)
Supplement: Supplementary file 1 — Supporting info item [file BPH-173-562-s001.pdf]

Supporting Information

Natural and synthetic flavonoid modulation of TRPC5 channels

Jacqueline Naylor[1], Aisling Minard[2#], Hannah J Gaunt[1#], Mohamed S Amer[1,3], Lesley A Wilson[1], Marco Migliore[2], Sin Y Cheung[1], Hussein N Rubaiy[1], Nicola M Blythe[1], Katie E Musialowski[1], Melanie J Ludlow[1], William D Evans[2], Ben L Green[1], Hongjun Yang[4], Yun You[4], Jing Li[1], Colin W G Fishwick[2], Katsuhiko Muraki[5], David J Beech[1*], Robin S Bon[2*]

Schools of Medicine[1] and Chemistry[2], University of Leeds, Leeds, LS2 9JT, UK. [3]Clinical Physiology Department, Faculty of Medicine, Menoufiya University, Egypt. [4]Institute of Chinese Materia Medica, China Academy of Chinese Medical Sciences, Beijing, China. [5]School of Pharmacy, Aichi-Gakuin University, 1-100 Kusumoto, Chikusa, Nagoya 464-8650, Japan.

[#]These authors contributed equally. *To whom correspondence should be addressed: Dr Robin S Bon, School of Chemistry, Woodhouse Lane, University of Leeds, LS2 9JT, England, UK; r.bon@leeds.ac.uk; Tel +44 (0) 113 34 33196 or Prof David J Beech, School of Medicine, LIGHT Building, Clarendon Way, University of Leeds, Leeds, LS2 9JT, England, UK; d.j.beech@leeds.ac.uk; Tel +44 (0) 113 34 34323.

Contents

1. Supplementary Figures S1-S4 page 2

2. Synthetic details page 6

3. ^{1}H NMR spectra of synthetic flavonols page 23

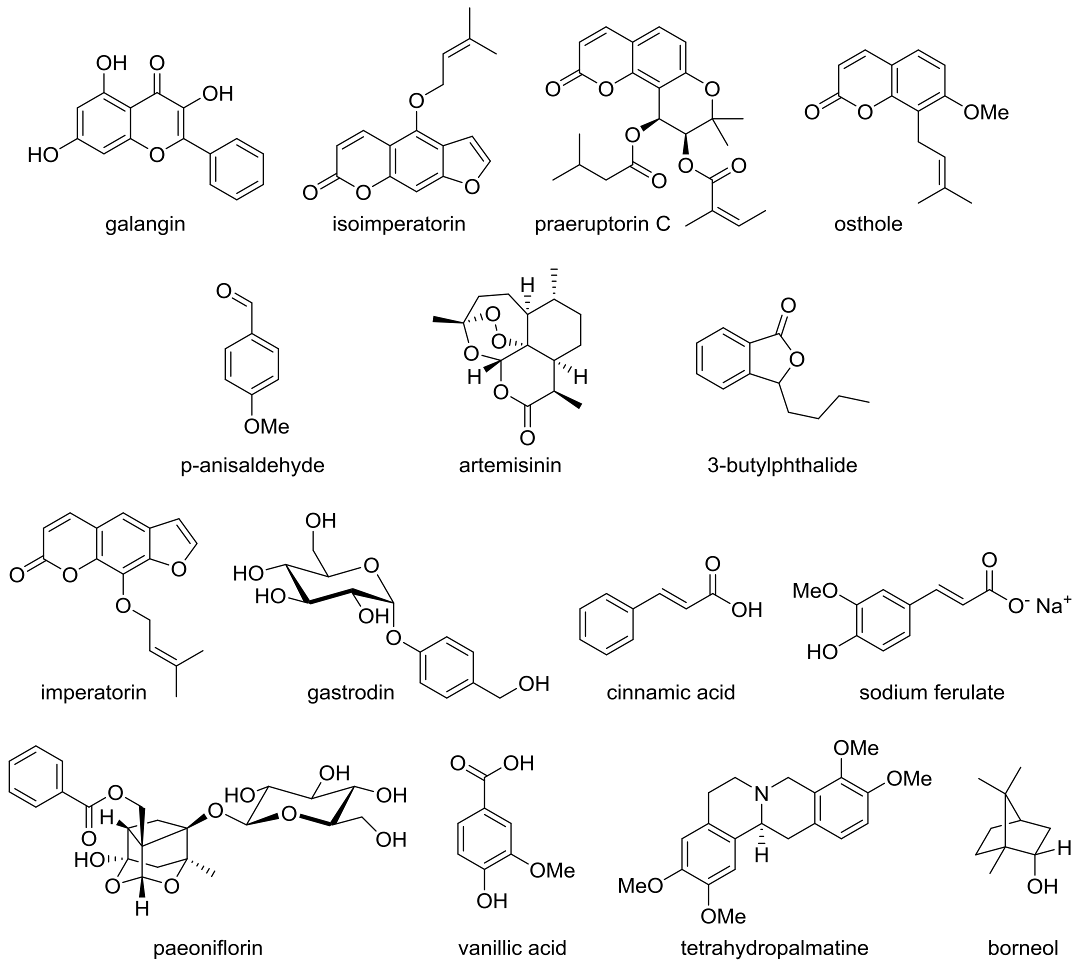

Figure S1. Overview of chemicals from traditional Chinese medicines screened against Ca^{2+} entry in HEK 293 cells over-expressing human TRPC5.

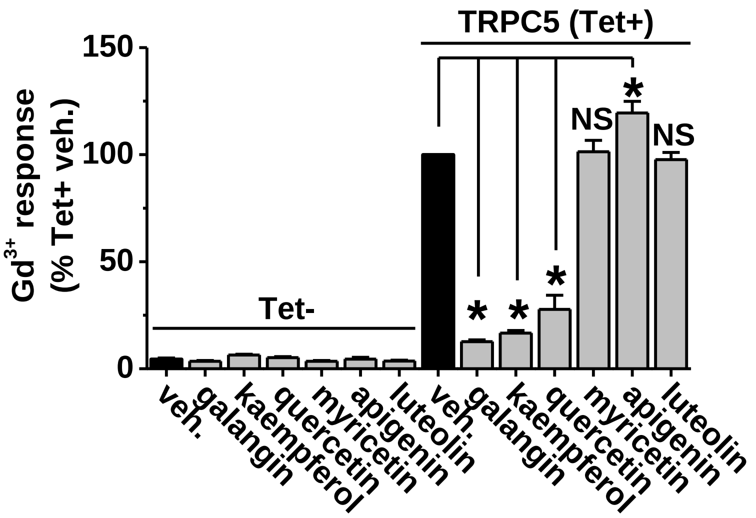

Figure S2. Screen of natural flavonols against Ca^{2+} entry in HEK 293 cells over-expressing human TRPC5. Intracellular Ca^{2+} was measured using XRhod-1. Mean data comparing responses to 50 µM Gd^{3+} in the presence of 10 µM galangin, kaempferol, quercetin, myricetin, apigenin, luteolin or vehicle control (veh.) (n/N=3/12 each). Data were normalized to the Gd^{3+} response in vehicle and Tet+ cells.

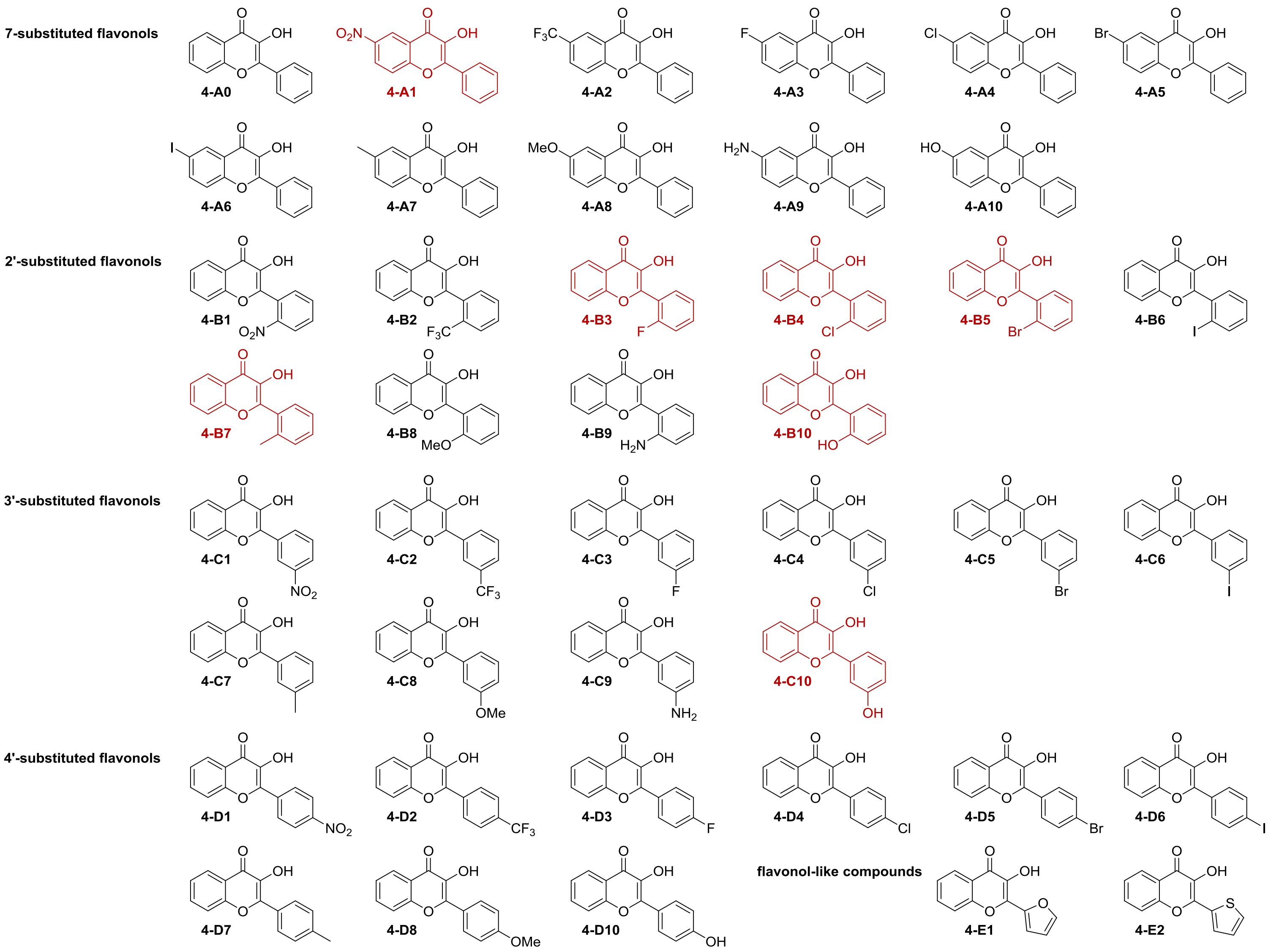

Figure S3. Overview of synthetic mono-substituted flavonols that were screened for TRPC5 inhibition at 10 µM. Compounds that inhibited Gd^{3+}-evoked calcium entry in TRPC5-expressing HEK293 cells by > 50% are highlighted in red.

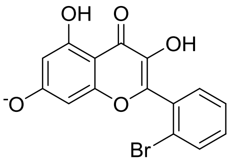

AM12-mm
pKa1 = 6.45

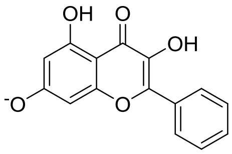

galangin-mm
pKa1 = 6.45

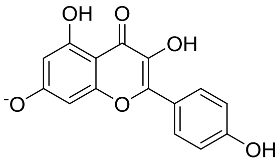

kaempferol-mm
pKa1 = 6.45

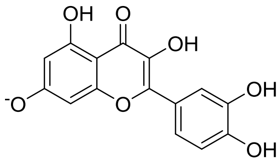

quercetin-mm
pKa1 = 6.45

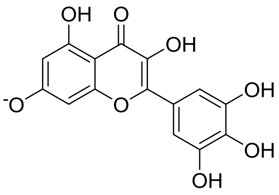

myricetin-mm
pKa1 = 6.45

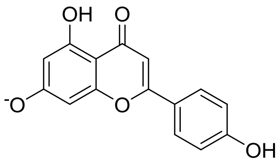

apigenin-mm
pKa1 = 6.64

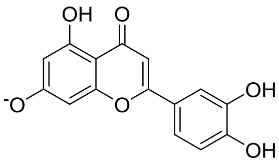

luteolin-mm
pKa1 = 6.64

Figure S4. pKa1 values and structures of major microspecies (mm) of tested natural flavonoids and AM12 (predicted using Marvin Beans; downloaded from ChemAxon: http://www.chemaxon.com).

Synthetic details

General experimental

Solvents and reagents were obtained from commercial suppliers and were used without further purification. Air- and moisture-sensitive reagents were handled under an atmosphere of dry nitrogen. ^{1}H and ^{13}C NMR spectra were recorded on a Bruker DPX300 and a Bruker Avance 500 using a deuterated dimethyl sulfoxide (DMSO-d_6) or deuterated chloroform ($CDCl_3$) as internal lock. Chemical shifts for ^{1}H and ^{13}C spectra were recorded in parts per million using the residual nondeuterated solvent as the internal standard (DMSO-d_6: ^{1}H = 2.50 ppm, ^{13}C = 39.52 ppm; $CDCl_3$: ^{1}H = 7.26 ppm, ^{13}C = 77.16 ppm). Data are reported as follows: chemical shift (ppm), multiplicity (indicated as br, broad signal; s, singlet; d, doublet; t, triplet; q, quartet; m, multiplet; and combinations thereof), coupling constant (J, Hz), and integrated intensity. Liquid chromatography mass spectra (LC-MS) were run on an Agilent 1200 LC system equipped with a Phenomenex Luna C18(2) 50 × 2 mm column, 5 μm particle size, on an acetonitrile/water gradient (5–95% acetonitrile, 0.1% formic acid, over 3 minutes) and a Bruker Daltonics HTCUltra™ system equipped with an Ion trap MS detector. High resolution mass spectra (HRMS) were recorded using a Bruker Daltonics micrOTOF or a Bruker MaXis Impact QqTOF using electron spray ionisation (ESI). All final compounds displayed ≥95% purity as determined by ^{1}H NMR analysis.

Synthesis of a library of mono-substituted flavonols

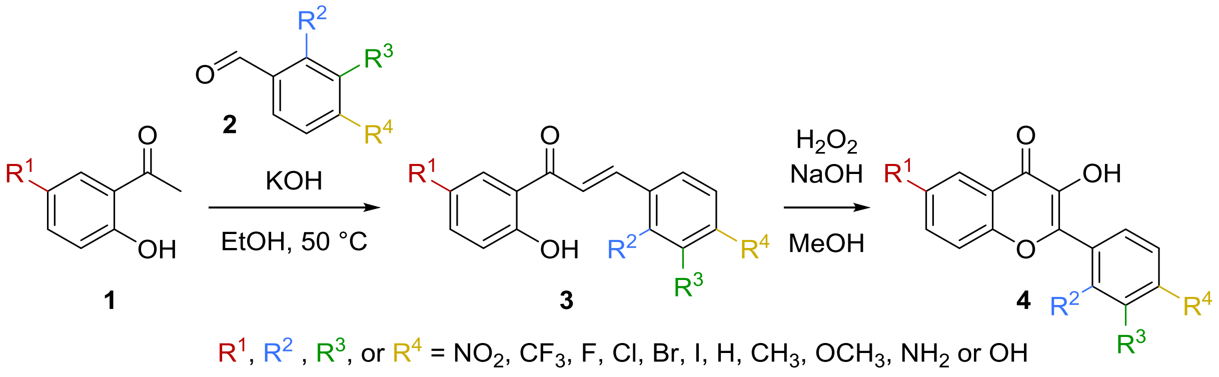

Scheme S1. General synthetic route towards a library of mono-substituted flavonols **4**. Aldol condensation of 2-hydroxyacetophenones **1** with benzaldehydes **2** was followed by oxidative cyclisation of the intermediate chalcones **3** by use of an Algar-Flynn-Oyamada reaction.

General procedure A. synthesis of flavonols 4.

A 2’-hydroxyacetophenone **1** (10 mmol) and a benzaldehyde **2** (10 mmol) were dissolved in ethanol (100 ml). Potassium hydroxide (1.12g, 20 mmol) was added and the mixture was stirred at 50 °C for 15 hours. After this time the pH was adjusted to 1-2 by the addition of conc. HCl and the precipitate formed was isolated by vacuum filtration to obtain the target chalcone **3** as a yellow solid that was used without any further purification.

To a solution of a chalcone **3** in methanol (100 ml), hydrogen peroxide 30% (5 ml, 50 mmol) and sodium hydroxide (1.2 g, 30 mmol) were added and the mixture was stirred at room temperature for 15 hours. Concentrated HCl was added until pH 1 and the precipitate formed was isolated by vacuum filtration to obtain the title compound as a solid, which was recrystallized from ethanol/water.

General Procedure B. reduction of nitro-substituted flavonols (R^n = NO_2) to form amine–substituted flavonols (R^n = NH_2):

To a solution of nitro-flavonol (1 mmol) in methanol, 10% Pd/C (53 mg, 0.05 mmol) was added and the mixture was hydrogenated for 5 hours under an atmosphere of hydrogen (balloon). The catalyst was filtered through a celite pad and the solvent was removed under reduced pressure to obtain the pure title compound as a solid.

General Procedure C. Demethylation of methoxy-substituted flavonols (R^n = OCH_3) to form hydroxyl-substituted flavonols (R^n = OH):

A solution of methoxy-flavonol (1 mmol) in dichloromethane was cooled to -20 °C. A solution of 1M BBr_3 in dichloromethane (5 ml, 5 mmol) was added drop-wise and the mixture was stirred for 15 hours. Methanol was carefully added to quench the reaction and the solvent removed under reduced pressure. The residue was taken up in methanol and the solid formed was isolated by vacuum filtration to obtain the pure title compound as a solid.

3-hydroxy-2-phenyl-chromen-4-one (4-A0)

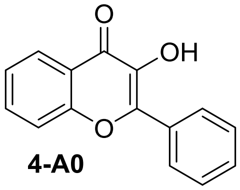

4-A0

Prepared according to general procedure A: white solid (51%). **^{1}H NMR** (500 MHz, $CDCl_3$) δ 8.33 – 8.23 (m, 3H), 7.71 (ddd, *J* = 8.5, 7.2, 1.4 Hz, 1H), 7.59 (d, *J* = 8.5 Hz, 1H), 7.54 (t, *J* = 7.6 Hz, 2H), 7.48 (t, *J* = 7.3 Hz, 1H), 7.42 (t, *J*

= 7.5 Hz, 1H), 7.09 (s, 1H). ^{13}**C NMR** (126 MHz, $CDCl_3$) δ 173.62 , 155.57 , 145.04 , 138.60 , 133.84 , 131.21 , 130.26 , 127.89 , 125.61 , 120.79 , 118.45 . **HRMS** $C_{15}H_{10}O_3$ requires 238.0630, found 238.0638 $[M+H]^+$.

3-hydroxy-6-nitro-2-phenyl-chromen-4-one (4-A1)

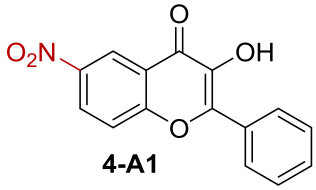

4-A1

Prepared according to general procedure A: yellow solid (43%). **1H NMR** (500 MHz, $CDCl_3$) δ 9.16 (d, *J* = 2.7 Hz, 1H), 8.54 (dd, *J* = 9.2, 2.7 Hz, 1H), 8.30 – 8.21 (m, 2H), 7.76 (d, *J* = 9.2 Hz, 1H), 7.57 (t, *J* = 7.3 Hz, 2H), 7.55 – 7.50 (m, 1H), 7.03 (s, 1H). ^{13}**C NMR** (126 MHz, $CDCl_3$) δ 172.99 , 156.17 , 144.25 , 143.70 , 136.77 , 131.05 , 128.61 , 128.16 , 126.78 , 125.29 , 121.56 , 115.99 . **HRMS** $C_{15}H_9NO_5$ requires 283.0481, found 283.0479 $[M+H]^+$.

3-hydroxy-2-phenyl-6-(trifluoromethyl)chromen-4-one (4-A2)

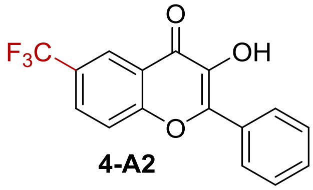

4-A2

Prepared according to general procedure A: white solid (51%). ^{1}**H NMR** (400 MHz, $CDCl_3$) δ 8.23 – 8.17 (m, 1H), 7.77 (dd, *J* = 7.1, 2.2 Hz, 3H), 7.57 (d, *J* = 7.5 Hz, 1H), 7.55 – 7.45 (m, 3H), 7.05 (s, 1H). ^{13}**C NMR** (126 MHz, $CDCl_3$) δ 172.99 , 155.32 , 143.70 , 136.77 , 131.45 (q, *J* = 3.7 Hz), 131.05 , 129.26 (q, *J* = 31.7 Hz), 128.61 , 128.16 , 124.98 , 123.09 – 122.83 (m), 123.67 (q, *J* = 267. 9 Hz), 120.63 (m). **HRMS** $C_{16}H_9F_3O_3$ requires 306.0504, found 306.0511 $[M+H]^+$.

6-fluoro-3-hydroxy-2-phenyl-chromen-4-one (4-A3)

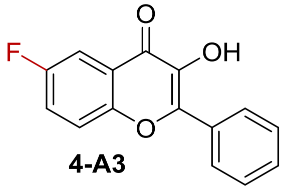

4-A3

Prepared according to general procedure A: white solid (46%). ^{1}**H NMR** (500 MHz, $CDCl_3$) δ 8.29 – 8.18 (m, 2H), 7.87 (dd, *J* = 8.1, 3.1 Hz, 1H), 7.59 (dd, *J* = 9.2, 4.1 Hz, 1H), 7.53 (t, *J* = 7.4 Hz, 2H), 7.51 – 7.46 (m, 1H), 7.43 (ddd, *J* = 9.2, 7.7, 3.1 Hz, 1H), 7.09 (s, 1H). ^{13}**C NMR** (75 MHz, $CDCl_3$) δ 172.97 (d, *J* = 2.6 Hz), 159.20 (d, *J* = 246.6 Hz), 151.89 , 145.57 , 138.35 , 130.95 , 130.52 , 128.76 , 127.95 , 122.32 (d, *J* = 25.9 Hz), 121.76 (d, *J* = 8.1 Hz), 120.55 (d, *J* = 8.3 Hz), 110.07 (d, *J* = 23.7 Hz). **HRMS** $C_{15}H_9FO_3$ requires 256.0536, found 256.0543 $[M+H]^+$.

6-chloro-3-hydroxy-2-phenyl-chromen-4-one (4-A4)

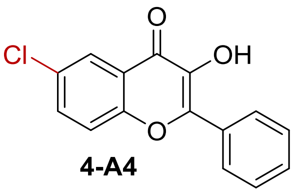

4-A4

Prepared according to general procedure A: white solid (62%). **^{1}H NMR** (500 MHz, $CDCl_3$) δ 8.25 – 8.22 (m, 2H), 8.21 (d, *J* = 2.5 Hz, 1H), 7.63 (dd, *J* = 9.0, 2.5 Hz, 1H), 7.54 (t, *J* = 8.3 Hz, 3H), 7.48 (t, *J* = 7.3 Hz, 1H), 7.06 (s, 1H). **^{13}C NMR** (75 MHz, $CDCl_3$) δ 172.57 , 153.87 , 145.56 , 138.73 , 134.04 , 130.87 , 130.65 , 130.58 , 128.79 , 127.95 , 124.85 , 121.73 , 120.14 . **HRMS** $C_{15}H_9ClO_3$ requires 272.0240, found 272.0234 $[M+H]^+$.

6-bromo-3-hydroxy-2-phenyl-chromen-4-one (4-A5)

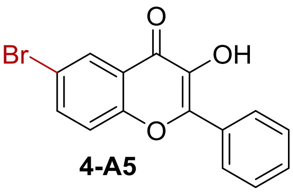

4-A5

Prepared according to general procedure A: white solid (75%). **^{1}H NMR** (500 MHz, $CDCl_3$) δ 8.38 (d, *J* = 2.4 Hz, 1H), 8.30 – 8.21 (m, 2H), 7.77 (dd, *J* = 8.9, 2.4 Hz, 1H), 7.54 (t, *J* = 7.4 Hz, 2H), 7.51 – 7.46 (m, 2H), 7.04 (s, 1H). **^{13}C NMR** (75 MHz, $CDCl_3$) δ 172.42 , 154.28 , 145.56 , 138.77 , 136.74 , 130.86 , 130.60 , 128.80 , 128.10 , 127.95 , 122.17 , 120.35 , 118.02 . **HRMS** $C_{15}H_9BrO_3$ requires 315.9735, found 315.9742 $[M+H]^+$.

3-hydroxy-6-iodo-2-phenyl-chromen-4-one (4-A6)

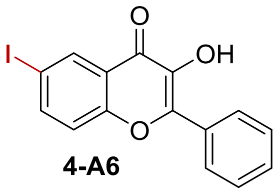

4-A6

Prepared according to general procedure A: white solid (35%). **^{1}H NMR** (500 MHz, $CDCl_3$) δ 8.58 (d, *J* = 2.1 Hz, 1H), 8.26 – 8.20 (m, 2H), 7.94 (dd, *J* = 8.9, 2.2 Hz, 1H), 7.56 – 7.51 (m, 2H), 7.51 – 7.45 (m, 1H), 7.35 (d, *J* = 8.9 Hz, 1H), 7.04 (s, 1H). **^{13}C NMR** (75 MHz, $CDCl_3$) δ 172.18 , 154.90 , 145.54 , 142.24 , 138.80 , 134.46 , 130.87 , 130.59 , 128.80 , 127.94 , 122.59 , 120.44 , 88.25 . **HRMS** $C_{15}H_9IO_3$ requires 363.9596, found 363.9608 $[M+H]^+$.

3-hydroxy-6-methyl-2-phenyl-chromen-4-one (4-A7)

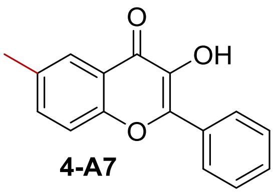

4-A7

Prepared according to general procedure A: white solid (71%). **^{1}H NMR** (500 MHz, $CDCl_3$) δ 8.31 – 8.21 (m, 3H), 8.03 (s, 1H), 7.61 – 7.43 (m, 4H), 7.05 (s, 1H), 2.48 (s, 3H). **^{13}C NMR** (75 MHz, $CDCl_3$) δ 173.56 , 154.00 , 144.91 , 138.57 , 135.26 , 134.63 , 131.38 , 130.22 , 128.71 , 127.88 , 124.71 , 120.50 , 118.18 , 21.07 . **HRMS** $C_{16}H_{12}O_3$ requires 252.0786, found 252.0790 $[M+H]^+$.

3-hydroxy-6-methoxy-2-phenyl-chromen-4-one (4-A8)

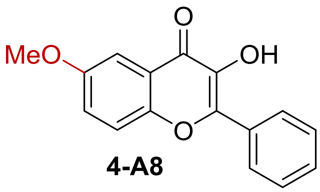

4-A8

Prepared according to general procedure A: white solid (55%). **^{1}H NMR** (500 MHz, $CDCl_3$) δ 8.28 – 8.21 (m, 2H), 7.57 (d, *J* = 3.1 Hz, 1H), 7.56 – 7.50 (m, 3H), 7.49 – 7.45 (m, 1H), 7.31 (dd, *J* = 9.2, 3.1 Hz, 1H), 7.03 (s, 1H), 3.92 (s, 3H). **^{13}C NMR** (75 MHz, $CDCl_3$) δ 173.25 , 156.68 , 150.75 , 144.95 , 138.30 , 131.35 , 130.25 , 128.74 , 127.87 , 124.61 , 121.27 , 119.90 , 104.04 , 56.11 . **HRMS** $C_{16}H_{12}O_4$ requires 268.0736, found 268.0732 $[M+H]^+$.

6-amino-3-hydroxy-2-phenyl-chromen-4-one (4-A9)

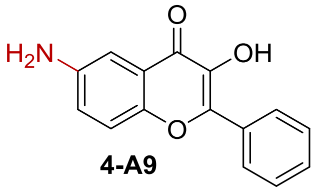

4-A9

Prepared from **4-A1** according to general procedure B: yellow solid (95%). **^{1}H NMR** (500 MHz, DMSO-d_6) δ 9.26 (s, 1H), 8.27 – 8.10 (m, 3H), 7.54 (td, *J* = 7.0, 1.6 Hz, 2H), 7.51 – 7.44 (m, 1H), 7.17 (d, *J* = 2.8 Hz, 1H), 7.09 (dd, *J* = 8.9, 2.8 Hz, 1H), 5.47 (s, 2H). **^{13}C NMR** (75 MHz, DMSO-d_6) δ 172.59 , 147.30 , 145.80 , 144.41 , 138.16 , 131.67 , 129.48 , 128.39 , 127.42 , 122.29 , 122.07 , 118.79 , 104.07 . **HRMS** $C_{15}H_{11}NO_3$ requires 253.0739, found 253.0732 $[M+H]^+$.

3,6-dihydroxy-2-phenyl-chromen-4-one (4-A10)

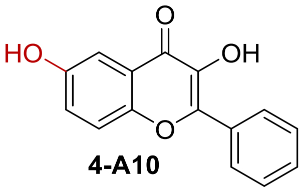

4-A10

Prepared from **4-A8** according to general procedure C: pink solid (23%). **^{1}H NMR** (500 MHz, DMSO-d_6) δ 9.97 (s, 1H), 9.41 (s, 1H), 8.23 – 8.15 (m, 2H), 7.62 (d, *J* = 9.1 Hz, 1H), 7.55 (t, *J* = 7.6 Hz, 2H), 7.48 (t, *J* = 7.3 Hz, 1H), 7.37 (d, *J* = 3.0 Hz, 1H), 7.26 (dd, *J* = 9.1, 3.0 Hz, 1H). **^{13}C NMR** (75 MHz, DMSO-d_6) δ 172.52, 154.06, 148.64, 144.82, 138.23, 131.40, 129.59, 128.47, 127.52, 123.33, 122.02, 119.84, 106.71 . **HRMS** $C_{15}H_{10}O_4$ requires 254.0579, found 254.0571 $[M+H]^+$.

3-hydroxy-2-(2-nitrophenyl)chromen-4-one (4-B1)

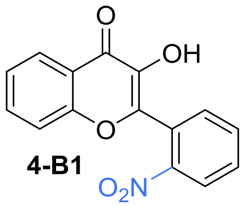

4-B1

Prepared according to general procedure A: yellow solid (23%). **^{1}H NMR** (500 MHz, $CDCl_3$) δ 8.28 (dd, *J* = 8.0, 1.4 Hz, 1H), 8.14 – 8.06 (m, 1H), 7.93 (dd, *J* = 7.7, 1.1 Hz, 1H), 7.78 (td, *J* = 7.6, 1.0 Hz, 1H), 7.74 – 7.65 (m, 2H), 7.50 – 7.42 (m, 2H), 6.60 (s, 1H). **^{13}C NMR** (75 MHz, $CDCl_3$) δ 172.31 , 153.68 , 148.28 , 142.97 , 138.94 , 130.77 – 130.72 (m), 130.69 , 129.37 , 125.97 , 125.87 , 125.74 , 125.65 , 122.56 , 115.40 . **HRMS** $C_{15}H_9NO_5$ requires 283.0481, found 283.0475 $[M+H]^+$.

3-hydroxy-2-[2-(trifluoromethyl)phenyl]chromen-4-one (4-B2)

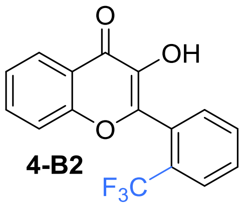

4-B2

Prepared according to general procedure A: white solid (36%). **^{1}H NMR** (500 MHz, $CDCl_3$) δ 8.29 (dd, *J* = 8.1, 1.3 Hz, 1H), 7.87 (d, *J* = 7.8 Hz, 1H), 7.83 (d, *J* = 7.6 Hz, 1H), 7.74 – 7.67 (m, 2H), 7.64 (t, *J* = 7.6 Hz, 1H), 7.50 (d, *J* = 8.5 Hz, 1H), 7.44 (t, *J* = 7.6 Hz, 1H), 6.69 (s, 1H). **^{13}C NMR** (75 MHz, $CDCl_3$) δ 173.59 , 155.85 , 145.21 , 138.70 , 134.02 , 132.01 , 131.88 , 130.61 , 129.44 (q, *J* = 31.7 Hz), 128.67 (q, *J* = 1.9 Hz), 127.33 (q, *J* = 5.1 Hz), 125.69 , 124.88 , 123.77 (q, *J* = 273.9 Hz), 121.34 , 118.57 . **HRMS** $C_{16}H_9F_3O_3$ requires 306.0504, found 306.0501 $[M+H]^+$.

2-(2-fluorophenyl)-3-hydroxy-chromen-4-one (4-B3)

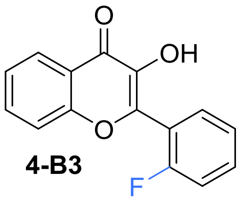

4-B3

Prepared according to general procedure A: white solid (49%). **^{1}H NMR** (500 MHz, $CDCl_3$) δ 8.28 (d, *J* = 8.0 Hz, 1H), 7.81 (td, *J* = 7.7, 1.6 Hz, 1H), 7.70 (ddd, *J* = 8.5, 7.3, 1.5 Hz, 1H), 7.57 – 7.46 (m, 2H), 7.43 (t, *J* = 7.5 Hz, 1H), 7.30 (t, *J* = 7.6 Hz, 1H), 7.27 – 7.20 (m, 1H), 6.75 (s, 1H). **^{13}C NMR** (75 MHz, $CDCl_3$) δ 173.40 , 160.06 (d, *J* = 255.3 Hz), 156.09 , 142.91 , 139.15 , 133.85 , 132.45 (d, *J* = 8.5 Hz), 131.02 (d, *J* = 2.3 Hz), 125.69 , 124.73 , 124.23 (d, *J* = 3.6 Hz), 121.21 , 118.95 (d, *J* = 13.3 Hz), 118.63 , 116.67 (d, *J* = 21.4 Hz) . **HRMS** $C_{15}H_9FO_3$ requires 256.0536, found 256.0533 $[M+H]^+$.

2-(2-chlorophenyl)-3-hydroxy-chromen-4-one (4-B4)

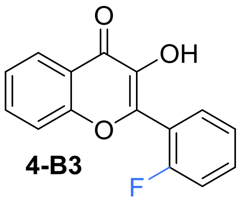

4-B3

Prepared according to general procedure A: white solid (67%). **^{1}H NMR** (500 MHz, $CDCl_3$) δ 8.30 (dd, *J* = 8.1, 1.5 Hz, 1H), 7.70 (ddd, *J* = 8.8, 7.2, 1.7 Hz, 1H), 7.67 (dd, *J* = 7.5, 1.9 Hz, 1H), 7.56 (dd, *J* = 7.9, 1.2 Hz, 1H), 7.53 (d, *J* = 8.5 Hz, 1H), 7.48 – 7.39 (m, 3H), 6.80 (s, 1H). **^{13}C NMR** (75 MHz, $CDCl_3$) δ 173.59 , 156.02 , 145.31 , 139.05 , 134.00 , 133.85 , 131.71 , 131.64 , 130.46 , 129.73 , 126.83 , 125.72 , 124.72 , 121.43 , 118.58 . **HRMS** $C_{15}H_9ClO_3$ requires 272.0240, found 272.0242 $[M+H]^+$.

2-(2-bromophenyl)-3-hydroxy-chromen-4-one (4-B5)

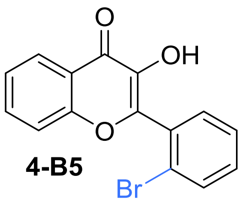

4-B5

Prepared according to general procedure A: white solid (81%). **^{1}H NMR** (500 MHz, $CDCl_3$) δ 8.30 (d, *J* = 7.9 Hz, 1H), 7.75 (d, *J* = 8.1 Hz, 1H), 7.71 (t, *J* = 7.8 Hz, 1H), 7.64 (dd, *J* = 7.6, 1.5 Hz, 1H), 7.54 (d, *J* = 8.5 Hz, 1H), 7.50 – 7.41 (m, 2H), 7.37 (td, *J* = 7.9, 1.6 Hz, 1H), 6.73 (s, 1H). **^{13}C NMR** (75 MHz, $CDCl_3$) δ 173.98 ,

156.27 , 146.70 , 139.13 , 134.18 , 133.97 , 132.21 , 132.09 , 132.04 , 127.74 , 126.03 , 125.06 , 123.47 , 121.80 , 118.91 . **HRMS** $C_{15}H_9BrO_3$ requires 315.9735, found 315.9731 $[M+H]^+$.

3-hydroxy-2-(2-iodophenyl)chromen-4-one (4-B6)

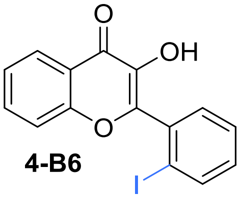

4-B6

Prepared according to general procedure A: white solid (57%). **^{1}H NMR** (500 MHz, $CDCl_3$) δ 8.30 (dd, *J* = 8.1, 1.5 Hz, 1H), 8.02 (dd, *J* = 8.0, 1.0 Hz, 1H), 7.72 (ddd, *J* = 8.7, 7.1, 1.7 Hz, 1H), 7.59 (dd, *J* = 7.7, 1.6 Hz, 1H), 7.56 (d, *J* = 8.4 Hz, 1H), 7.51 (td, *J* = 7.6, 1.1 Hz, 1H), 7.45 (ddd, *J* = 8.0, 7.2, 0.9 Hz, 1H), 7.21 (td, *J* = 7.8, 1.7 Hz, 1H), 6.60 (s, 1H). **^{13}C NMR** (75 MHz, $CDCl_3$) δ 174.02 , 156.19 , 148.39 , 140.42 , 138.70 , 135.96 , 134.21 , 132.02 , 131.75 , 128.50 , 126.00 , 125.11 , 121.82 , 118.95 , 97.26 . **HRMS** $C_{15}H_9IO_3$ requires 363.9596, found 363.9589 $[M+H]^+$.

3-hydroxy-2-(o-tolyl)chromen-4-one (4-B7)

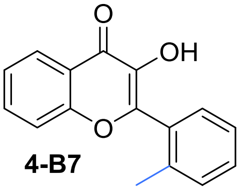

4-B7

Prepared according to general procedure A: white solid (68%). **^{1}H NMR** (500 MHz, $CDCl_3$) δ 8.31 (dd, *J* = 8.1, 1.4 Hz, 1H), 7.70 (tt, *J* = 8.1, 1.3 Hz, 1H), 7.61 (dd, *J* = 7.6, 1.5 Hz, 1H), 7.52 (dd, *J* = 8.6, 1.2 Hz, 1H), 7.46 – 7.40 (m, 2H), 7.38 – 7.32 (m, 2H), 6.63 (s, 1H), 2.42 (s, 3H). **^{13}C NMR** (75 MHz, $CDCl_3$) δ 175.58 , 158.18 , 150.11 , 140.71 , 139.99 , 135.84 , 133.14 , 132.71 , 132.31 , 132.10 , 128.05 , 127.89 , 126.86 , 123.56 , 120.67 , 22.38 . **HRMS** $C_{16}H_{12}O_3$ requires 252.0786, found 252.0787 $[M+H]^+$.

3-hydroxy-2-(2-methoxyphenyl)chromen-4-one (4-B8)

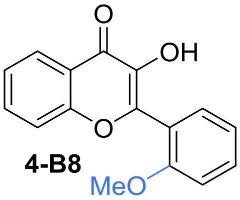

4-B8

Prepared according to general procedure A: white solid (68%). **^{1}H NMR** (500 MHz, $CDCl_3$) δ 8.29 (d, *J* = 7.7 Hz, 1H), 7.67 (t, *J* = 7.5 Hz, 1H), 7.59 (d, *J* = 7.3 Hz, 1H), 7.56 – 7.46 (m, 2H), 7.41 (t, *J* = 7.4 Hz, 1H), 7.11 (t, *J* = 7.5 Hz, 1H), 7.07 (d, *J* = 8.4 Hz, 1H), 6.49 (s, 1H), 3.88 (s, 3H). **^{13}C NMR** (75 MHz, $CDCl_3$) δ 173.51 , 159.71 , 155.46 , 144.66 , 138.58 , 133.69 , 132.36 , 129.65 , 125.50 , 124.55 , 120.64 , 120.28 , 118.33 , 116.02 , 113.28 , 55.45 . **HRMS** $C_{16}H_{12}O_4$ requires 268.0736, found 268.0739 $[M+H]^+$.

2-(2-aminophenyl)-3-hydroxy-chromen-4-one (4-B9)

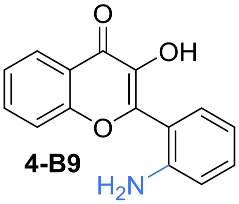

4-B9

Prepared from **4-B1** according to general procedure B: yellow solid (87%). **^{1}H NMR** (500 MHz, DMSO-d_6) δ 9.25 (s, 1H), 8.13 (dd, *J* = 8.0, 1.3 Hz, 1H), 7.77

(ddd, *J* = 8.6, 7.4, 1.5 Hz, 1H), 7.63 (d, *J* = 8.5 Hz, 1H), 7.46 (ddd, *J* = 8.1, 7.1, 1.0 Hz, 1H), 7.37 (d, *J* = 6.5 Hz, 1H), 7.20 (ddd, *J* = 8.5, 7.3, 1.7 Hz, 1H), 6.82 (d, *J* = 7.9 Hz, 1H), 6.68 (td, *J* = 7.6, 1.1 Hz, 1H), 5.31 (s, 2H). **^{13}C NMR** (75 MHz, DMSO-d_6) δ 172.60 , 155.13 , 147.97 , 146.68 , 138.17 , 133.23 , 130.82 , 130.23 , 124.70 , 124.23 , 121.87 , 118.42 , 116.15 , 115.80 , 115.38 . **HRMS** $C_{15}H_{11}NO_3$ requires 253.0739, found 253.0744 $[M+H]^+$.

3-hydroxy-2-(2-hydroxyphenyl)chromen-4-one (4-B10)

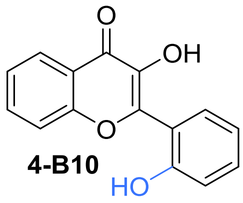

4-B10

Prepared from **4-B8** according to general procedure C: pink solid (32%). **^{1}H NMR** (500 MHz, DMSO-d_6) δ 9.78 (s, 1H), 8.99 (s, 1H), 8.14 (dd, *J* = 8.0, 1.4 Hz, 1H), 7.76 (ddd, *J* = 8.6, 7.1, 1.7 Hz, 1H), 7.61 (d, *J* = 8.4 Hz, 1H), 7.50 – 7.42 (m, 2H), 7.35 (ddd, *J* = 8.3, 7.4, 1.7 Hz, 1H), 6.99 (dd, *J* = 8.3, 1.1 Hz, 1H), 6.93 (td, *J* = 7.5, 1.0 Hz, 1H). **^{13}C NMR** (75 MHz, DMSO-d_6) δ 173.10 , 155.76 , 155.42 , 147.88 , 139.24 , 133.71 , 131.78 , 131.25 , 125.18 , 124.70 , 122.35 , 119.07 , 118.73 , 118.66 , 116.78 . **HRMS** $C_{15}H_{10}O_4$ requires 254.0579, found 254.0576 $[M+H]^+$.

3-hydroxy-2-(3-nitrophenyl)chromen-4-one (4-C1)

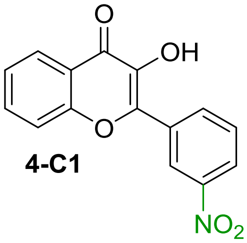

4-C1

Prepared according to general procedure A: yellow solid (59%). **^{1}H NMR** (500 MHz, $CDCl_3$) δ 9.09 (t, *J* = 1.9 Hz, 1H), 8.63 (d, *J* = 8.0 Hz, 1H), 8.30 (d, *J* = 8.0 Hz, 1H), 8.26 (dd, *J* = 8.0, 1.5 Hz, 1H), 7.76 (ddd, *J* = 8.6, 7.1, 1.7 Hz, 1H), 7.72 (t, *J* = 8.1 Hz, 1H), 7.65 (d, *J* = 8.5 Hz, 1H), 7.45 (ddd, *J* = 8.0, 7.1, 0.9 Hz, 1H), 7.33 (s, 1H). **^{13}C NMR** (75 MHz, $CDCl_3$) δ 173.69 , 155.57 , 148.70 , 142.07 , 139.38 , 134.48 , 133.50 , 132.96 , 129.85 , 125.77 , 125.13 , 124.53 , 122.51 , 120.67 , 118.51 . **HRMS** $C_{15}H_9NO_5$ requires 283.0481, found 283.0486 $[M+H]^+$.

3-hydroxy-2-[3-(trifluoromethyl)phenyl]chromen-4-one (4-C2)

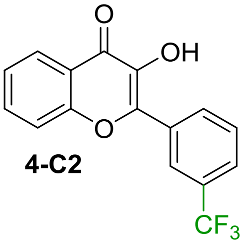

4-C2

Prepared according to general procedure A: white solid (49%). **^{1}H NMR** (500 MHz, $CDCl_3$) δ 8.51 (s, 1H), 8.47 (d, *J* = 7.9 Hz, 1H), 8.25 (d, *J* = 8.0 Hz, 1H), 7.77 – 7.69 (m, 2H), 7.66 (t, *J* = 7.9 Hz, 1H), 7.62 (d, *J* = 8.5 Hz, 1H), 7.43 (t, *J* = 7.5 Hz, 1H), 7.22 (s, 1H). **^{13}C NMR** (75 MHz, $CDCl_3$) δ 173.67 , 155.56 ,

143.12 , 139.06 , 134.19 , 132.07 , 131.31 (q, *J* = 32.6 Hz), 131.01 , 129.29 , 126.65 (q, *J* = 3.6 Hz), 125.71 , 124.93 , 124.49 (q, *J* = 3.9 Hz), 124.08 (q, *J* = 272.5 Hz), 120.70 , 118.47 . **HRMS** $C_{16}H_9F_3O_3$ requires 306.0504, found 306.0509 $[M+H]^+$.

2-(3-fluorophenyl)-3-hydroxy-chromen-4-one (4-C3)

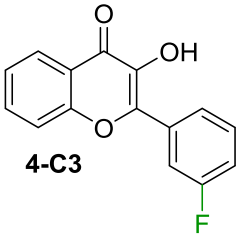

4-C3

Prepared according to general procedure A: white solid (61%). **^{1}H NMR** (500 MHz, $CDCl_3$) δ 8.25 (dd, *J* = 8.0, 1.3 Hz, 1H), 8.08 (d, *J* = 8.0 Hz, 1H), 7.99 (dt, *J* = 10.6, 2.0 Hz, 1H), 7.73 (td, *J* = 7.9, 7.2, 1.5 Hz, 1H), 7.59 (d, *J* = 8.5 Hz, 1H), 7.50 (td, *J* = 8.1, 6.1 Hz, 1H), 7.43 (t, *J* = 7.5 Hz, 1H), 7.20 – 7.13 (m, 2H). **^{13}C NMR** (75 MHz, $CDCl_3$) δ 173.94 , 163.21 (d, *J* = 245.5 Hz), 155.79 , 143.71 , 139.20 , 134.33 , 133.52 (d, *J* = 8.5 Hz), 130.56 (d, *J* = 8.2 Hz), 125.95 , 125.09 , 123.83 (d, *J* = 3.1 Hz), 120.97 , 118.69 , 117.45 (d, *J* = 21.3 Hz), 115.05 (d, *J* = 24.5 Hz). **HRMS** $C_{15}H_9FO_3$ requires 256.0536, found 256.0535 $[M+H]^+$.

2-(3-chlorophenyl)-3-hydroxy-chromen-4-one (4-C4)

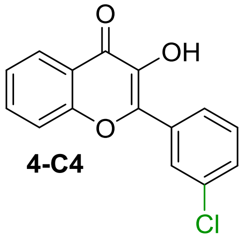

4-C4

Prepared according to general procedure A: white solid (69%). **^{1}H NMR** (500 MHz, $CDCl_3$) δ 8.26 – 8.22 (m, 2H), 8.16 (dt, *J* = 7.6, 1.6 Hz, 1H), 7.71 (ddd, *J* = 8.6, 7.1, 1.6 Hz, 1H), 7.58 (d, *J* = 8.5 Hz, 1H), 7.47 – 7.39 (m, 3H), 7.22 (s, 1H). **^{13}C NMR** (75 MHz, $CDCl_3$) δ 173.62 , 155.50 , 143.32 , 138.96 , 134.86 , 134.04 , 132.93 , 130.17 , 129.96 , 127.62 , 126.01 , 125.66 , 124.81 , 120.70 , 118.40 . **HRMS** $C_{15}H_9ClO_3$ requires 272.0240, found 272.0249 $[M+H]^+$.

2-(3-bromophenyl)-3-hydroxy-chromen-4-one (4-C5)

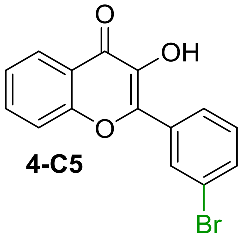

4-C5

Prepared according to general procedure A: white solid (87%). **^{1}H NMR** (500 MHz, $CDCl_3$) δ 8.38 (t, *J* = 1.6 Hz, 1H), 8.26 – 8.20 (m, 2H), 7.72 (ddd, *J* = 8.6, 7.2, 1.6 Hz, 1H), 7.62 – 7.55 (m, 2H), 7.45 – 7.37 (m, 2H), 7.19 (s, 1H). **^{13}C NMR** (75 MHz, $CDCl_3$) δ 173.62 , 155.52 , 143.20 , 138.95 , 134.06 , 133.17 , 133.10 , 130.46 , 130.21 , 126.49 , 125.67 , 124.83 , 122.92 , 120.70 , 118.43 . **HRMS** $C_{15}H_9BrO_3$ requires 315.9735, found 315.9737 $[M+H]^+$.

3-hydroxy-2-(3-iodophenyl)chromen-4-one (4-C6)

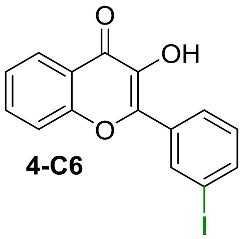

4-C6

Prepared according to general procedure A: white solid (50%). **^{1}H NMR** (500 MHz, $CDCl_3$) δ 8.56 (t, *J* = 1.6 Hz, 1H), 8.33 – 8.19 (m, 2H), 7.79 (d, *J* = 7.4 Hz, 1H), 7.72 (ddd, *J* = 8.6, 7.1, 1.6 Hz, 1H), 7.60 (d, *J* = 8.5 Hz, 1H), 7.43 (ddd, *J* = 8.0, 7.1, 0.9 Hz, 1H), 7.26 (t, *J* = 7.9 Hz, 1H), 7.13 (s, 1H). **^{13}C NMR** (75 MHz, $CDCl_3$) δ 173.58 , 155.54 , 143.08 , 139.04 , 138.89 , 136.28 , 134.06 , 133.19 , 130.32 , 127.11 , 125.67 , 124.84 , 120.70 , 118.46 , 94.44 , 77.37 . **HRMS** $C_{15}H_9IO_3$ requires 363.9596, found 363.9599 $[M+H]^+$.

3-hydroxy-2-(m-tolyl)chromen-4-one (4-C7)

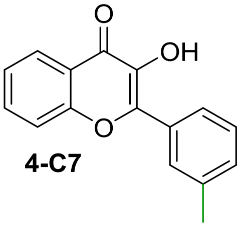

4-C7

Prepared according to general procedure A: white solid (76%). **^{1}H NMR** (500 MHz, $CDCl_3$) δ 8.26 (dd, *J* = 8.0, 1.4 Hz, 1H), 8.11 – 8.01 (m, 2H), 7.70 (td, *J* = 7.8, 7.1, 1.6 Hz, 1H), 7.60 (d, *J* = 8.5 Hz, 1H), 7.42 (q, *J* = 7.2 Hz, 2H), 7.29 (d, *J* = 7.5 Hz, 1H), 7.06 (s, 1H), 2.47 (s, 3H). **^{13}C NMR** (75 MHz, $CDCl_3$) δ 173.58 , 155.56 , 145.29 , 138.56 , 138.39 , 133.68 , 131.17 , 131.12 , 128.63 , 128.33 , 125.58 , 125.15 , 124.59 , 120.79 , 118.42 , 21.77 . **HRMS** $C_{16}H_{12}O_3$ requires 252.0786, found 252.0787 $[M+H]^+$.

3-hydroxy-2-(3-methoxyphenyl)chromen-4-one (4-C8)

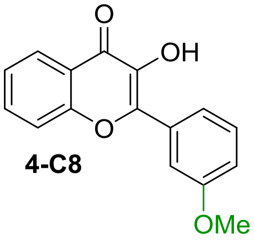

4-C8

Prepared according to general procedure A: white solid (56%). **^{1}H NMR** (500 MHz, $CDCl_3$) δ 8.26 (dd, *J* = 8.0, 1.4 Hz, 1H), 7.86 (d, *J* = 7.9 Hz, 1H), 7.84 – 7.82 (m, 1H), 7.71 (ddd, *J* = 8.6, 7.1, 1.6 Hz, 1H), 7.60 (d, *J* = 8.5 Hz, 1H), 7.48 – 7.40 (m, 2H), 7.05 (s, 1H), 7.03 (dd, *J* = 8.3, 1.9 Hz, 1H), 3.91 (s, 3H). **^{13}C NMR** (75 MHz, $CDCl_3$) δ 173.63 , 159.84 , 155.58 , 144.78 , 138.70 , 133.82 , 132.48 , 129.78 , 125.63 , 124.67 , 120.77 , 120.41 , 118.46 , 116.14 , 113.40 , 55.57 . **HRMS** $C_{16}H_{12}O_4$ requires 268.0736, found 268.0740 $[M+H]^+$.

2-(3-aminophenyl)-3-hydroxy-chromen-4-one (4-C9)

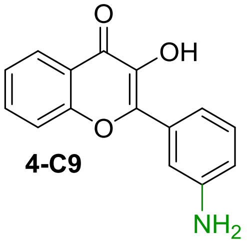

4-C9

Prepared from **4-C1** according to general procedure B: yellow solid (98%). **^{1}H NMR** (500 MHz, DMSO-d_6) δ 9.37 (s, 1H), 8.11 (d, *J* = 7.6 Hz, 1H), 7.79 (t, *J* = 7.5 Hz, 1H), 7.69 (d, *J* = 8.2 Hz, 1H), 7.51 – 7.40 (m, 1H), 7.37 (d, *J* = 7.3 Hz, 1H), 7.19 (t, *J* = 7.7 Hz, 1H), 6.70 (d, *J* = 7.3 Hz, 1H), 5.31 (s, 2H). **^{13}C**

NMR (75 MHz, DMSO-d_6) δ 172.87 , 154.51 , 148.69 , 146.19 , 138.79 , 133.63 , 131.74 , 128.89 , 124.79 , 124.46 , 121.25 , 118.23 , 115.63 , 115.54 , 112.85 . **HRMS** $C_{15}H_{11}NO_3$ requires 253.0739, found 253.0731 $[M+H]^+$.

3-hydroxy-2-(3-hydroxyphenyl)chromen-4-one (4-C10)

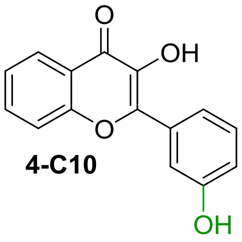

4-C10

Prepared from **4-C8** according to general procedure C: pink solid (43%). ^{1}**H NMR** (500 MHz, DMSO-d_6) δ 9.71 (s, 1H), 9.56 (s, 1H), 8.12 (dd, *J* = 8.0, 1.4 Hz, 1H), 7.81 (ddd, *J* = 8.4, 7.3, 1.4 Hz, 1H), 7.74 (d, *J* = 8.4 Hz, 1H), 7.69 (s, 1H), 7.66 (d, *J* = 8.0 Hz, 1H), 7.47 (t, *J* = 7.5 Hz, 1H), 7.36 (t, *J* = 8.0 Hz, 1H), 6.90 (d, *J* = 7.9 Hz, 1H). ^{13}**C NMR** (75 MHz, DMSO-d_6) δ 172.93 , 157.26 , 154.50 , 145.20 , 139.00 , 133.70 , 132.37 , 129.48 , 124.76 , 124.50 , 121.22 , 118.41 , 118.31 , 117.01 , 114.53 . **HRMS** $C_{15}H_{10}O_4$ requires 254.0579, found 254.0577 $[M+H]^+$.

3-hydroxy-2-(4-nitrophenyl)chromen-4-one (4-D1)

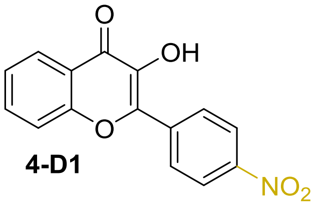

4-D1

Prepared according to general procedure A: yellow solid (56%). ^{1}**H NMR** (500 MHz, $CDCl_3$) δ 8.47 (d, *J* = 9.1 Hz, 2H), 8.38 (d, *J* = 9.1 Hz, 2H), 8.28 (dd, *J* = 8.0, 1.3 Hz, 1H), 7.78 (ddd, *J* = 8.6, 7.4, 1.5 Hz, 1H), 7.64 (d, *J* = 8.5 Hz, 1H), 7.47 (t, *J* = 7.5 Hz, 1H), 7.29 (s, 1H). ^{13}**C NMR** (126 MHz, $CDCl_3$) δ 173.75 , 155.70 , 148.20 , 141.99 , 139.88 , 137.28 , 134.61 , 128.56 , 125.85 , 125.17 , 123.91 , 120.60 , 118.52 . **HRMS** $C_{15}H_9NO_5$ requires 283.0481, found 283.0480 $[M+H]^+$.

3-hydroxy-2-[4-(trifluoromethyl)phenyl]chromen-4-one (4-D2)

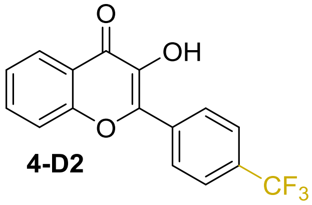

4-D2

Prepared according to general procedure A: white solid (67%). ^{1}**H NMR** (500 MHz, $CDCl_3$) δ 8.38 (d, *J* = 8.3 Hz, 2H), 8.31 – 8.20 (m, 1H), 7.78 (d, *J* = 8.4 Hz, 2H), 7.74 (ddd, *J* = 8.4, 7.1, 1.4 Hz, 1H), 7.60 (d, *J* = 8.5 Hz, 1H), 7.44 (t, *J* = 7.5 Hz, 1H), 7.23 (s, 1H). ^{13}**C NMR** (75 MHz, $CDCl_3$) δ 173.72 , 155.61 , 143.11 , 139.32 , 134.62 , 134.24 , 131.65 (q, *J* = 32.7 Hz), 128.06 , 125.74 , 125.62 (q, *J* = 3.7 Hz), 124.94 , 123.98 (q, *J* = 272.3 Hz), 120.69 , 118.45 . **HRMS** $C_{16}H_9F_3O_3$ requires 306.0504, found 306.0500 $[M+H]^+$.

2-(4-fluorophenyl)-3-hydroxy-chromen-4-one (4-D3)

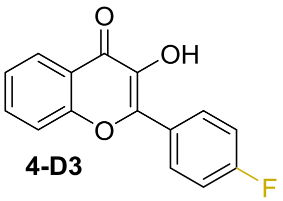

4-D3

Prepared according to general procedure A: white solid (61%). **^{1}H NMR** (500 MHz, $CDCl_3$) δ 8.34 – 8.18 (m, 3H), 7.70 (td, *J* = 7.9, 7.2, 1.5 Hz, 1H), 7.57 (d, *J* = 8.5 Hz, 1H), 7.47 – 7.37 (m, 1H), 7.21 (t, *J* = 8.7 Hz, 2H), 7.12 (s, 1H). **^{13}C NMR** (75 MHz, $CDCl_3$) δ 173.54 , 163.74 (d, *J* = 252.0 Hz), 155.48 , 144.23 , 138.30 , 133.82 , 130.11 (d, *J* = 8.5 Hz), 127.40 (d, *J* = 3.3 Hz), 125.65 , 124.73 , 120.79 , 118.33 , 115.90 (d, *J* = 21.7 Hz). **HRMS** $C_{15}H_9FO_3$ requires 256.0536, found 256.0538 $[M+H]^+$.

2-(4-chlorophenyl)-3-hydroxy-chromen-4-one (4-D4)

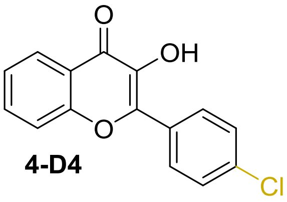

4-D4

Prepared according to general procedure A: white solid (76%). **^{1}H NMR** (500 MHz, $CDCl_3$) δ 8.31 – 8.14 (m, 3H), 7.72 (t, *J* = 7.2 Hz, 1H), 7.58 (d, *J* = 8.4 Hz, 1H), 7.50 (d, *J* = 8.5 Hz, 2H), 7.43 (t, *J* = 7.3 Hz, 1H), 7.10 (s, 1H). **^{13}C NMR** (75 MHz, $CDCl_3$) δ 173.56 , 155.52 , 143.91 , 138.65 , 136.36 , 133.96 , 129.70 , 129.16 , 129.04 , 125.67 , 124.80 , 120.76 , 118.39 . **HRMS** $C_{15}H_9ClO_3$ requires 272.0240, found 272.0243 $[M+H]^+$.

2-(4-bromophenyl)-3-hydroxy-chromen-4-one (4-D5)

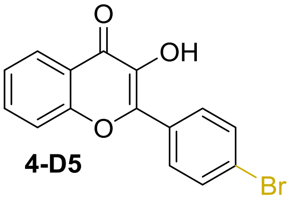

4-D5

Prepared according to general procedure A : white solid (86%). **^{1}H NMR** (500 MHz, $CDCl_3$) δ 8.25 (dd, *J* = 8.0, 1.3 Hz, 1H), 8.14 (d, *J* = 8.7 Hz, 2H), 7.72 (td, *J* = 7.9, 7.2, 1.5 Hz, 1H), 7.66 (d, *J* = 8.7 Hz, 2H), 7.58 (d, *J* = 8.5 Hz, 1H), 7.46 – 7.39 (m, 1H), 7.13 (s, 1H). **^{13}C NMR** (75 MHz, $CDCl_3$) δ 173.58 , 155.53 , 143.96 , 138.72 , 133.99 , 132.02 , 130.16 , 129.34 , 125.68 , 124.82 , 120.77(2xC) , 118.40 . **HRMS** $C_{15}H_9BrO_3$ requires 315.9735, found 315.9742 $[M+H]^+$.

3-hydroxy-2-(4-iodophenyl)chromen-4-one (4-D6)

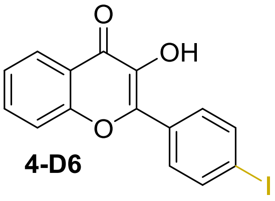

4-D6

Prepared according to general procedure A: white solid (66%). **^{1}H NMR** (500 MHz, $CDCl_3$) δ 8.25 (dd, *J* = 8.0, 1.5 Hz, 1H), 8.00 (d, *J* = 8.7 Hz, 2H), 7.88 (d, *J* = 8.7 Hz, 2H), 7.72 (ddd, *J* = 8.7, 7.1, 1.7 Hz, 1H), 7.59 (d, *J* = 8.4 Hz, 1H), 7.43 (td, *J* = 7.6, 7.1, 0.9 Hz, 1H), 7.06 (s, 1H). **^{13}C NMR** (75 MHz, $CDCl_3$) δ 173.56 , 155.55 , 144.00 , 138.81 , 138.02 , 134.00 , 130.75 , 129.33 , 125.69 , 124.82 , 120.76 , 118.42 , 96.97 . **HRMS** $C_{15}H_9IO_3$ requires 363.9596, found 363.9594 $[M+H]^+$.

3-hydroxy-2-(p-tolyl)chromen-4-one (4-D7)

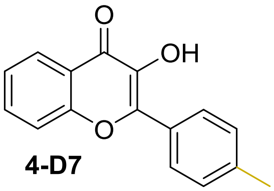

4-D7

Prepared according to general procedure A: white solid (80%). **^{1}H NMR** (500 MHz, $CDCl_3$) δ 8.25 (dd, *J* = 8.0, 1.5 Hz, 1H), 8.16 (d, *J* = 8.3 Hz, 2H), 7.69 (ddd, *J* = 8.6, 7.1, 1.6 Hz, 1H), 7.58 (d, *J* = 8.4 Hz, 1H), 7.41 (ddd, *J* = 8.0, 7.2, 0.9 Hz, 1H), 7.34 (d, *J* = 8.1 Hz, 2H), 7.03 (s, 1H), 2.44 (s, 3H). **^{13}C NMR** (75 MHz, $CDCl_3$) δ 173.47 , 155.49 , 145.43 , 140.74 , 138.28 , 133.59 , 129.47 , 128.39 , 127.84 , 125.55 , 124.56 , 120.83 , 118.38 , 21.69 . **HRMS** $C_{16}H_{12}O_3$ requires 252.0786, found 252.0789 $[M+H]^+$.

3-hydroxy-2-(4-methoxyphenyl)chromen-4-one (4-D8)

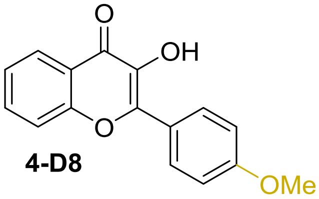

4-D8

Prepared according to general procedure A: white solid (64%). **^{1}H NMR** (500 MHz, $CDCl_3$) δ 8.28 – 8.22 (m, 3H), 7.70 (t, *J* = 7.7 Hz, 1H), 7.59 (d, *J* = 8.4 Hz, 1H), 7.41 (t, *J* = 7.5 Hz, 1H), 7.06 (d, *J* = 8.9 Hz, 2H), 6.95 (s, 1H), 3.90 (s, 3H). **^{13}C NMR** (126 MHz, $CDCl_3$) δ 173.29 , 161.26 , 155.45 , 145.46 , 133.51 , 129.68 , 125.57 , 124.58 , 123.71 , 120.89 , 118.33 , 114.26 , 55.58 . **HRMS** $C_{16}H_{12}O_4$ requires 268.0736, found 268.0739 $[M+H]^+$.

3-hydroxy-2-(4-hydroxyphenyl)chromen-4-one (4-D10)

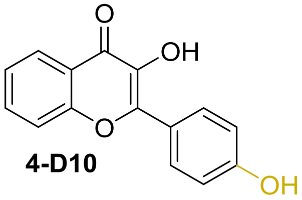

4-D10

Prepared from **4-D8** according to general procedure C: pink solid (34%). **^{1}H NMR** (500 MHz, DMSO-*d*$_6$) δ 10.09 (s, 1H), 9.33 (s, 1H), 8.16 – 8.07 (m, 3H), 7.77 (ddd, *J* = 8.5, 6.9, 1.7 Hz, 1H), 7.72 (dd, *J* = 8.6, 1.0 Hz, 1H), 7.45 (ddd, *J* = 8.0, 6.9, 1.1 Hz, 1H), 6.95 (d, *J* = 8.9 Hz, 2H). **^{13}C NMR** (75 MHz, DMSO-*d*$_6$) δ 172.48 , 159.11 , 154.37 , 146.08 , 137.79 , 133.35 , 129.56 , 124.69 , 124.39 , 121.97 , 121.34 , 118.24 , 115.43 . **HRMS** $C_{15}H_{10}O_4$ requires 254.0579, found 254.0575 $[M+H]^+$.

2-(2-furyl)-3-hydroxy-chromen-4-one (4-E1)

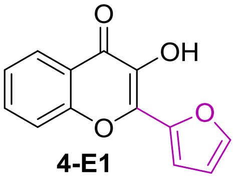

4-E1

Prepared from according to general procedure A (from furfural instead of a benzaldehyde): white solid (43%). **^{1}H NMR** (300 MHz, $CDCl_3$) δ 8.18 (dd, *J* = 7.5, 2.0 Hz, 1H), 7.87 (td, *J* = 7.5, 2.0 Hz, 1H), 7.66 (dd, *J* = 7.4, 2.1 Hz, 1H), 7.55 (td, *J* = 7.4, 1.9 Hz, 1H), 7.34 (dd, *J* = 7.5, 1.3 Hz, 1H), 7.03 (s, 1H), 6.74 (dd, *J* = 7.5, 1.5 Hz, 1H), 6.47 (t, *J* = 7.5 Hz, 1H). **^{13}C NMR** (75 MHz, $CDCl_3$) δ 172.56 , 155.21 , 144.91 ,

144.56 , 138.90 , 136.54 , 133.69 , 125.56 , 124.80 , 121.34 , 118.48 , 115.84 , 112.82 . **HRMS** $C_{13}H_8O_4$ requires 228.0422, found 228.0430 $[M+H]^+$.

3-hydroxy-2-(2-thienyl)chromen-4-one (4-E2)

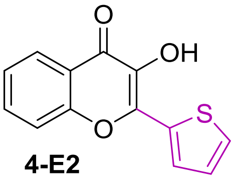

4-E2

Prepared according to general procedure A (from thiophene-2-carbaldehyde instead of a benzaldehyde): white solid (54%). **^{1}H NMR** (300 MHz, $CDCl_3$) δ 8.19 (dd, *J* = 7.5, 1.9 Hz, 1H), 7.87 (td, *J* = 7.5, 1.9 Hz, 1H), 7.67 (dd, *J* = 7.5, 2.0 Hz, 1H), 7.62 – 7.50 (m, 2H), 7.36 – 7.20 (m, 2H), 7.03 (s, 1H). **^{13}C NMR** (75 MHz, $CDCl_3$) δ 172.68 , 155.22 , 142.78 , 136.45 , 133.66 , 133.07 , 129.98 , 129.60 , 128.25 , 125.58 , 124.72 , 121.20 , 118.28 . **HRMS** $C_{13}H_8O_3S$ requires 244.0194, found 244.0191 $[M+H]^+$.

Synthesis of AM12

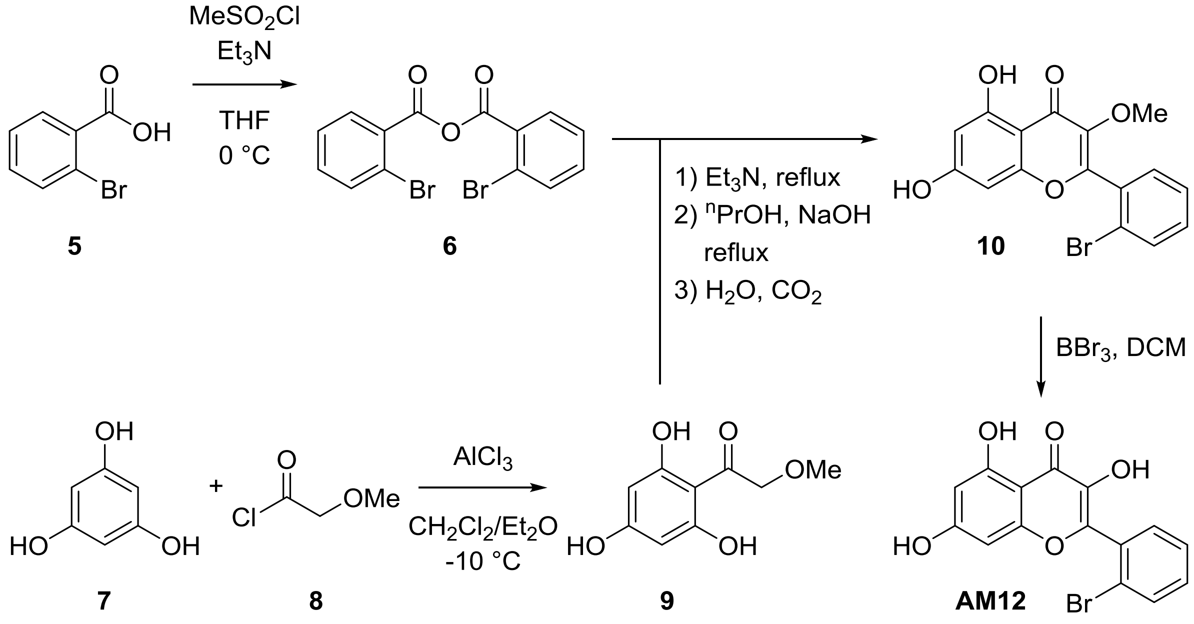

Scheme S2. Synthesis of synthetic flavonol **AM12**. Benzoic anhydride **6** was prepared from its corresponding benzoic acid **5**. Friedel-Crafts acylation of phloroglucinol with acyl chloride **8** gave intermediate **9**. Combination of building blocks **6** and **9** in an Allan-Robinson reaction followed by boron tribromide-mediated demethylation afforded **AM12**. THF: tetrahydrofuran.

2-bromobenzoic anhydride (6)

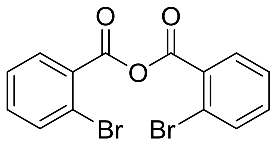

6

A solution 2-bromobenzoic acid **5** (8.207 g, 40.8 mmol) and methanesulfonyl chloride (1.58 ml, 20.4 mmol) in THF (100 ml) was stirred at 0 ^{o}C. A mixture of triethylamine (16 ml) and THF (14 ml) was added dropwise and the resulting mixture was warmed to room temperature while stirring over 2 hours. Then, the reaction mixture was concentrated in vacuo, and the residue suspended in 10% $NaHCO_3$ (40 ml). The suspension extracted with EtOAc (3 × 30 ml). The organic layers were combined, dried with Na_2SO_4 and concentrated in vacuo to give the title compound as a white, crystalline product (7.41 g, 95%). **Rf** = 0.29 (EtOAc:hexanes 2:8). m.p. 70-71 °C. **^{1}H NMR** (300 MHz, $CDCl_3$) δ 8.07-7.93 (m, 2H), 7.78-7.70 (m, 2H), 7.48-7.40 (m, 4H). **^{13}C NMR** (125 Mhz, $CDCl_3$) δ 201.5, 166.9, 137.8, 129.0, 128.2, 125.3, 89.9. ν_{max}/cm^{-1} (solid state) = 3064, 2982,1784 and 1720. **HRMS**: $C_{14}H_8Br_2O_3$ requires 406.8712, found 406.8727 $[M+Na]^+$.

2-methoxy-1-(2,4,6-trihydroxyphenyl)ethanone (9)

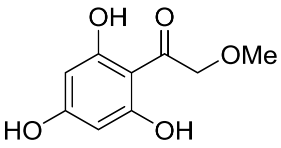

9

$AlCl_3$ (11 g, 82 mmol) in DCM (40 ml) was added to a dry, nitrogen-flushed flask. The solution was cooled to −10 ^{o}C, while stirring, in 20 min, followed by addition of Et_2O (75 ml). After further stirring at −10 ^{o}C for 10 min, phloroglucinol **7** (2.52 g, 20 mmol) was added. Then, a solution of methoxyacetal chloride **8** (2.0 ml, 20 mmol) in Et_2O (10 ml) was added dropwise. The mixture was stirred overnight at 0 ^{o}C, after which it was poured onto ice (70 g). Once the ice had melted, the mixture was stirred vigourously in Rochelle's salt (50%, 100 ml) for 30–60 mins and the layers were seperated. The aqueous layer was extracted with Et_2O (4 × 60 ml) and the organic layers were combined, washed with water (100 ml), dried with Na_2SO_4, and concentrated in vacuo to give an off-white solid (0.919 g, 23%). **Rf** = 0.23 (EtOAc:hexane 75:25). m.p. 154-155 °C. **^{1}H NMR** (300 MHz, DMSO-d_6) δ 12.18 (s, 2H), 10.43 (s, 1H), 8.95 (s, 1H), 5.81 (s, 1H), 5.65 (s, 1H), 4.58 (s, 2H), 3.34 (s, 3H). **^{13}C NMR** (125 MHz, DMSO-d_6) δ 201.4 , 164.9 , 164.0 , 158.9 , 102.6 , 94.5 , 94.0 , 77.0 , 58.5 . ν_{max}/cm^{-1} (solid state) = 3280, 2920, 2852 and 1592. **HRMS** requires 419.0949, found 419.0948 $[2M+Na]^+$.

2-(2-bromophenyl)-5,7-dihydroxy-3-methoxychromen-4-one (10)

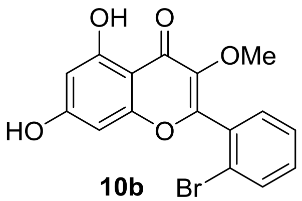

10b

2-Methoxy-1-(2,4,6-trihydroxyphenyl)ethanone **9** (400 mg, 2.02 mmol) and 2-bromobenzoic anhydride **6** (2.33 g, 6.06 mmol) were dissolved in triethylamine (5 ml) and heated to reflux for 5 hours. *Reagents did not dissolve readily but formed a homogeneous solution at 72 ^oC.* Then, the reaction mixture was allowed to cool to room temperature, and 1-propanol (4 ml) and NaOH (2M, 5 ml) were added. The reaction mixture was heated at reflux for 14 hours. After cooling to room temperature, the reaction mixture was diluted to 20 ml with water. The volatiles were evaporated in vacuo. Saturation of the aqueous mixture CO_2 (cardice) gave a yellow solid. Purification by flash column chromatography on SiO_2 (EtOAc:hexanes 25:75) gave the title compound as a pale yellow solid (492 mg, 68%). **Rf** = 0.36 (EtOAc:hexanes 25:75). m.p. 245-246 °C. **^{1}H NMR** (300MHz, DMSO-d_6) δ 12.47 (s, 1H), 11.00 (s, 1H), 7.83 (d, *J* = 1.5, 1H), 7.68 (d, *J* = 2.0, 1H), 7.61-7.47 (m, 2H), 6.38 (d, J = 2.1, 1H), 6.26 (d, *J* = 2.0, 1H), 3.68 (s, 3H). **^{13}C NMR** (125 Mhz, DMSO-d_6) δ 178.0 , 164.6, 161.4, 157.0, 156.6, 149.1, 132.8, 132.5, 131.5, 127.9, 122.1, 105.0, 98.9, 93.9, 60.3. ν_{max}/cm^{-1} (solid state) = 3228, 2961, 2922, 2850, 1709 and 1656. **HRMS** $C_{16}H_{11}O_5Br$ requires 384.9682, found 384.9782 $[M+Na]^+$.

2-(2-bromophenyl)-3,5,7-trihydroxychromen-4-one (AM12)

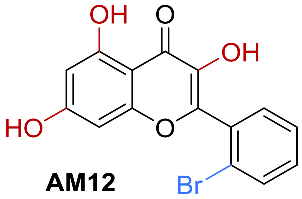

AM12

A solution of BBr_3 in DCM (1M, 0.63 mL) was added dropwise to a solution of 2-(2-bromophenyl)-5,7-dihydroxy-3-methoxychromen-4-one **10** (75 mg, 0.21 mmol) in DCM (10 mL) at 0 °C under inert atmosphere. The mixture was stirred for a further 1 hr at room temperature. The pH of the reaction mixture was confirmed at pH=1 before quenching with water (30 mL) at 0 °C. The reaction mixture was diluted with DCM (30 mL), and extracted with EtOAc (3 × 30 mL). The organic layers were washed with water (3 × 30 mL), dried (Na_2SO_4) and concentrated in vacuo to yield a pale yellow solid. The resulting solid was purified by column chromatography on SiO_2 (acetone:hexane 35:75) to yield the title compound as a pale yellow solid (25 mg, 18%). **Rf** = 0.30 (acetone:hexanes 35:65). m.p. 245-246 °C. **^{1}H NMR** (300 MHz, acetone-d_6) δ 12.18 (s, 1H) 7.83 (dd, *J* = 8.0, 1.3, 1H), 7.74 (dd, 7.6, 1.8, 1H), 7.59 (td, J = 7.5, 1.3, 1H), 7.51 (td, *J* = 7.5, 1.3, 1H), 6.44 (d, *J* = 2.1, 1H), 6.31 (d, *J* = 2.1, 1H). **^{13}C NMR** (125 MHz, acetone-d_6) δ 206.1, 187.1, 175.1, 168.3, 157.7, 148.1, 143.9, 142.8, 134.1, 133.0, 132.8, 128.5, 114.8, 109.2, 104.5.

ν_{max}/cm^{-1} (solid state) = 3228, 2961, 2922, 2850, 1709 and 1656. **HRMS** $C_{15}H_9O_5Br$ requires 348.9706, found 348.9693 $[M+H]^+$.

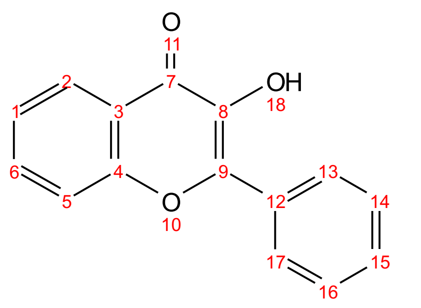

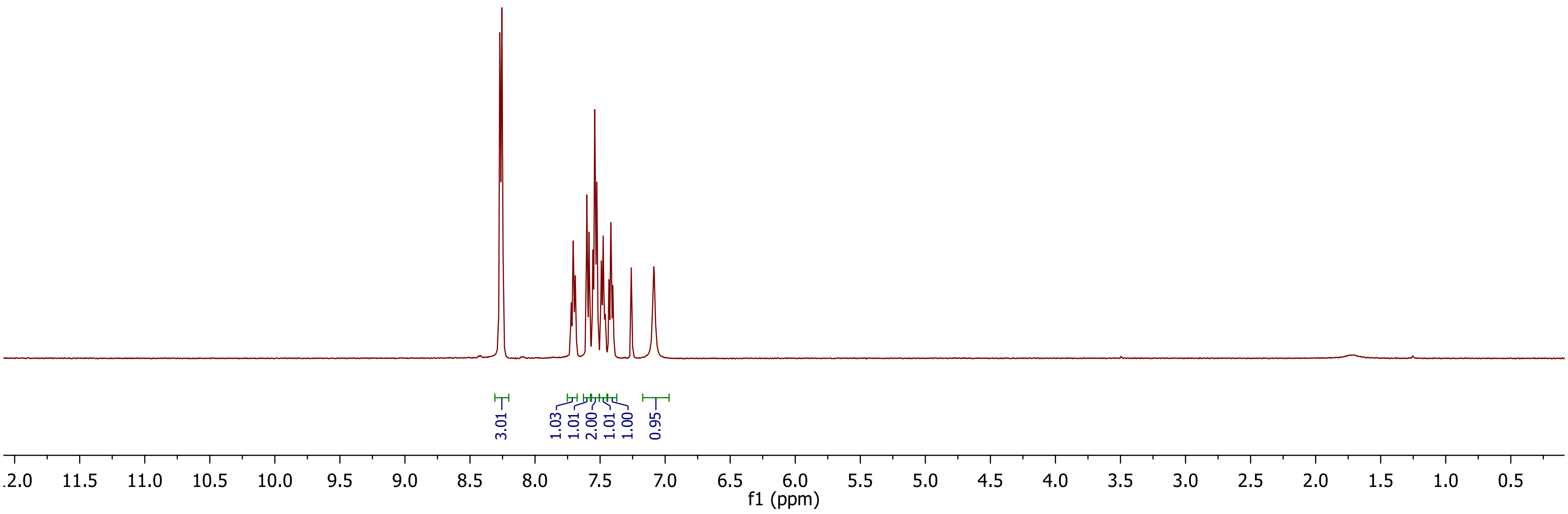
3.01
1.03
1.01
2.00
1.01
1.00
0.95
12.0
11.5
11.0
10.5
10.0
9.5
9.0
8.5
8.0
7.5
7.0
6.5
6.0
5.5
5.0
4.5
4.0
3.5
3.0
2.5
2.0
1.5
1.0
0.5
f1 (ppm)

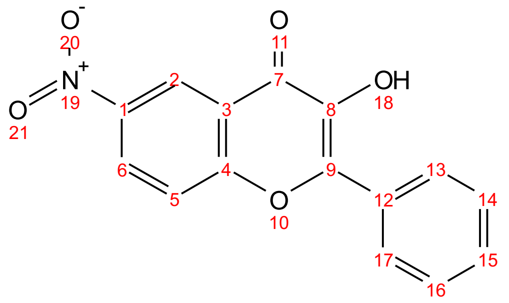

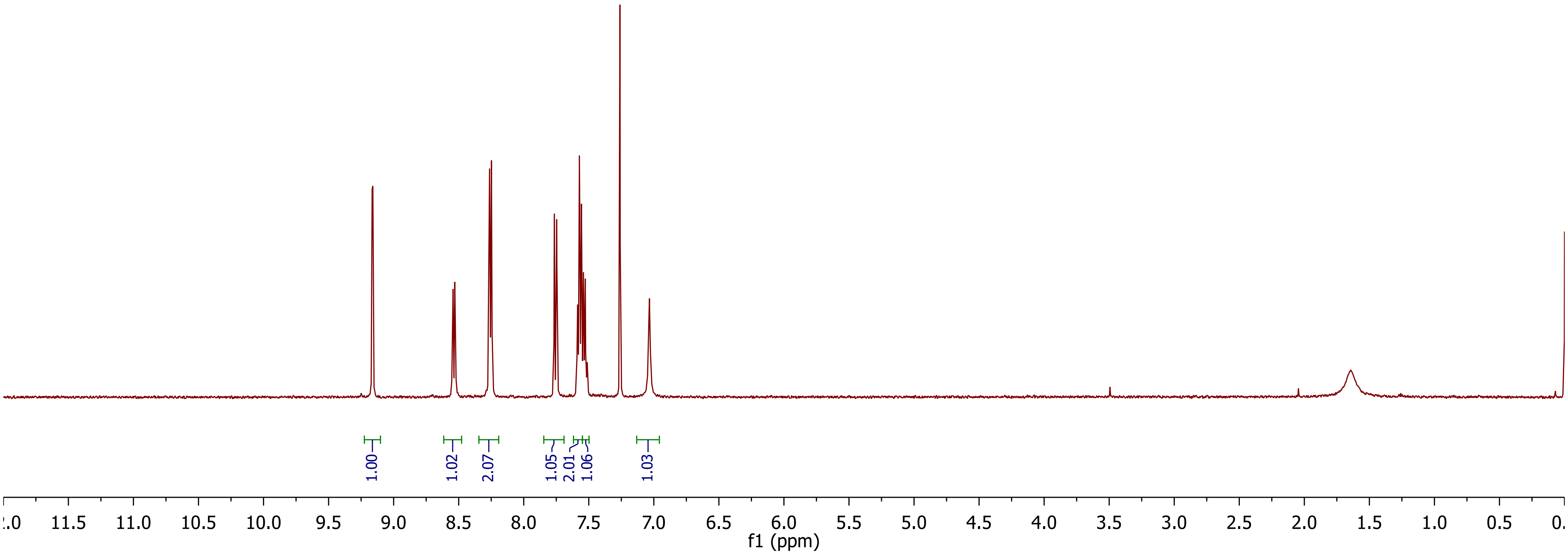
1.00
1.02
2.07
1.05
2.01
1.06
1.03
f1 (ppm)

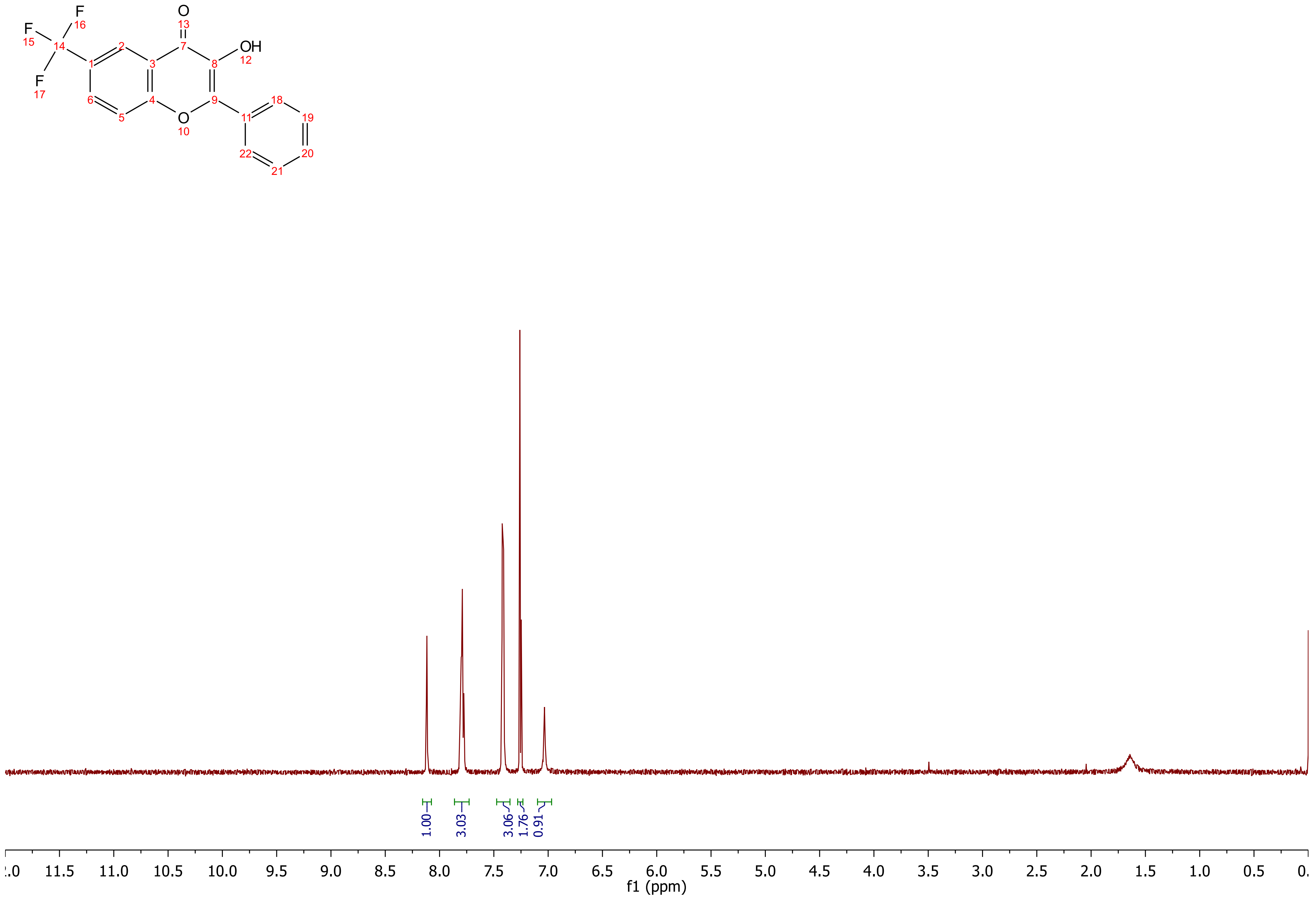

O
OH
F
F
F
O
1.00
3.03
3.06
1.76
0.91
12.0
11.5
11.0
10.5
10.0
9.5
9.0
8.5
8.0
7.5
7.0
6.5
6.0
5.5
5.0
4.5
4.0
3.5
3.0
2.5
2.0
1.5
1.0
0.5
0.
f1 (ppm)

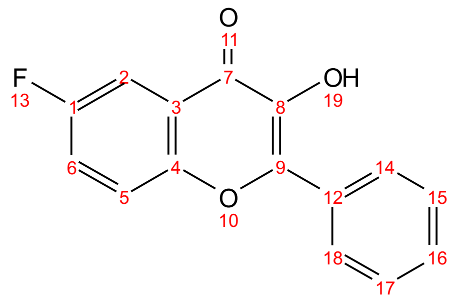

O
11
F
13
OH
19
O
10

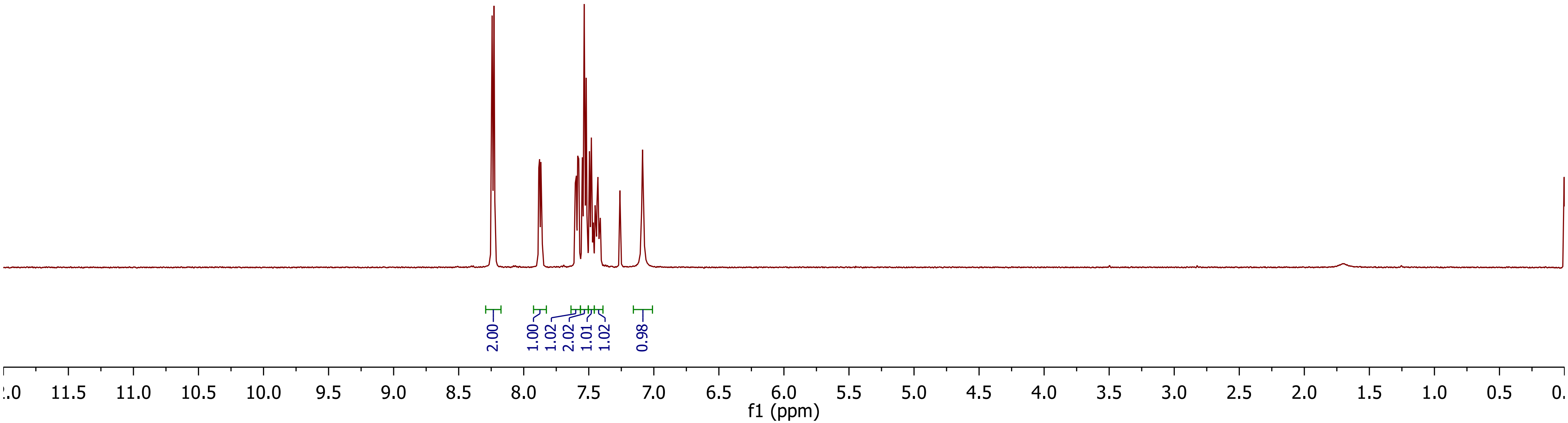

2.00
1.00
1.02
2.02
1.01
1.02
0.98
11.5
11.0
10.5
10.0
9.5
9.0
8.5
8.0
7.5
7.0
6.5
6.0
5.5
5.0
4.5
4.0
3.5
3.0
2.5
2.0
1.5
1.0
0.5
f1 (ppm)

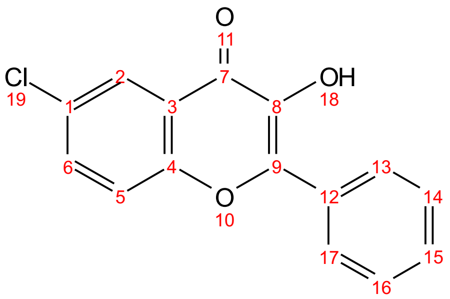
Cl
OH
O
O

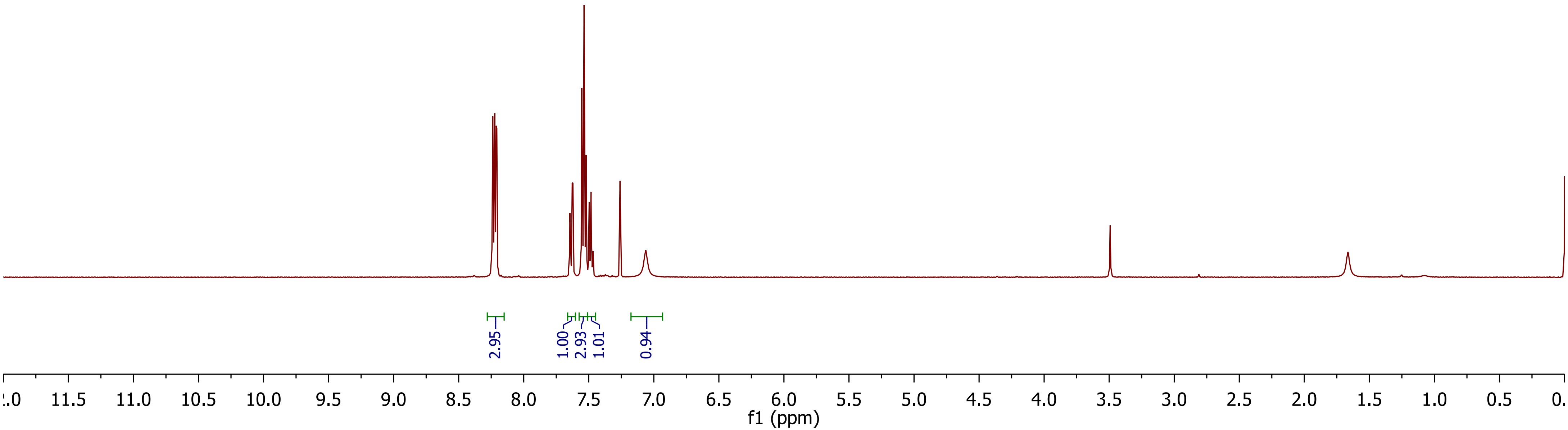
2.95
1.00
2.93
1.01
0.94
11.5
11.0
10.5
10.0
9.5
9.0
8.5
8.0
7.5
7.0
6.5
6.0
5.5
5.0
4.5
4.0
3.5
3.0
2.5
2.0
1.5
1.0
0.5
f1 (ppm)

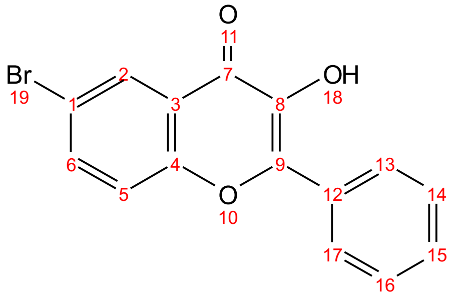

Br
O
OH
O

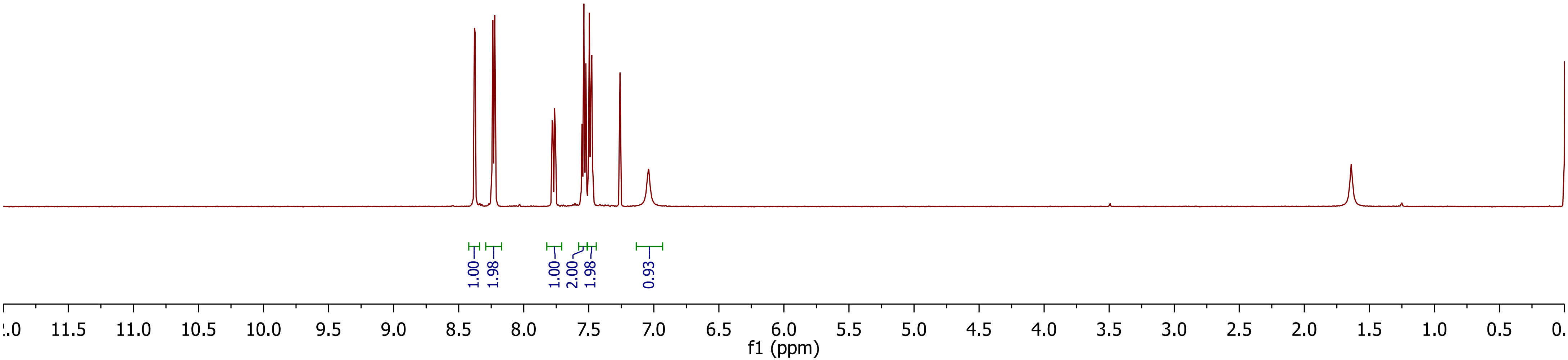

1.00
1.98
1.00
2.00
1.98
0.93
11.5 11.0 10.5 10.0 9.5 9.0 8.5 8.0 7.5 7.0 6.5 6.0 5.5 5.0 4.5 4.0 3.5 3.0 2.5 2.0 1.5 1.0 0.5
f1 (ppm)

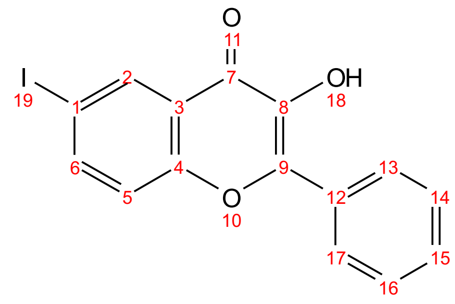

O
11
OH
18
I
19
2
7
8
1
3
13
6
4
9
14
12
5
O
10
17
15
16

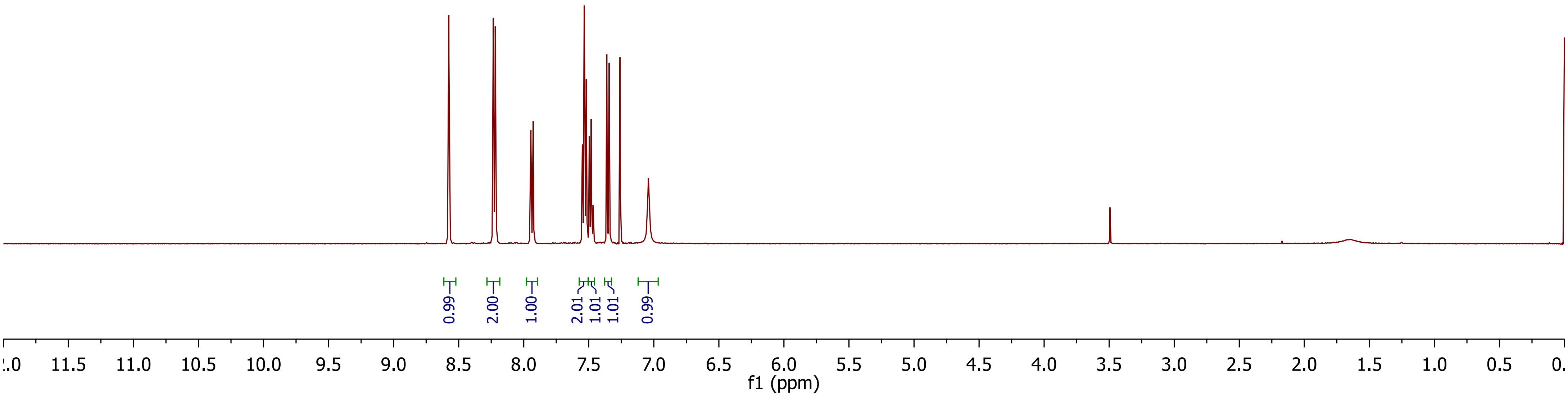

0.99
2.00
1.00
2.01
1.01
1.01
0.99
11.5
11.0
10.5
10.0
9.5
9.0
8.5
8.0
7.5
7.0
6.5
6.0
5.5
5.0
4.5
4.0
3.5
3.0
2.5
2.0
1.5
1.0
0.5
f1 (ppm)

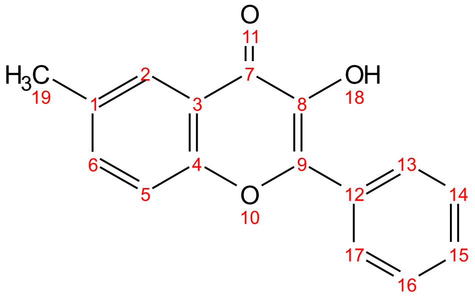

H_3C
O
OH
O

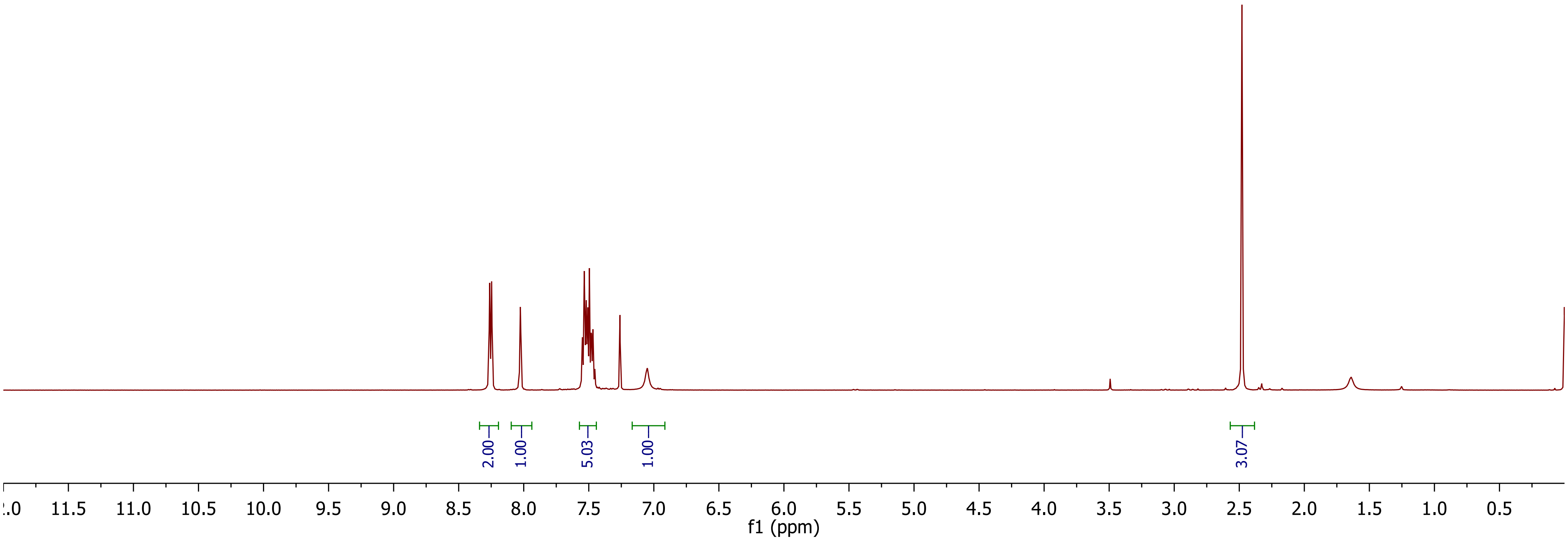

2.00
1.00
5.03
1.00
3.07
11.5
11.0
10.5
10.0
9.5
9.0
8.5
8.0
7.5
7.0
6.5
6.0
5.5
5.0
4.5
4.0
3.5
3.0
2.5
2.0
1.5
1.0
0.5
f1 (ppm)

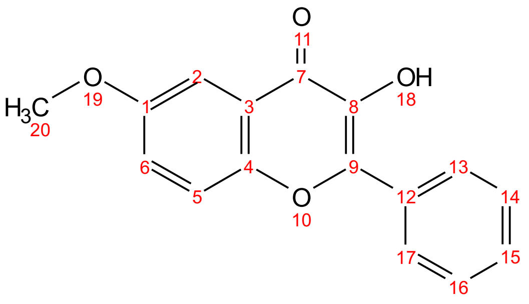
O
11
OH
18
H3C
20
O
19
1
2
3
7
8
9
4
5
6
O
10
12
13
14
15
16
17

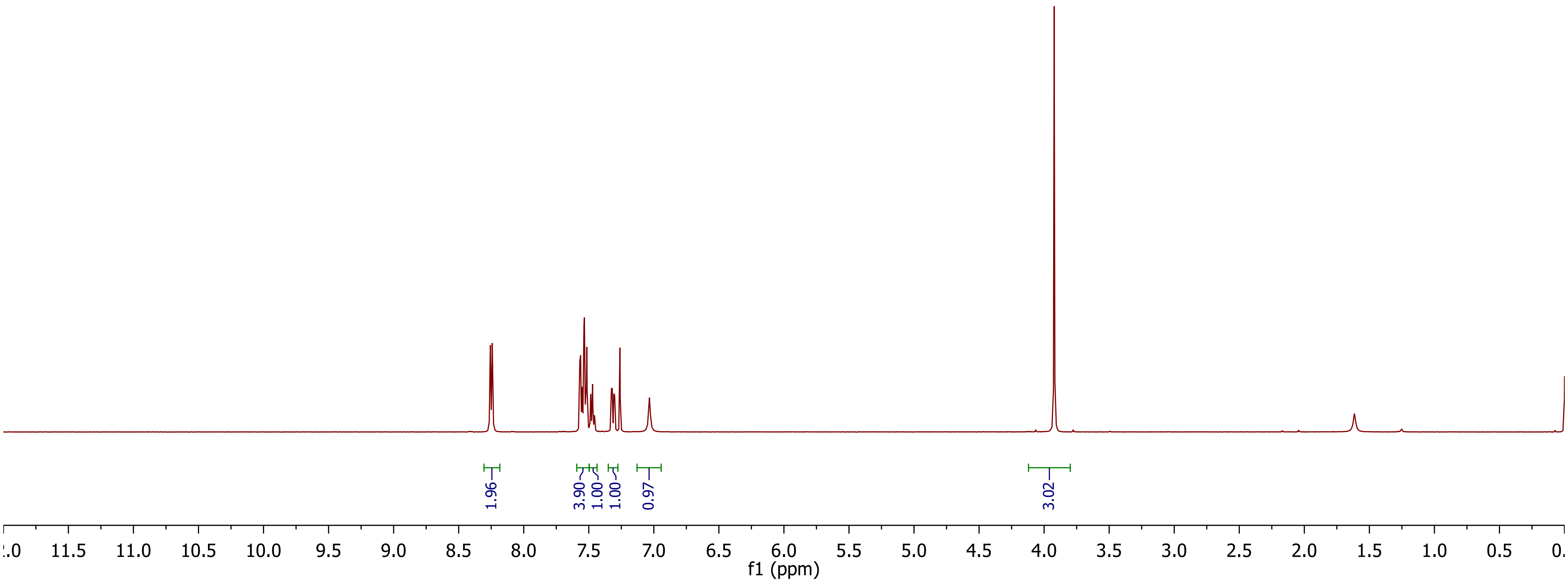
1.96
3.90
1.00
1.00
0.97
3.02
11.5
11.0
10.5
10.0
9.5
9.0
8.5
8.0
7.5
7.0
6.5
6.0
5.5
5.0
4.5
4.0
3.5
3.0
2.5
2.0
1.5
1.0
0.5
f1 (ppm)

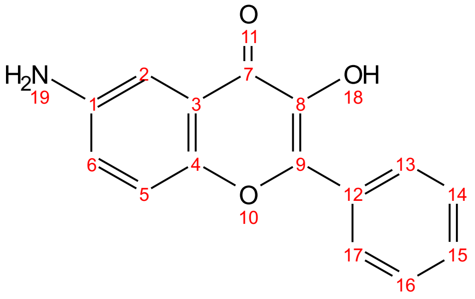

H2N
OH
O
O

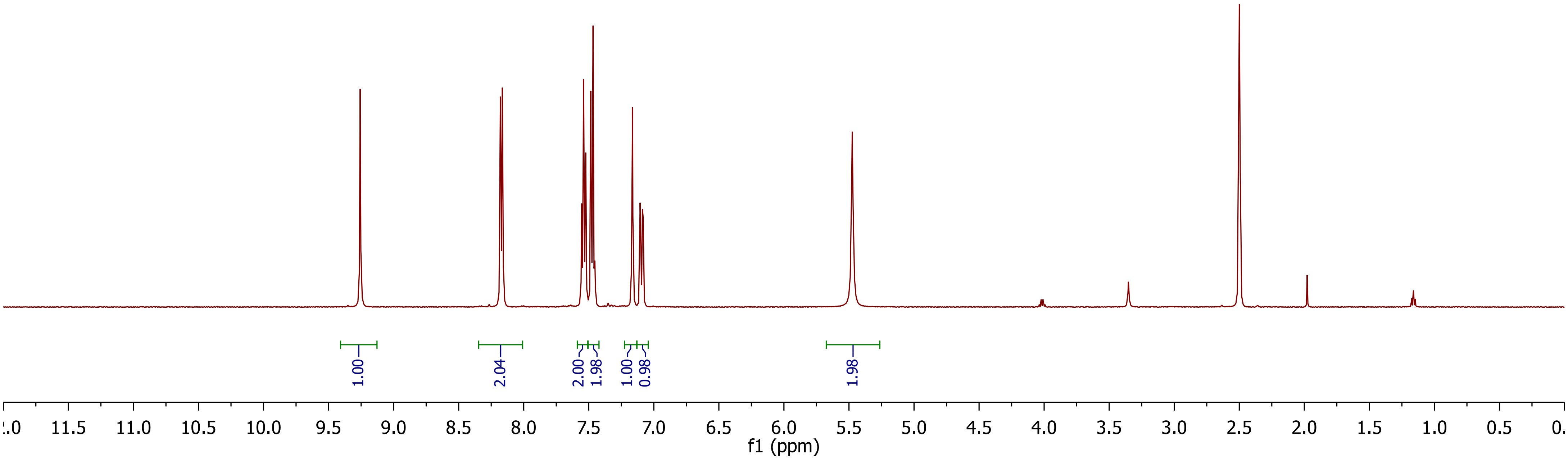

1.00
2.04
2.00
1.98
1.00
0.98
1.98
11.5
11.0
10.5
10.0
9.5
9.0
8.5
8.0
7.5
7.0
6.5
6.0
5.5
5.0
4.5
4.0
3.5
3.0
2.5
2.0
1.5
1.0
0.5
f1 (ppm)

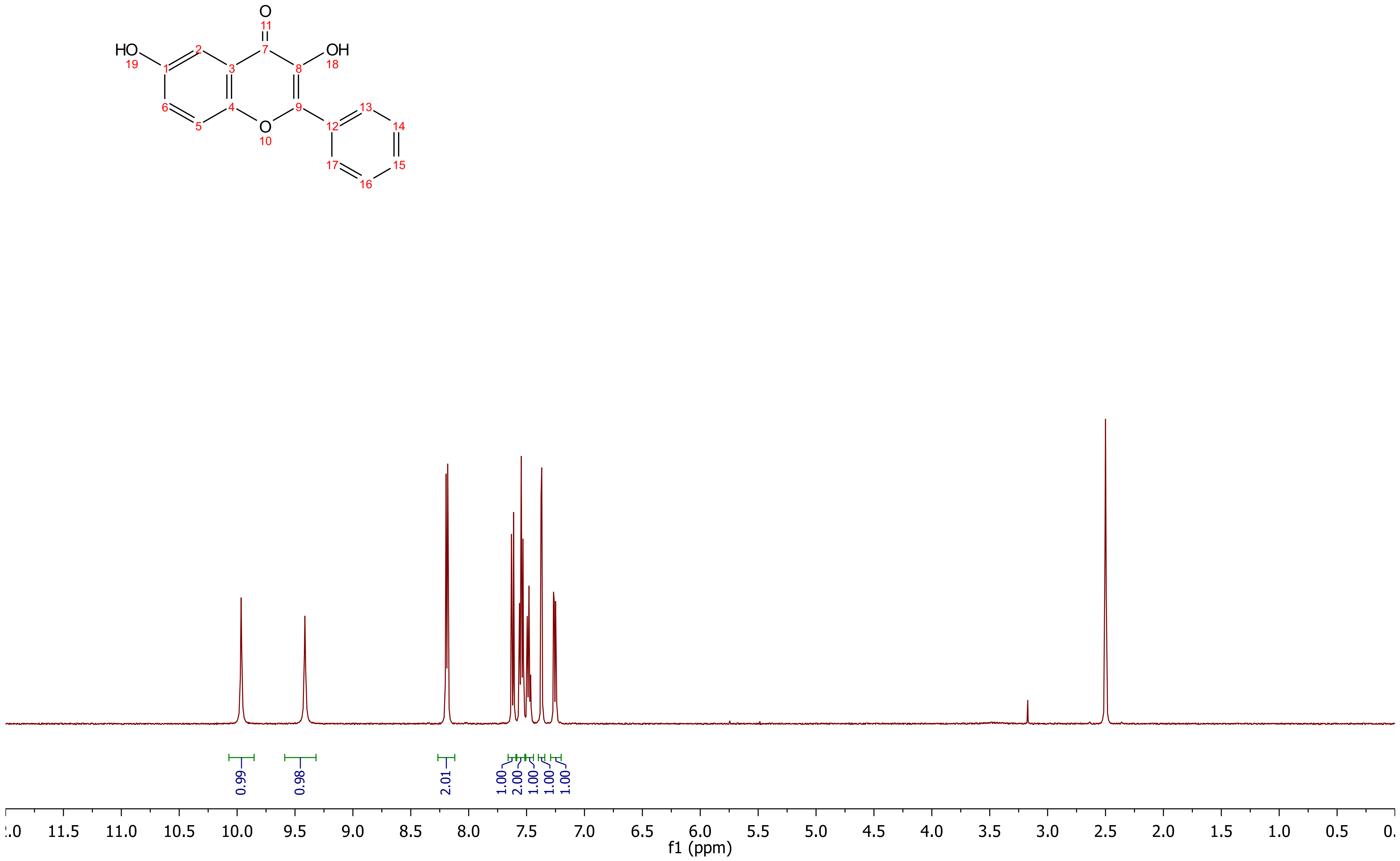

HO
OH
O
0.99
0.98
2.01
1.00
2.00
1.00
1.00
1.00
11.5
11.0
10.5
10.0
9.5
9.0
8.5
8.0
7.5
7.0
6.5
6.0
5.5
5.0
4.5
4.0
3.5
3.0
2.5
2.0
1.5
1.0
0.5
f1 (ppm)

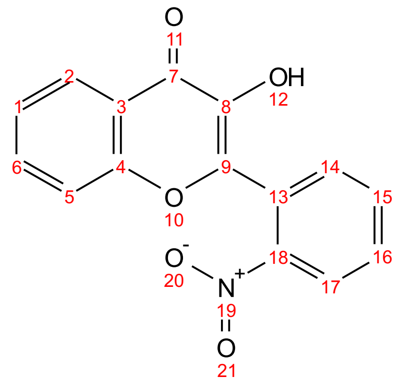
O
11
7
OH
12
8
9
1
2
3
4
5
6
O
10
13
14
15
16
17
18
O-
20
N+
19
O
21

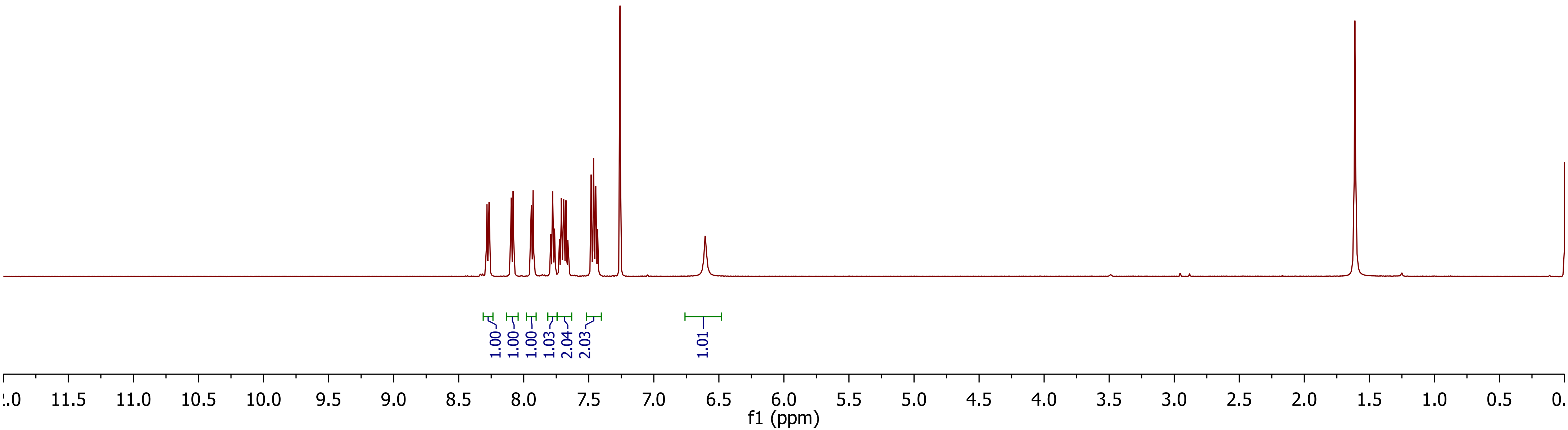
1.00
1.00
1.00
1.03
2.04
2.03
1.01
12.0
11.5
11.0
10.5
10.0
9.5
9.0
8.5
8.0
7.5
7.0
6.5
6.0
5.5
5.0
4.5
4.0
3.5
3.0
2.5
2.0
1.5
1.0
0.5
0.
f1 (ppm)

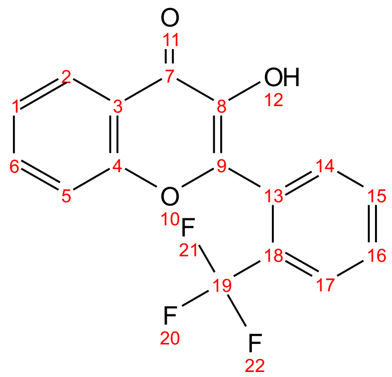
O
11
7
OH
12
1
2
3
8
6
4
9
14
15
5
O
10
13
F
21
18
16
19
17
F
20
F
22

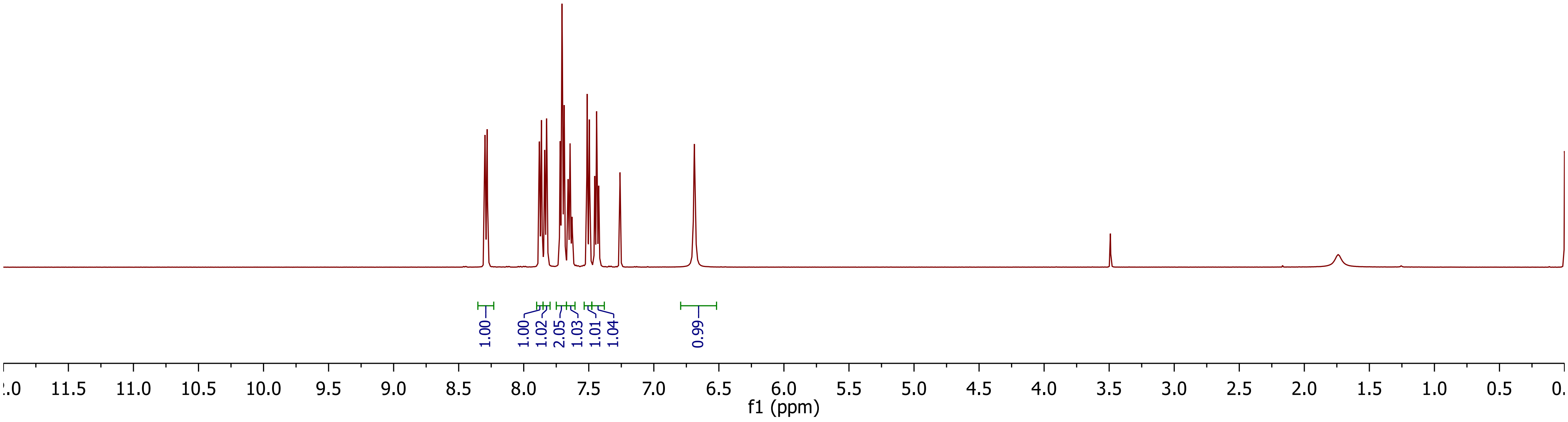
1.00
1.00
1.02
2.05
1.03
1.01
1.04
0.99
11.5
11.0
10.5
10.0
9.5
9.0
8.5
8.0
7.5
7.0
6.5
6.0
5.5
5.0
4.5
4.0
3.5
3.0
2.5
2.0
1.5
1.0
0.5
f1 (ppm)

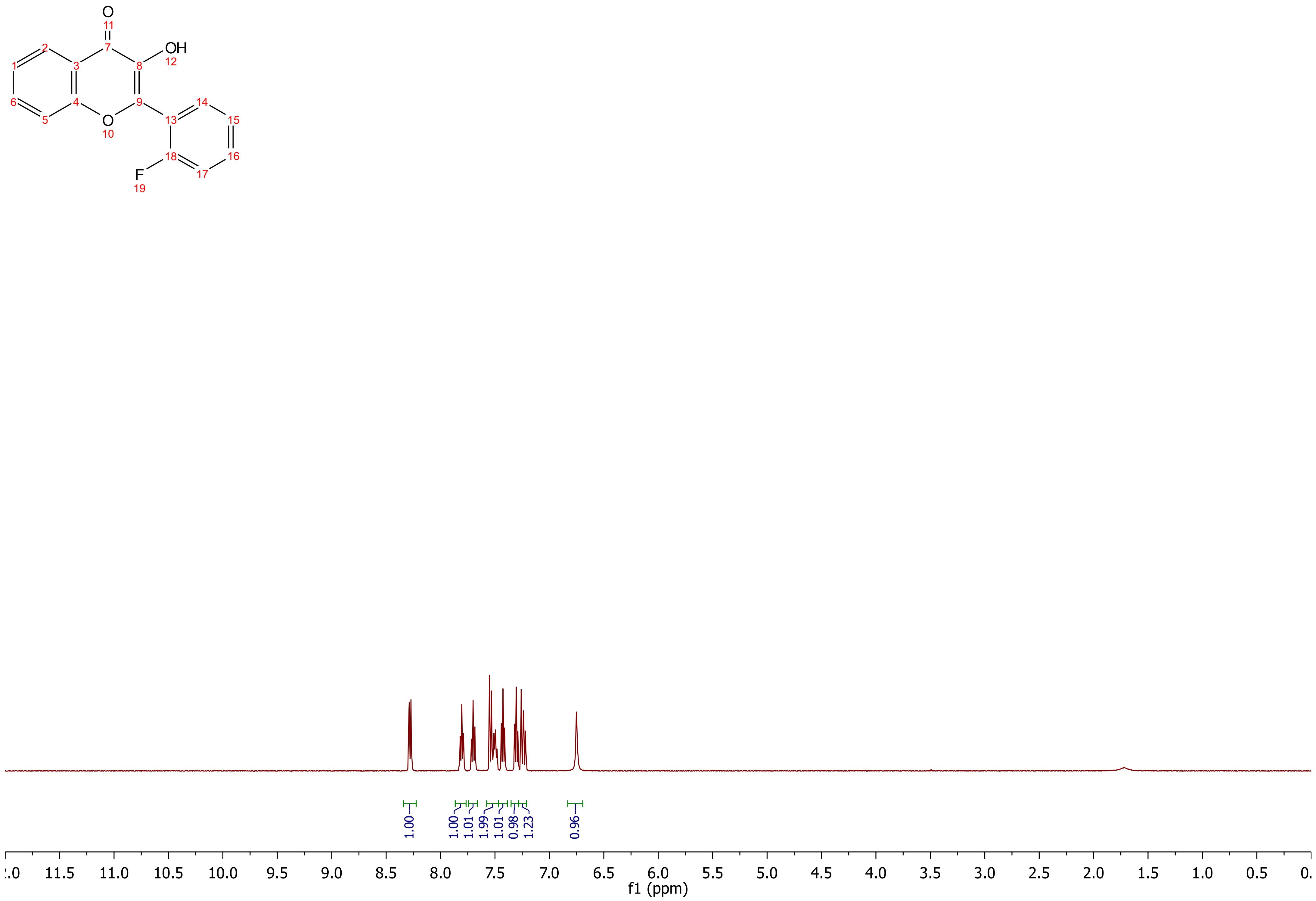

O
11
7
OH
12
8
9
O
10
F
19
1.00
1.00
1.01
1.99
1.01
0.98
1.23
0.96
11.5
11.0
10.5
10.0
9.5
9.0
8.5
8.0
7.5
7.0
6.5
6.0
5.5
5.0
4.5
4.0
3.5
3.0
2.5
2.0
1.5
1.0
0.5
f1 (ppm)

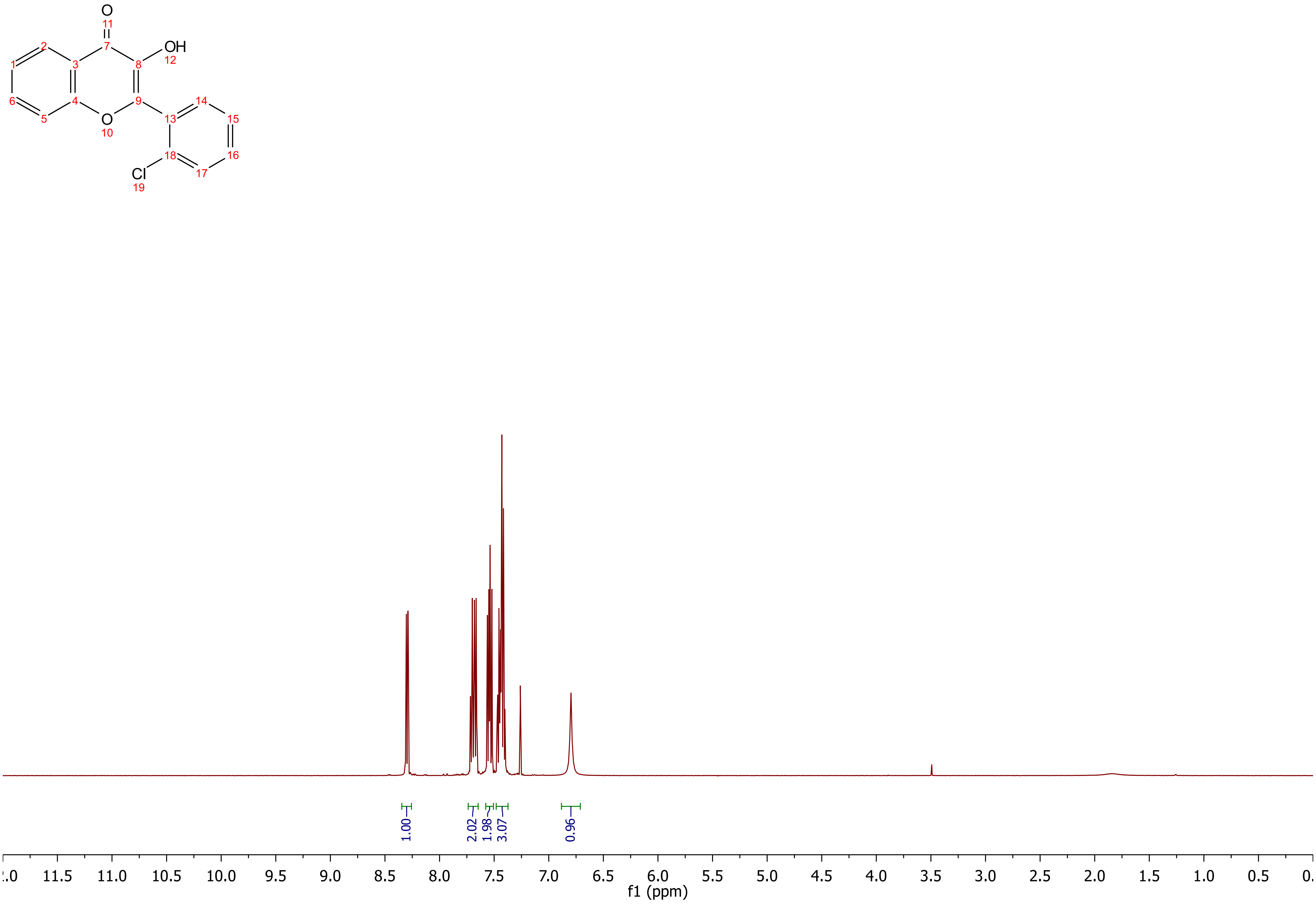

O
OH
O
Cl
1.00
2.02
1.98
3.07
0.96
11.5 11.0 10.5 10.0 9.5 9.0 8.5 8.0 7.5 7.0 6.5 6.0 5.5 5.0 4.5 4.0 3.5 3.0 2.5 2.0 1.5 1.0 0.5
f1 (ppm)

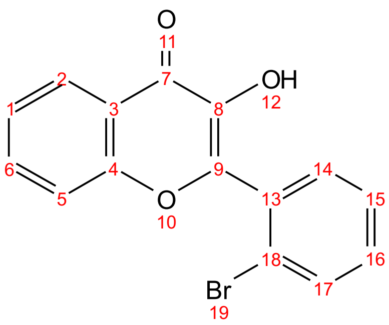

O
OH
O
Br

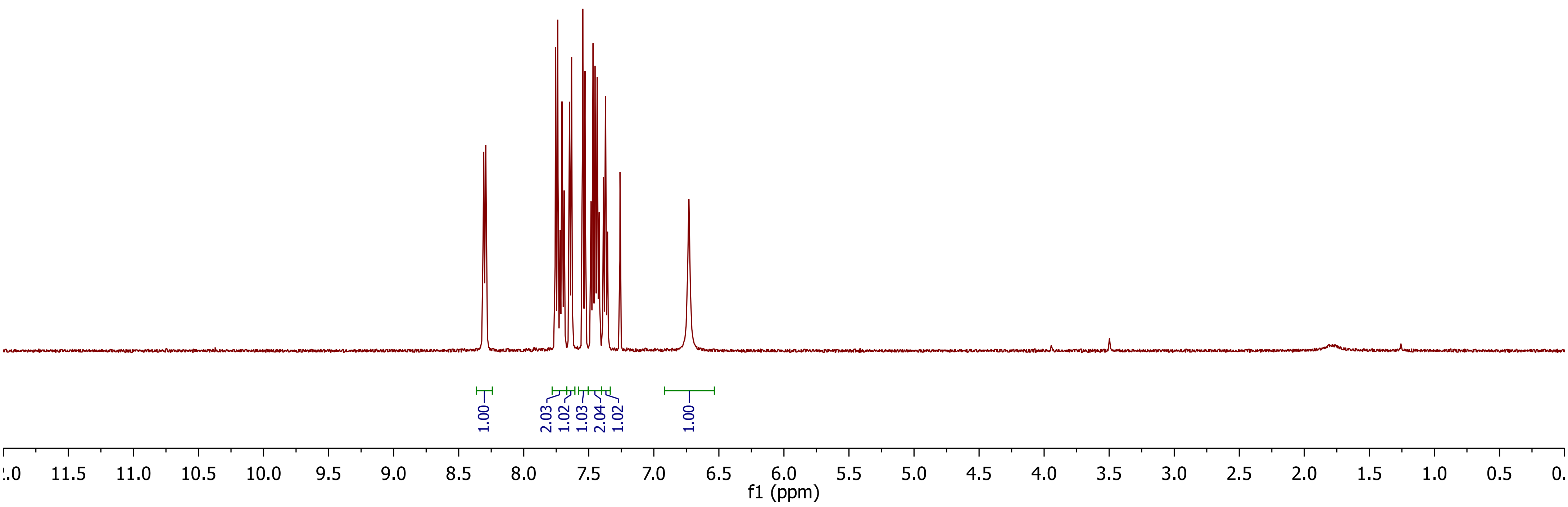

1.00
2.03
1.02
1.03
2.04
1.02
1.00
11.5 11.0 10.5 10.0 9.5 9.0 8.5 8.0 7.5 7.0 6.5 6.0 5.5 5.0 4.5 4.0 3.5 3.0 2.5 2.0 1.5 1.0 0.5
f1 (ppm)

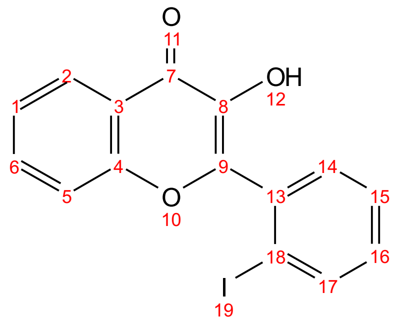

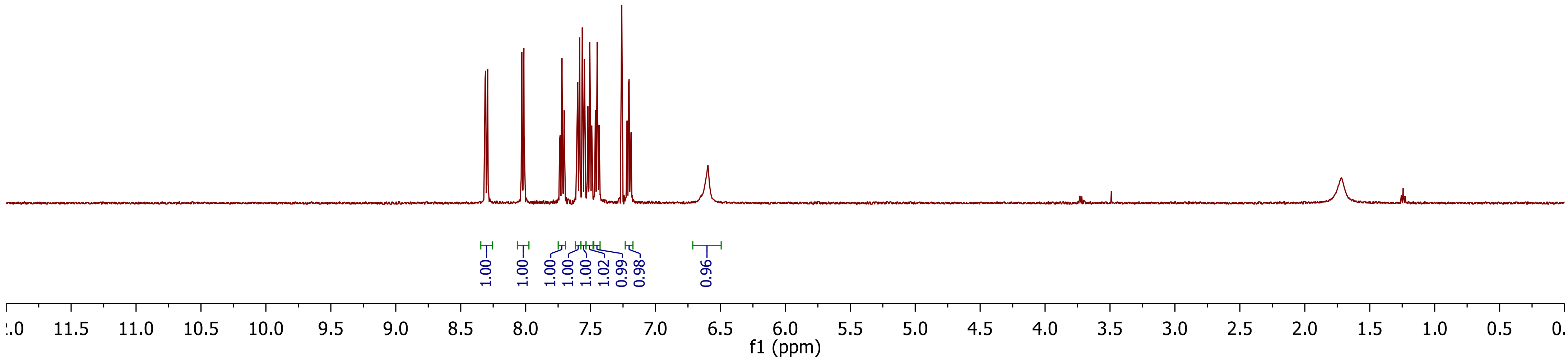

1.00
1.00
1.00
1.00
1.00
1.02
0.99
0.98
0.96
12.0
11.5
11.0
10.5
10.0
9.5
9.0
8.5
8.0
7.5
7.0
6.5
6.0
5.5
5.0
4.5
4.0
3.5
3.0
2.5
2.0
1.5
1.0
0.5
0.0
f1 (ppm)

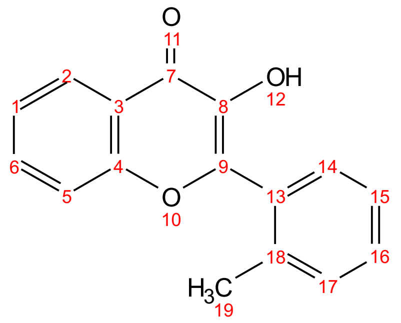
O
OH
O
H_3C

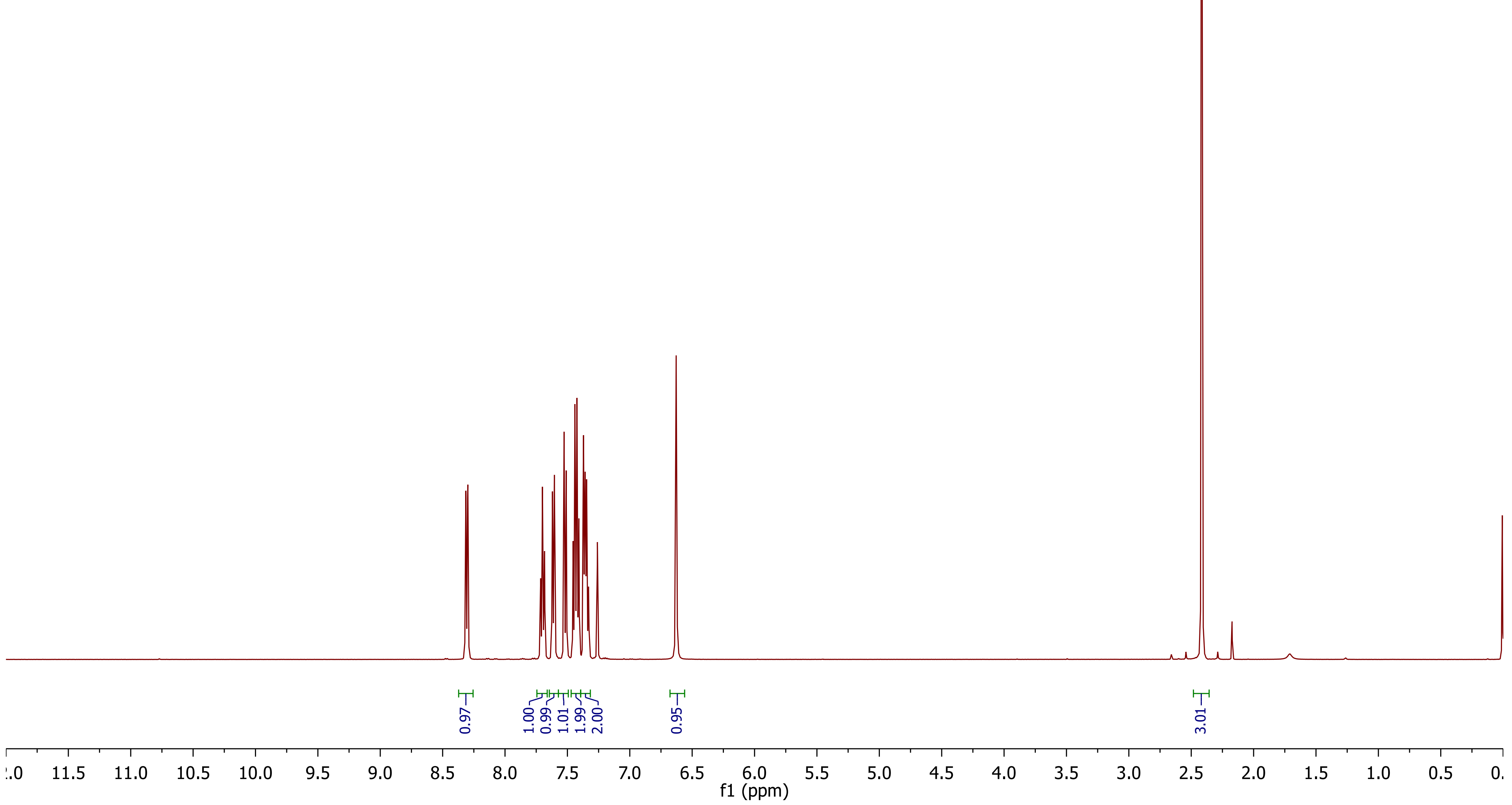
2.42
0.97
1.00
0.99
1.01
1.99
2.00
0.95
3.01
f1 (ppm)

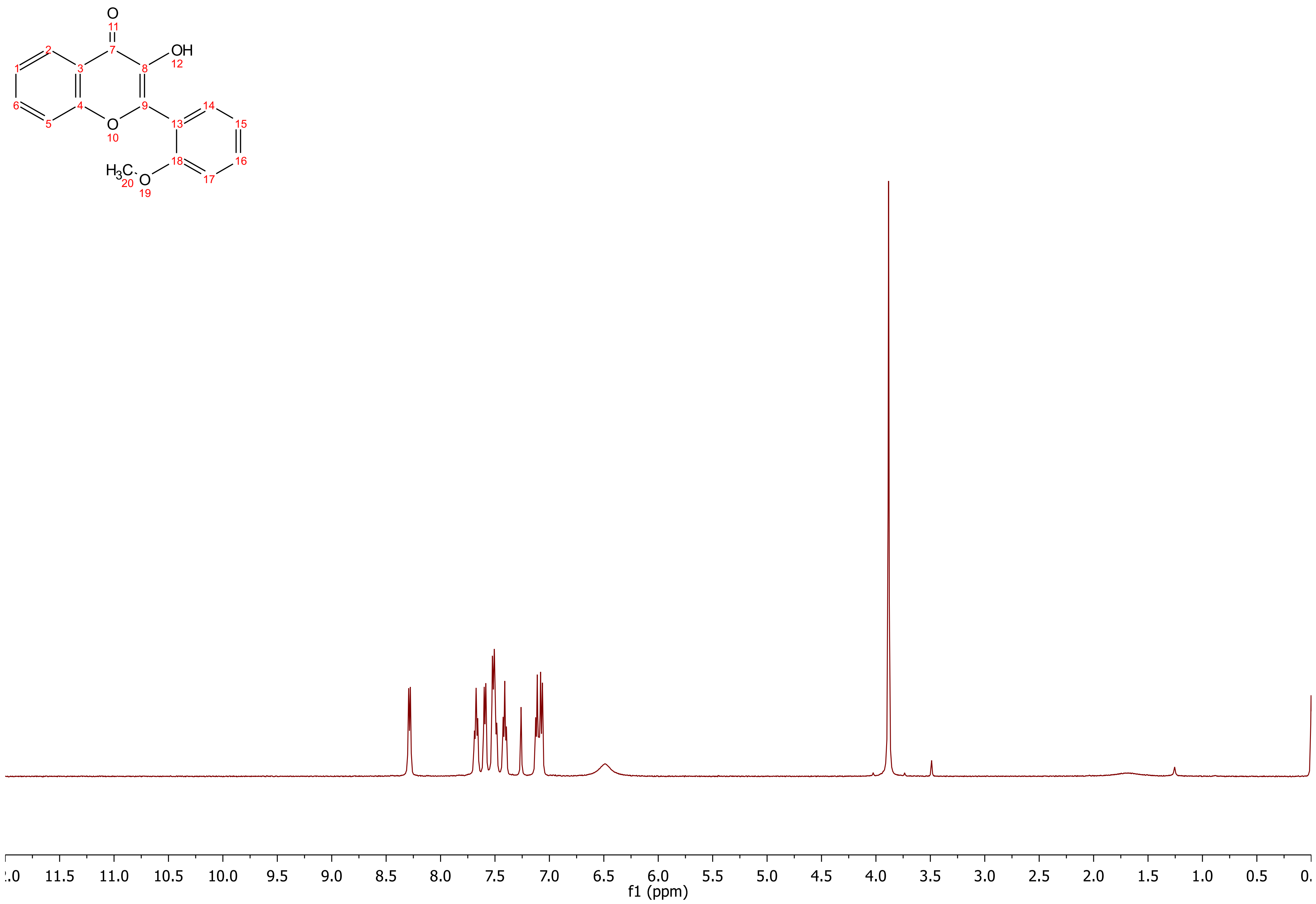

O
11
OH
12
1
2
3
4
5
6
7
8
9
O
10
13
14
15
16
17
18
H_3C
20
O
19
11.5
11.0
10.5
10.0
9.5
9.0
8.5
8.0
7.5
7.0
6.5
6.0
5.5
5.0
4.5
4.0
3.5
3.0
2.5
2.0
1.5
1.0
0.5
f1 (ppm)

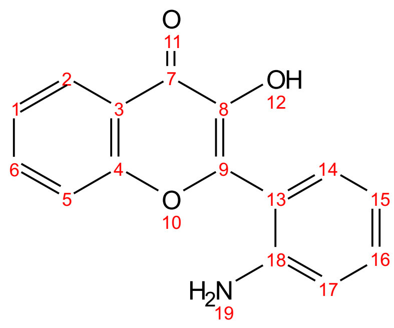

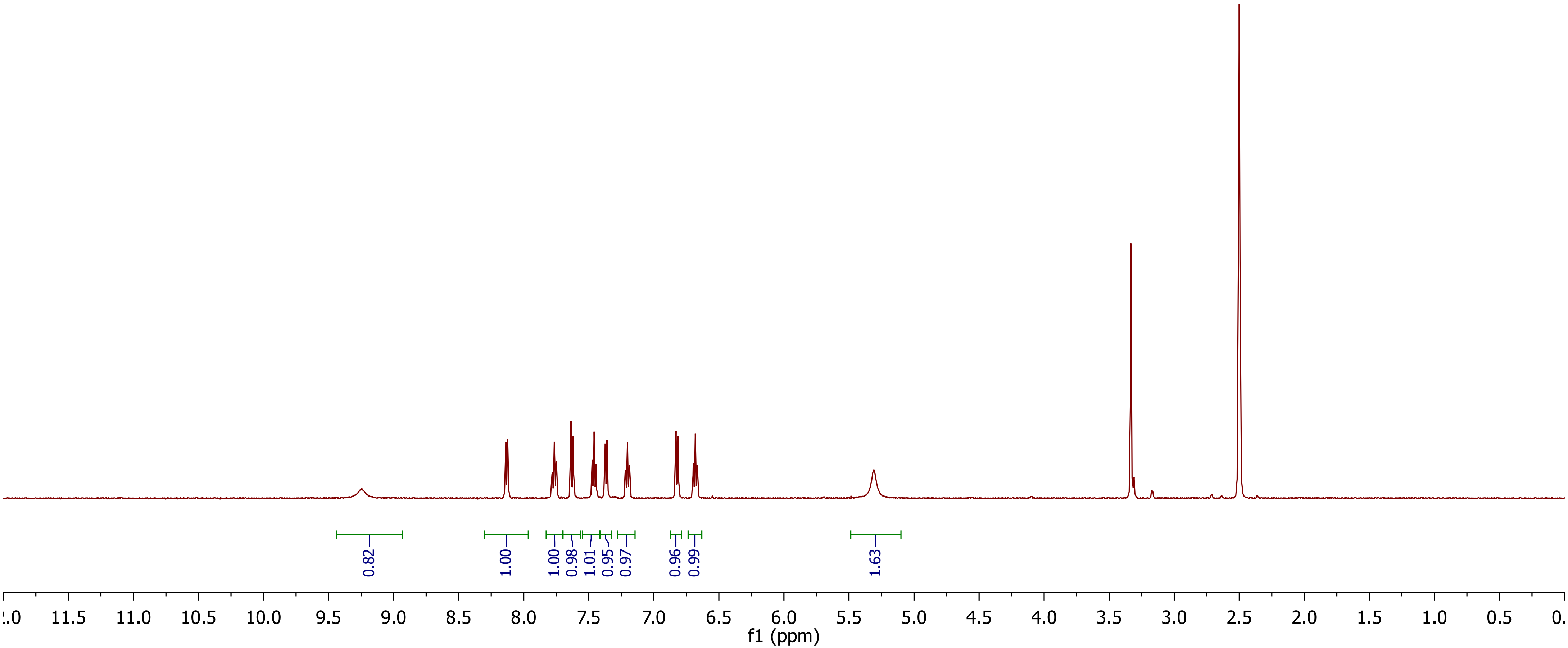

0.82
1.00
1.00
0.98
1.01
0.95
0.97
0.96
0.99
1.63
2.0
11.5
11.0
10.5
10.0
9.5
9.0
8.5
8.0
7.5
7.0
6.5
6.0
5.5
5.0
4.5
4.0
3.5
3.0
2.5
2.0
1.5
1.0
0.5
0.
f1 (ppm)

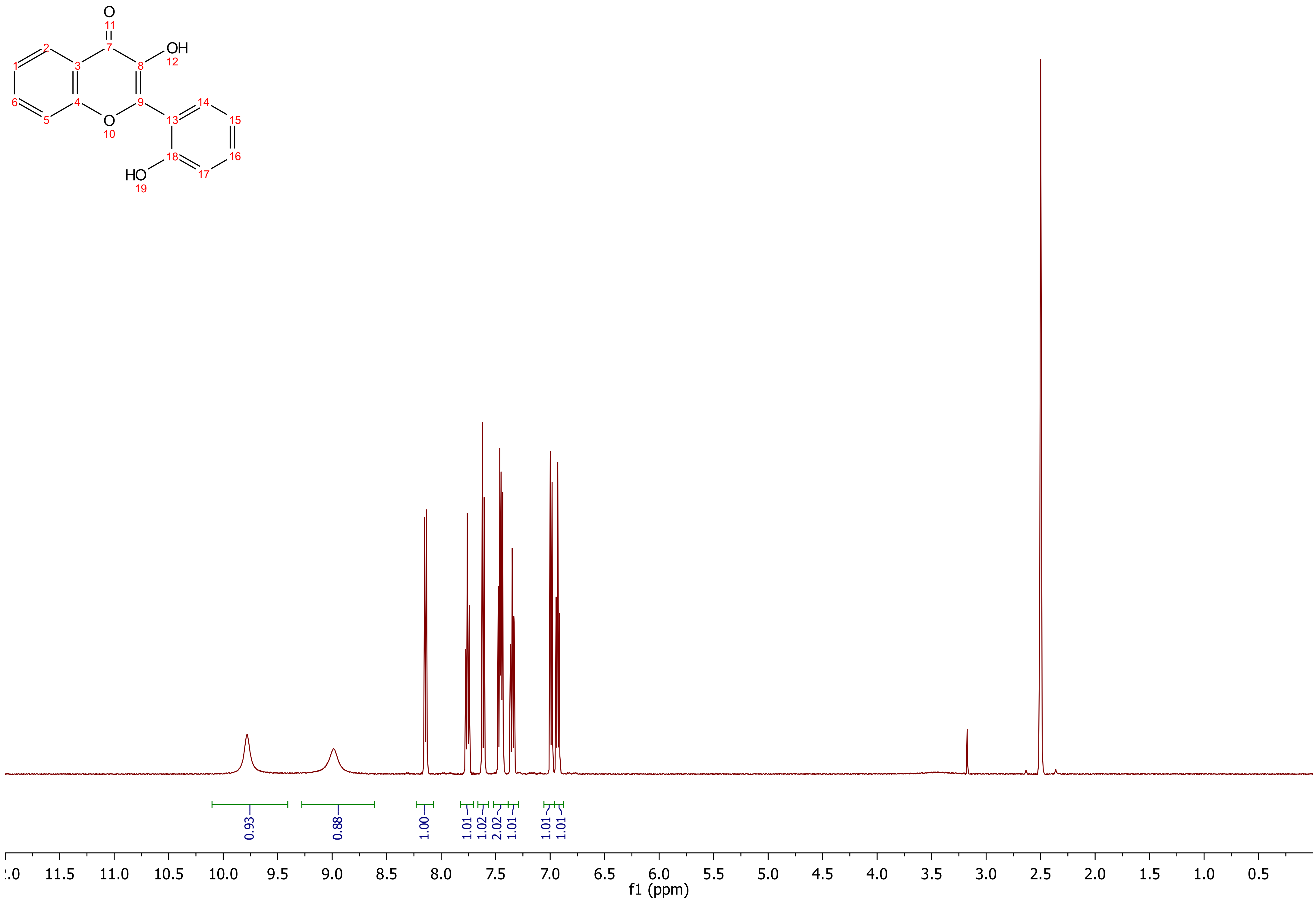

O
11
OH
12
1
2
3
4
5
6
7
8
9
O
10
13
14
15
16
17
18
HO
19
0.93
0.88
1.00
1.01
1.02
2.02
1.01
1.01
1.01
12.0 11.5 11.0 10.5 10.0 9.5 9.0 8.5 8.0 7.5 7.0 6.5 6.0 5.5 5.0 4.5 4.0 3.5 3.0 2.5 2.0 1.5 1.0 0.5
f1 (ppm)

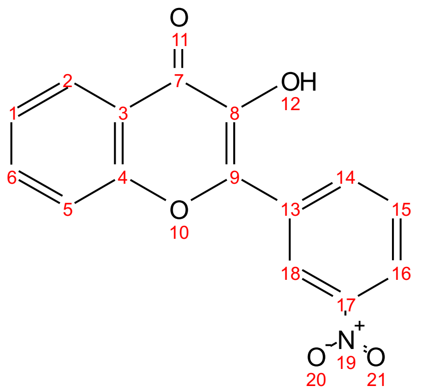

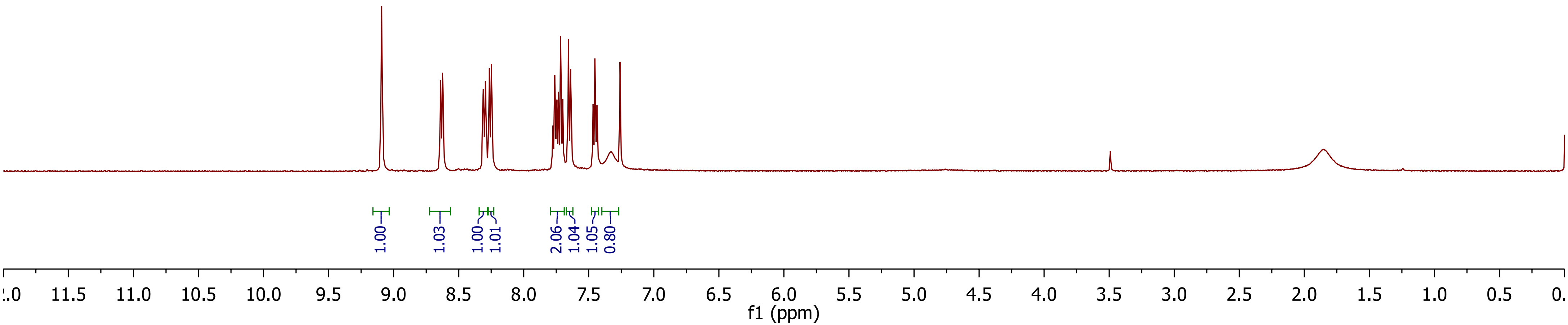
1.00
1.03
1.00
1.01
2.06
1.04
1.05
0.80
11.5
11.0
10.5
10.0
9.5
9.0
8.5
8.0
7.5
7.0
6.5
6.0
5.5
5.0
4.5
4.0
3.5
3.0
2.5
2.0
1.5
1.0
0.5
f1 (ppm)

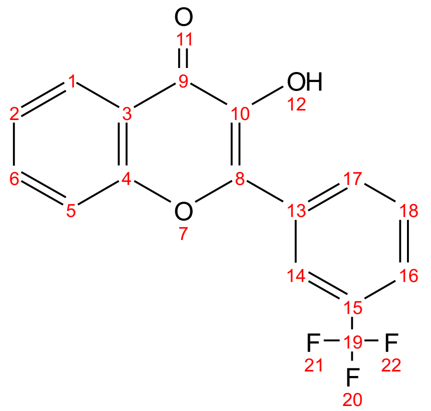

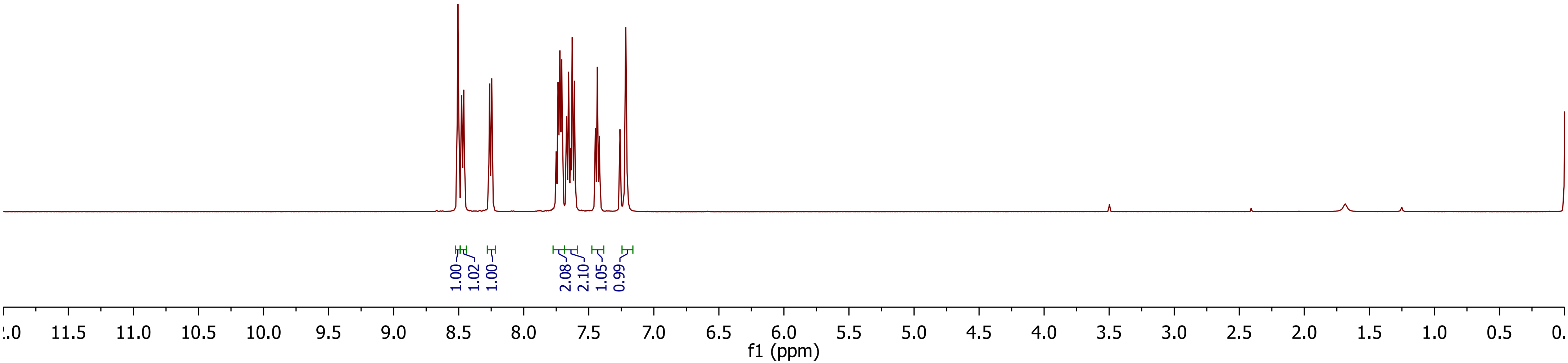

1.00
1.02
1.00
2.08
2.10
1.05
0.99
12.0
11.5
11.0
10.5
10.0
9.5
9.0
8.5
8.0
7.5
7.0
6.5
6.0
5.5
5.0
4.5
4.0
3.5
3.0
2.5
2.0
1.5
1.0
0.5
0.
f1 (ppm)

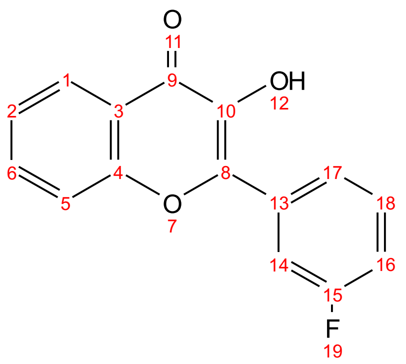
O
11
OH
12
1
2
3
4
5
6
7
8
9
10
13
14
15
16
17
18
F
19

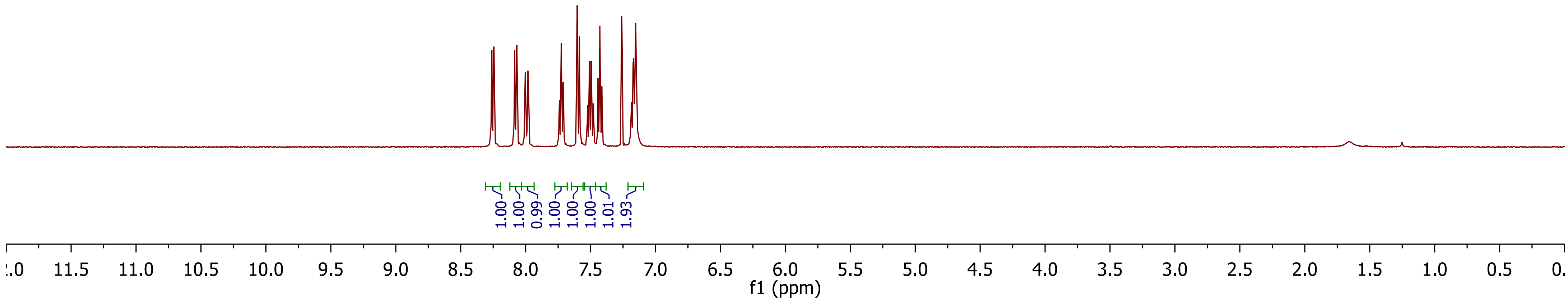
1.00
1.00
0.99
1.00
1.00
1.00
1.01
1.93
11.5
11.0
10.5
10.0
9.5
9.0
8.5
8.0
7.5
7.0
6.5
6.0
5.5
5.0
4.5
4.0
3.5
3.0
2.5
2.0
1.5
1.0
0.5
f1 (ppm)

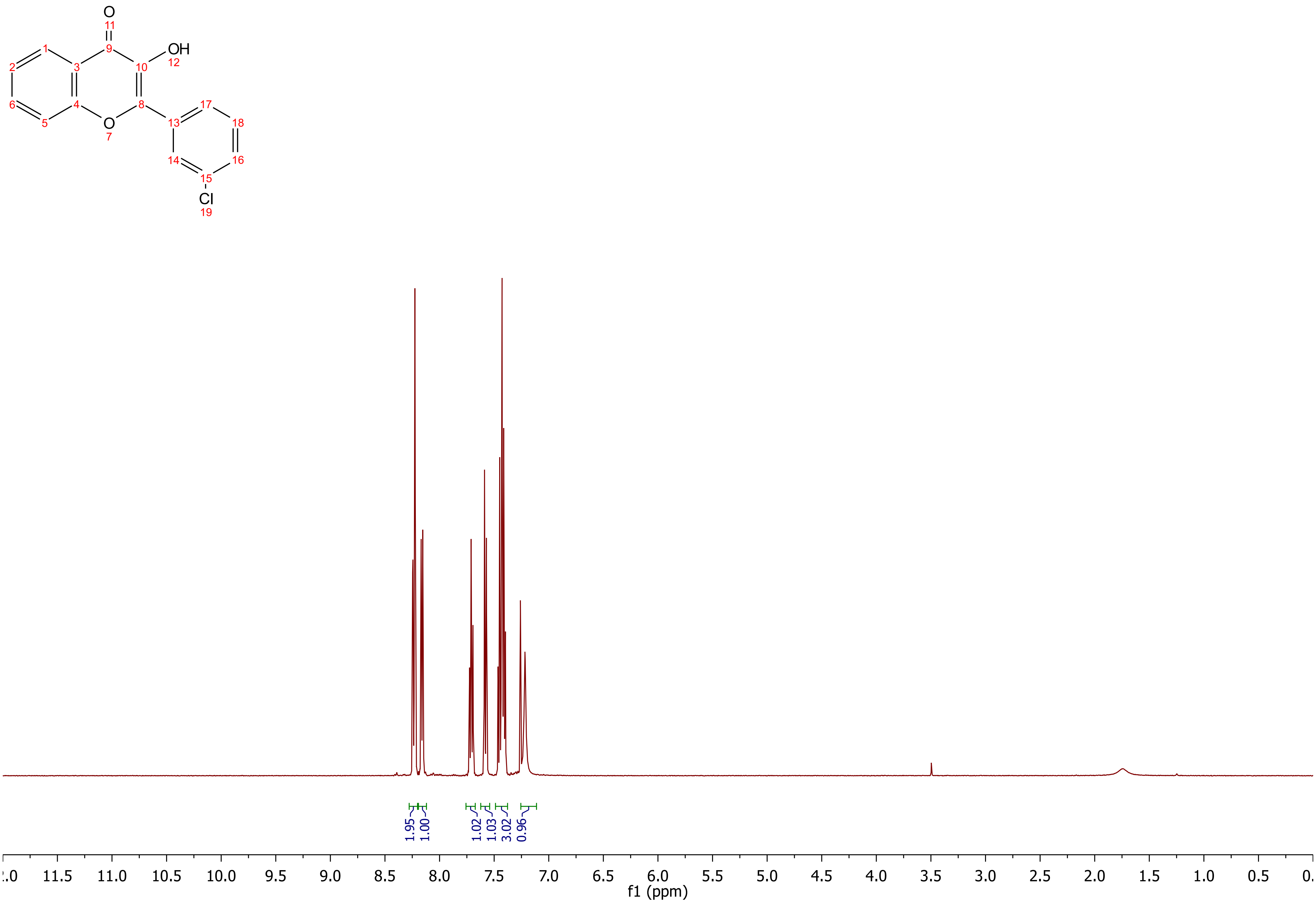

O
OH
O
Cl
1.95
1.00
1.02
1.03
3.02
0.96
11.5
11.0
10.5
10.0
9.5
9.0
8.5
8.0
7.5
7.0
6.5
6.0
5.5
5.0
4.5
4.0
3.5
3.0
2.5
2.0
1.5
1.0
0.5
f1 (ppm)

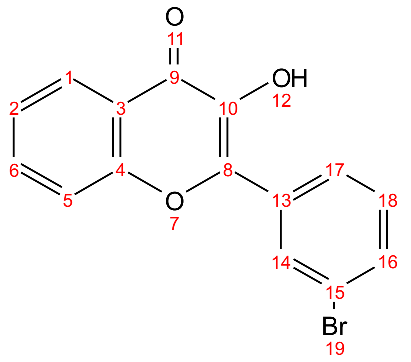

O
11
9
1
2
3
10
OH
12
6
4
8
17
18
5
O
7
13
14
16
15
Br
19

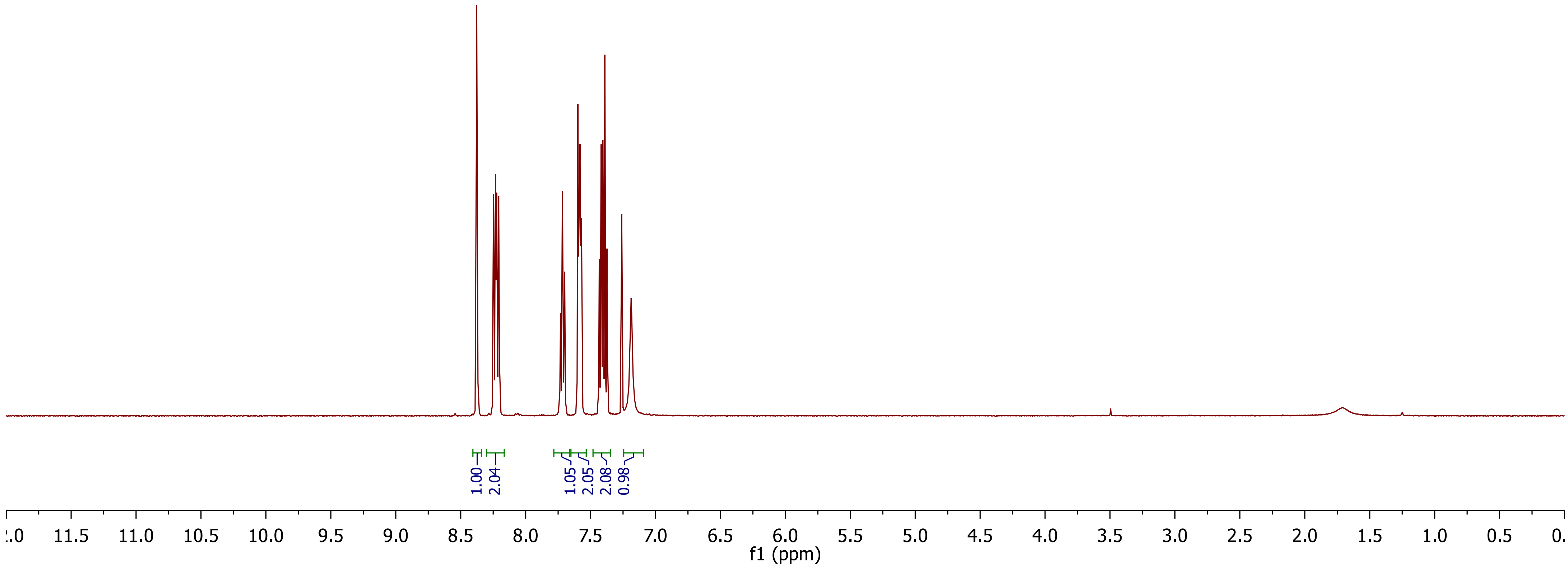

1.00
2.04
1.05
2.05
2.08
0.98
11.5
11.0
10.5
10.0
9.5
9.0
8.5
8.0
7.5
7.0
6.5
6.0
5.5
5.0
4.5
4.0
3.5
3.0
2.5
2.0
1.5
1.0
0.5
f1 (ppm)

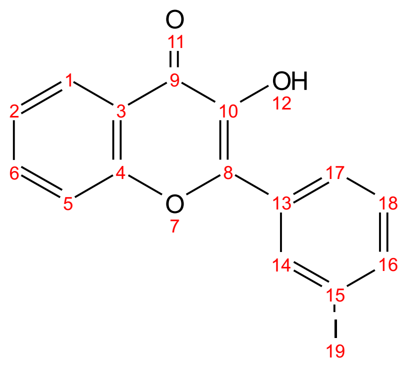
O
11
9
OH
12
10
1
2
3
6
4
8
17
5
O
7
13
18
14
16
15
19

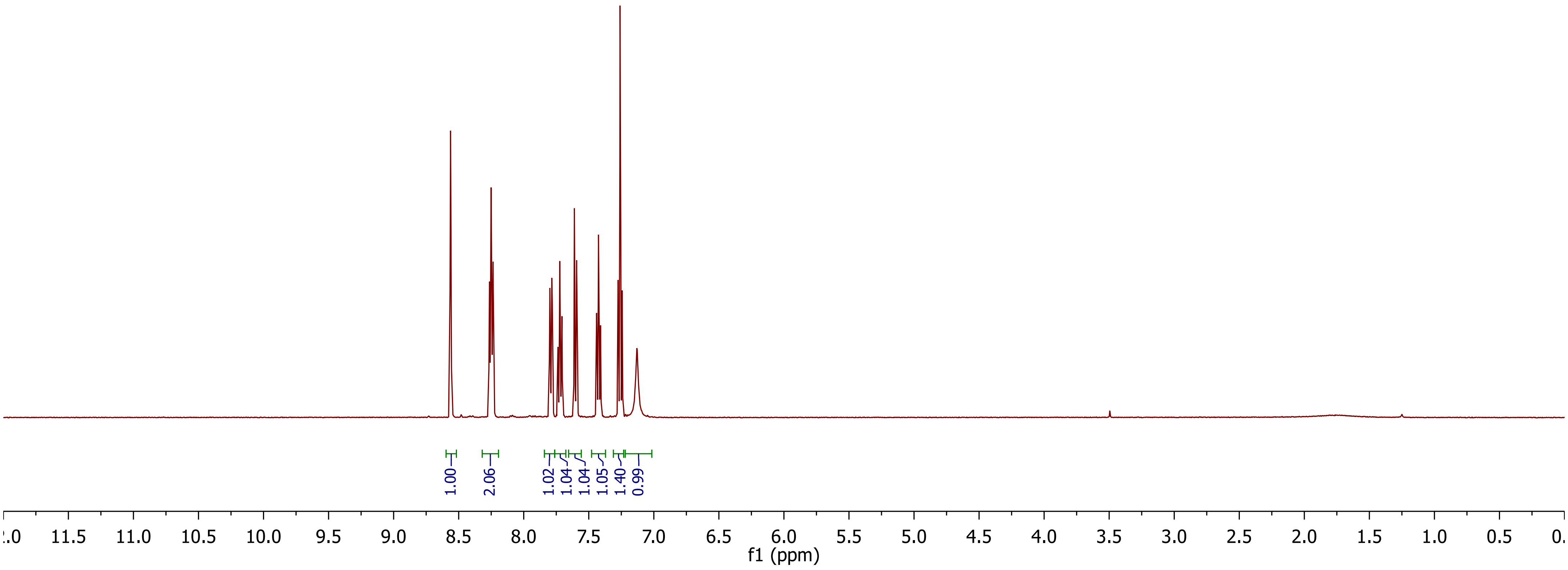
1.00
2.06
1.02
1.04
1.04
1.05
1.40
0.99
11.5
11.0
10.5
10.0
9.5
9.0
8.5
8.0
7.5
7.0
6.5
6.0
5.5
5.0
4.5
4.0
3.5
3.0
2.5
2.0
1.5
1.0
0.5
f1 (ppm)

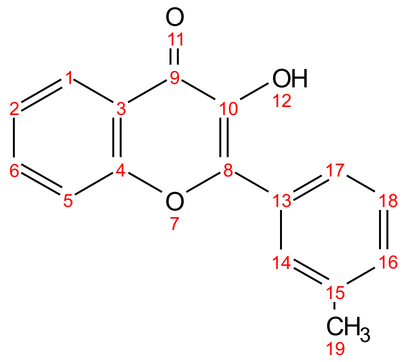
O
11
OH
12
1
2
3
9
10
6
4
8
17
18
5
O
7
13
14
16
15
CH3
19
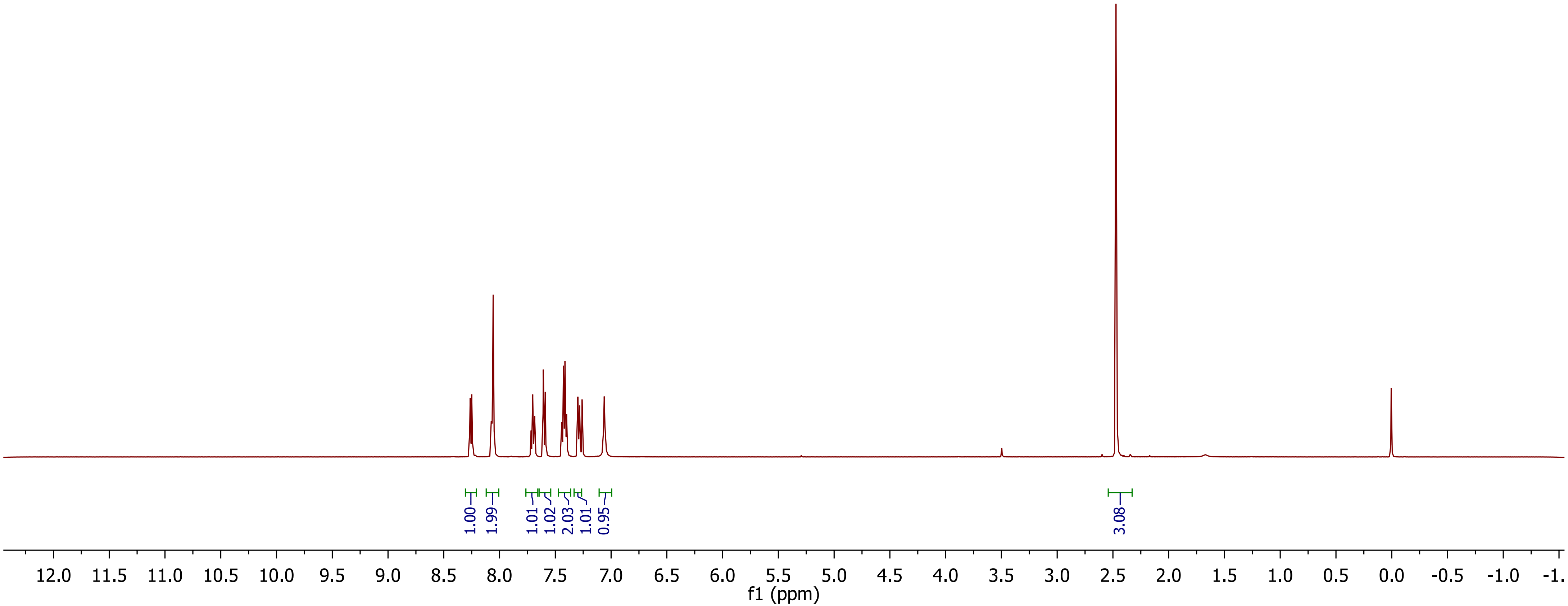
1.00
1.99
1.01
1.02
2.03
1.01
0.95
3.08
12.0
11.5
11.0
10.5
10.0
9.5
9.0
8.5
8.0
7.5
7.0
6.5
6.0
5.5
5.0
4.5
4.0
3.5
3.0
2.5
2.0
1.5
1.0
0.5
0.0
-0.5
-1.0
-1.
f1 (ppm)

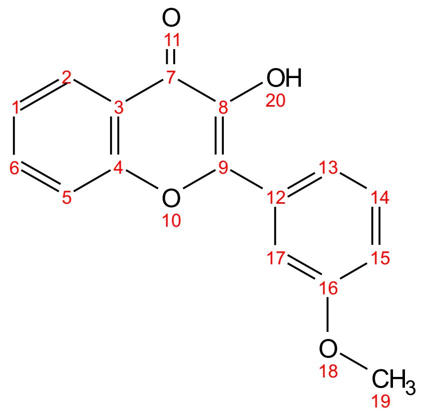
O
11
7
OH
20
8
9
O
10
1
2
3
4
5
6
12
13
14
15
16
17
O
18
CH3
19

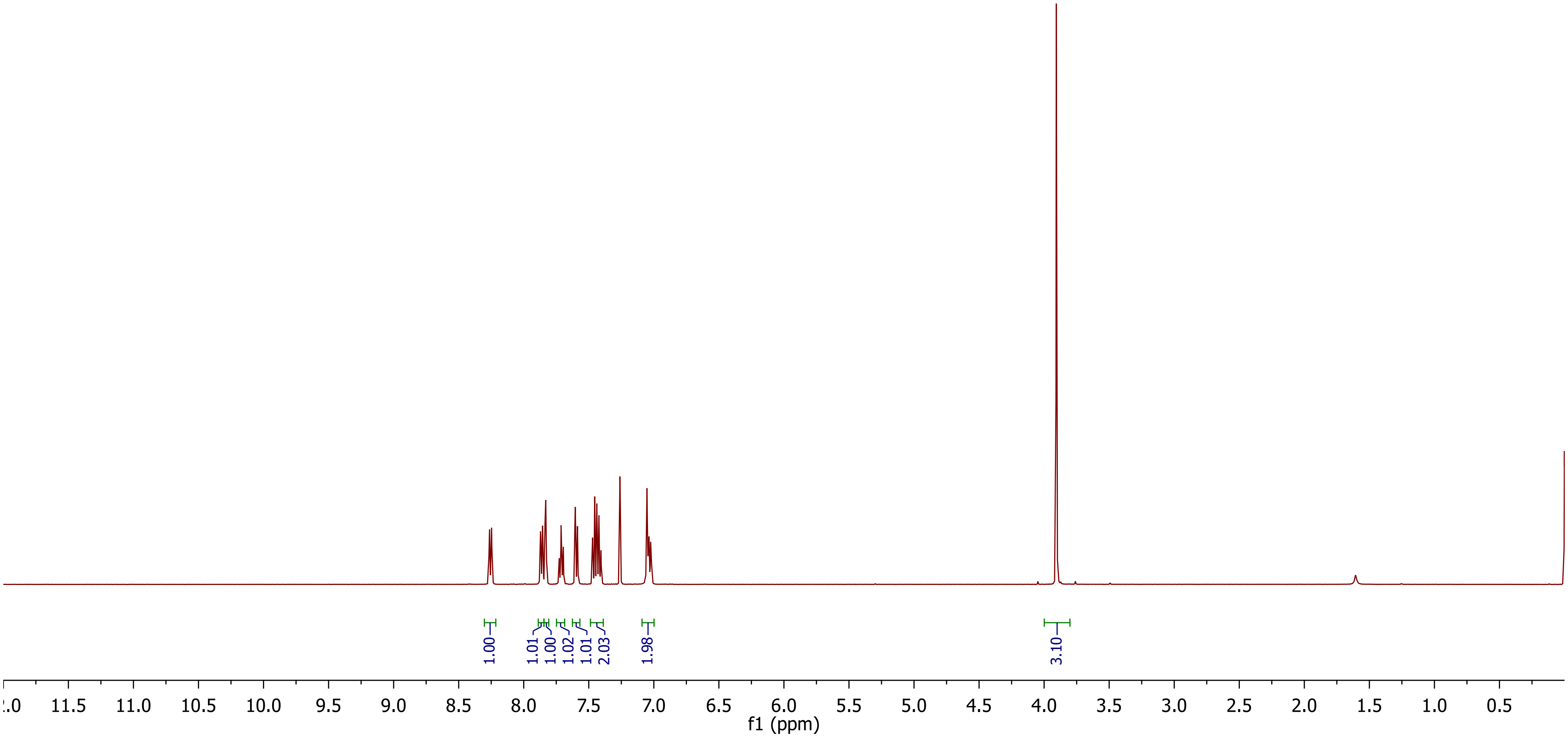
1.00
1.01
1.00
1.02
1.01
2.03
1.98
3.10
11.5
11.0
10.5
10.0
9.5
9.0
8.5
8.0
7.5
7.0
6.5
6.0
5.5
5.0
4.5
4.0
3.5
3.0
2.5
2.0
1.5
1.0
0.5
f1 (ppm)

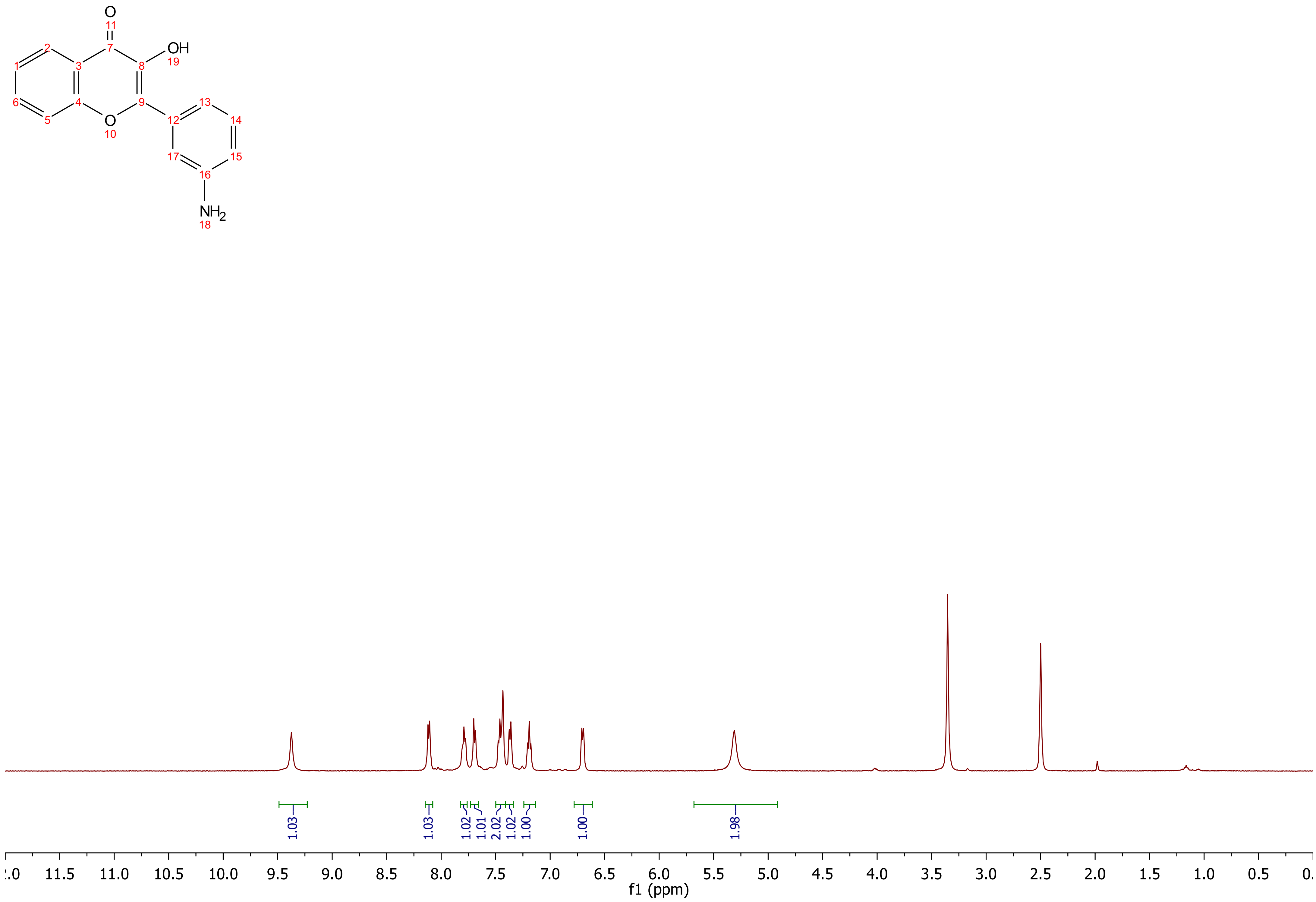

O
OH
NH2
1.03
1.03
1.02
1.01
2.02
1.02
1.00
1.00
1.98
f1 (ppm)

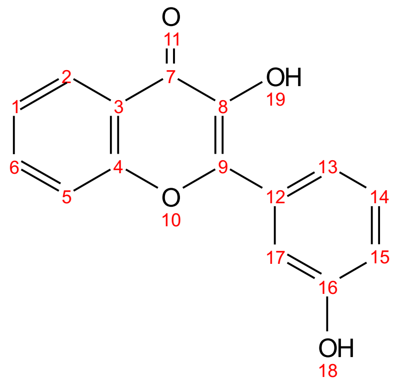
O
11
7
OH
19
8
9
2
1
3
6
4
5
O
10
13
14
12
15
17
16
OH
18

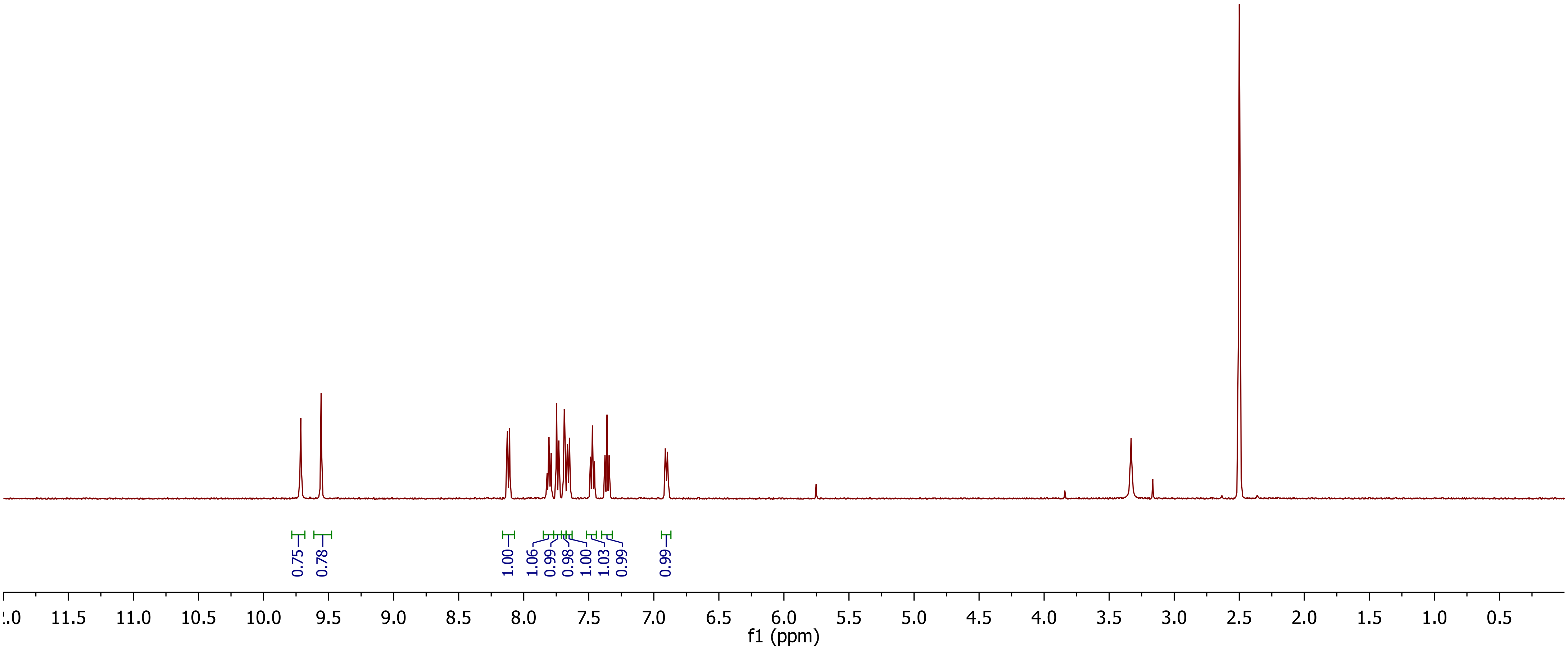
0.75
0.78
1.00
1.06
0.99
0.98
1.00
1.03
0.99
0.99
11.5
11.0
10.5
10.0
9.5
9.0
8.5
8.0
7.5
7.0
6.5
6.0
5.5
5.0
4.5
4.0
3.5
3.0
2.5
2.0
1.5
1.0
0.5
f1 (ppm)

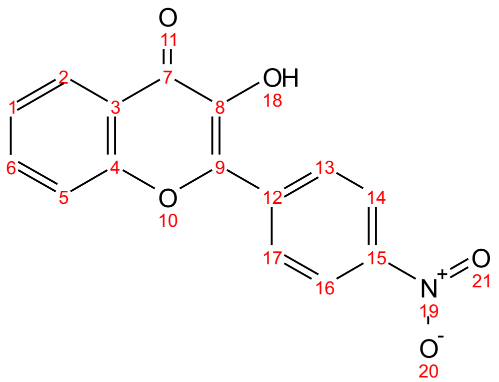
O
OH
O
N+
O-

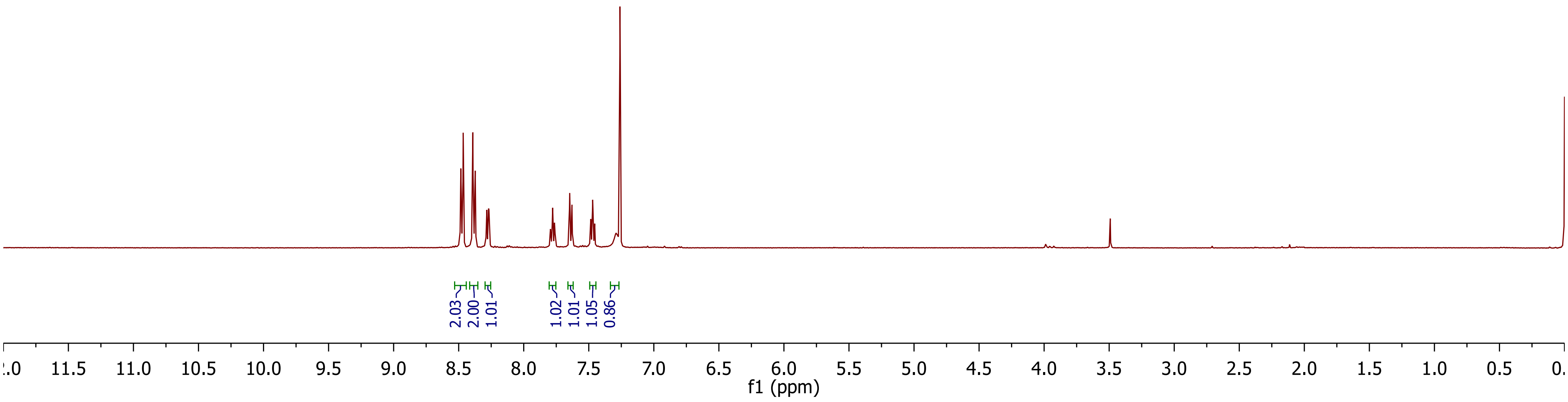
2.03
2.00
1.01
1.02
1.01
1.05
0.86
f1 (ppm)

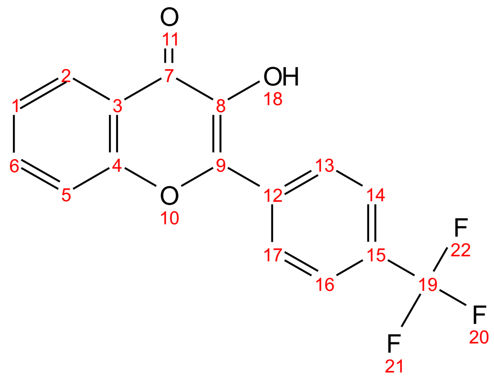

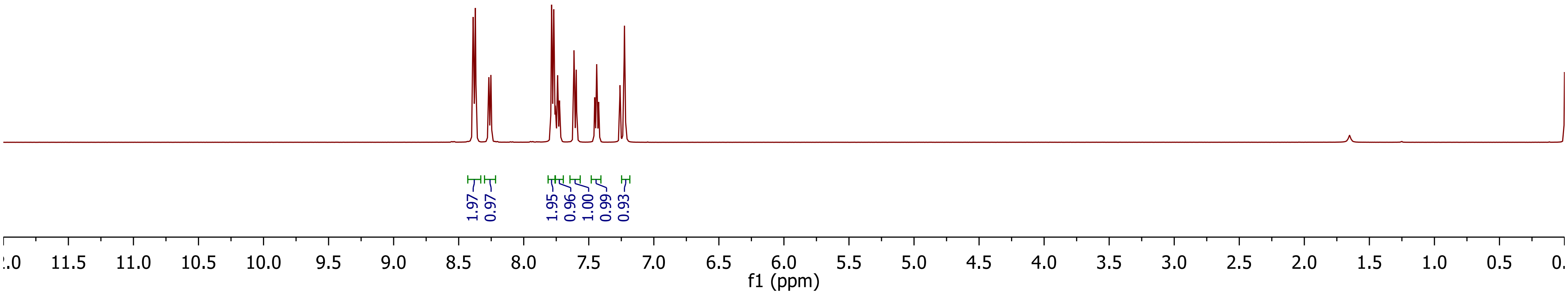
1.97
0.97
1.95
0.96
1.00
0.99
0.93
11.5 11.0 10.5 10.0 9.5 9.0 8.5 8.0 7.5 7.0 6.5 6.0 5.5 5.0 4.5 4.0 3.5 3.0 2.5 2.0 1.5 1.0 0.5
f1 (ppm)

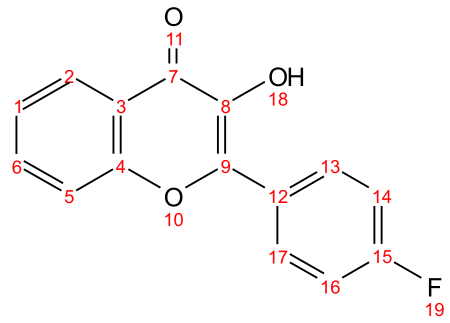

O
11
7
OH
18
8
2
1
3
6
4
9
13
5
O
10
12
14
17
15
16
F
19

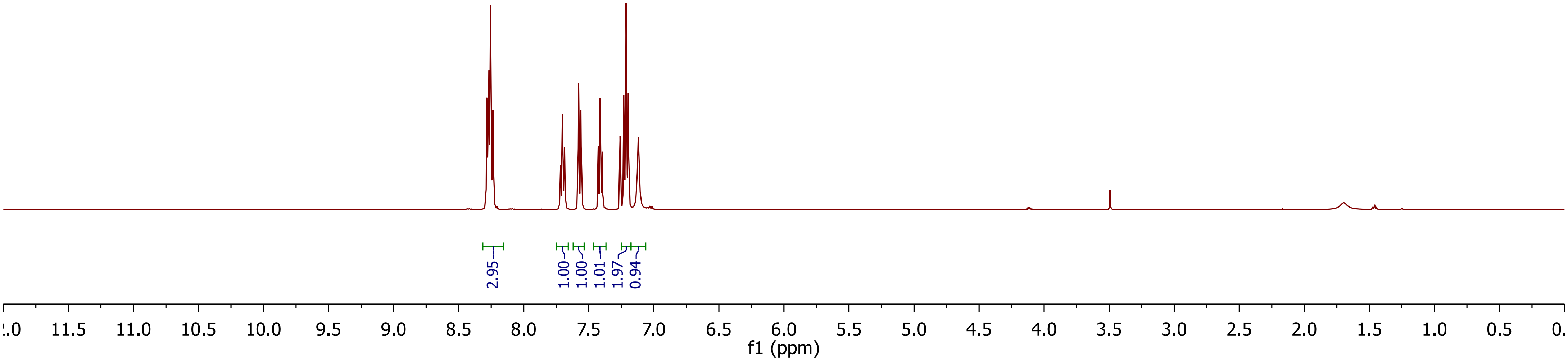

2.95
1.00
1.00
1.01
1.97
0.94
11.5
11.0
10.5
10.0
9.5
9.0
8.5
8.0
7.5
7.0
6.5
6.0
5.5
5.0
4.5
4.0
3.5
3.0
2.5
2.0
1.5
1.0
0.5
f1 (ppm)

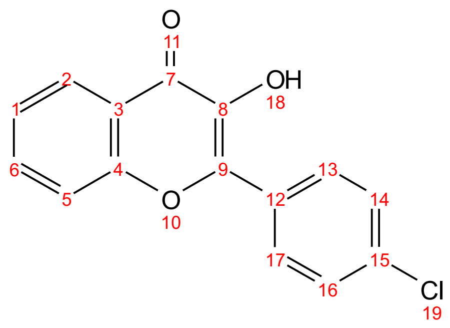

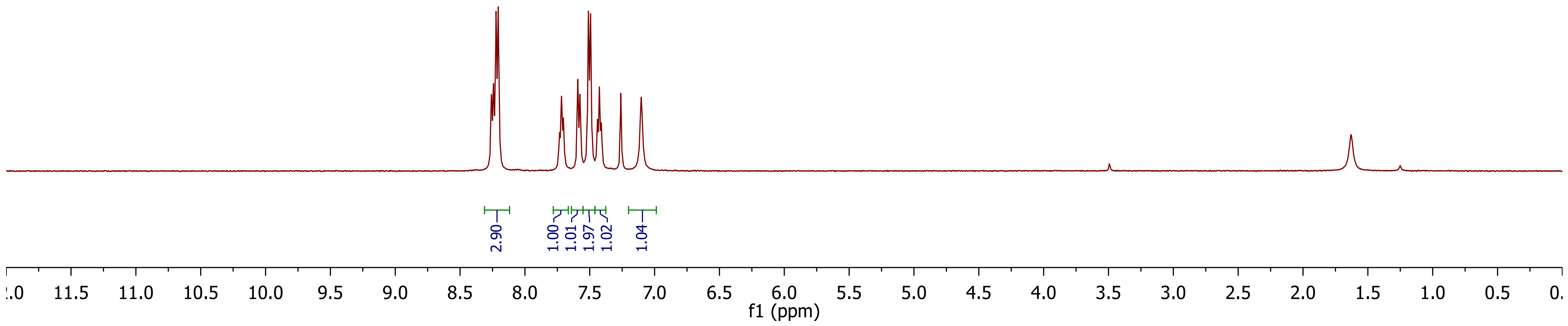

2.90
1.00
1.01
1.97
1.02
1.04
11.5
11.0
10.5
10.0
9.5
9.0
8.5
8.0
7.5
7.0
6.5
6.0
5.5
5.0
4.5
4.0
3.5
3.0
2.5
2.0
1.5
1.0
0.5
f1 (ppm)

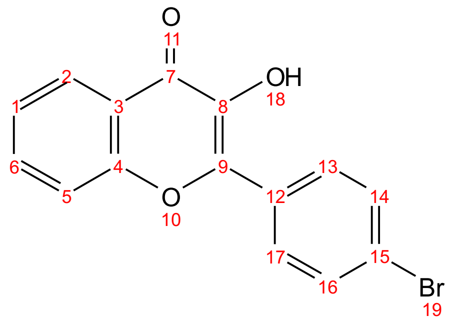
O
11
7
OH
18
8
9
O
10
1
2
3
4
5
6
12
13
14
15
16
17
Br
19

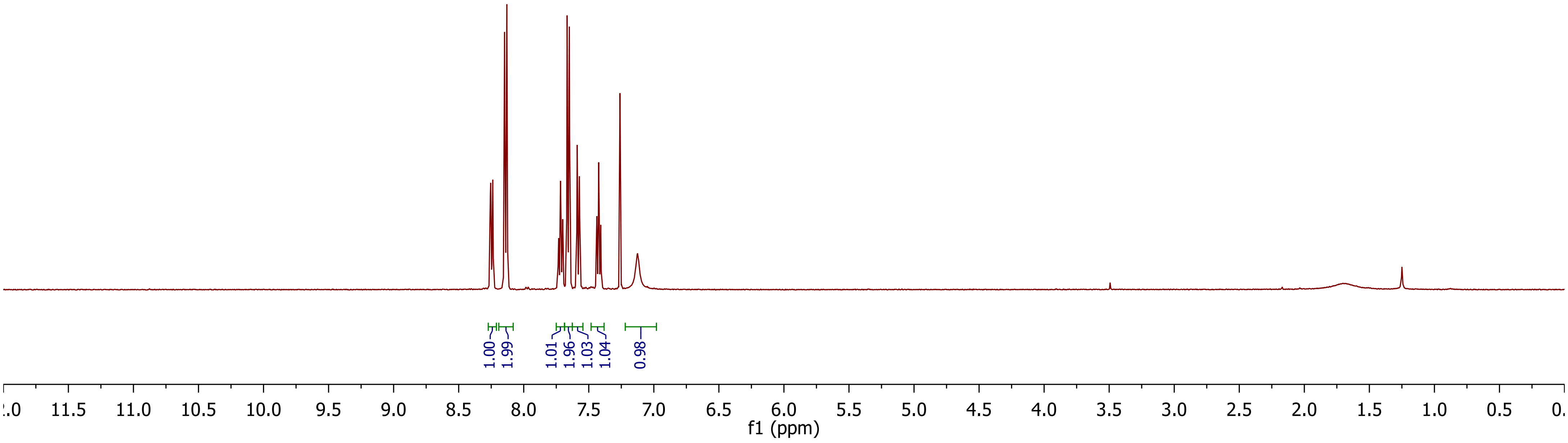
1.00
1.99
1.01
1.96
1.03
1.04
0.98
11.5
11.0
10.5
10.0
9.5
9.0
8.5
8.0
7.5
7.0
6.5
6.0
5.5
5.0
4.5
4.0
3.5
3.0
2.5
2.0
1.5
1.0
0.5
f1 (ppm)

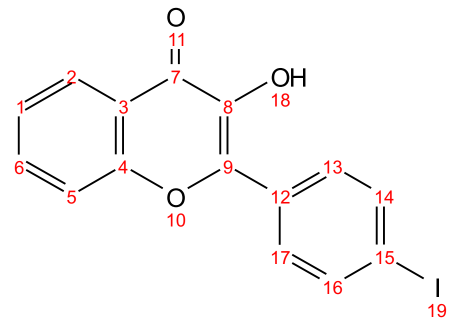
O
OH
O
I
11
7
8
18
1
2
3
4
5
6
9
10
12
13
14
15
16
17
19

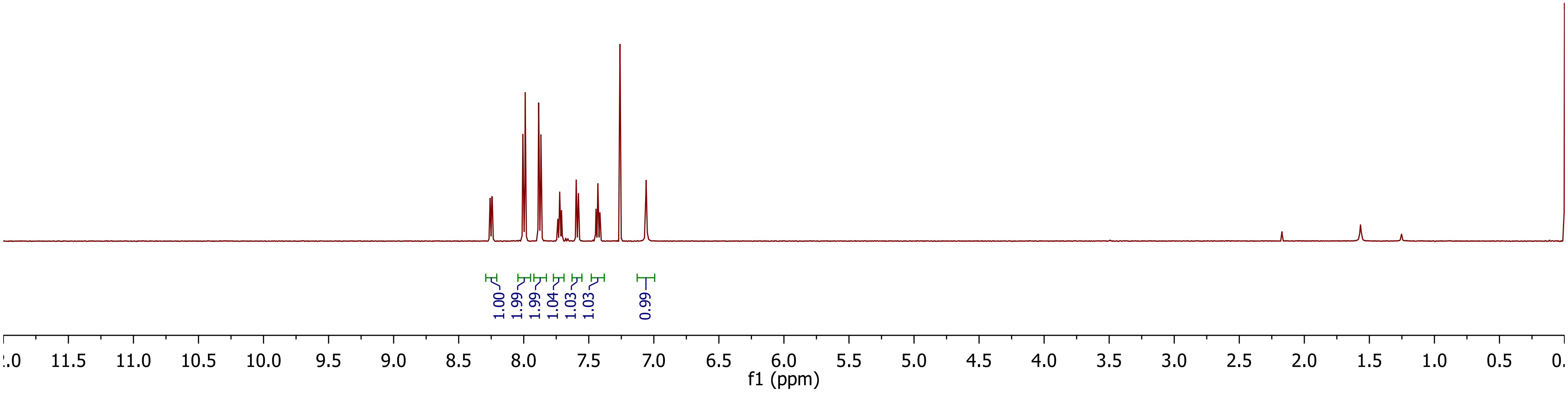
1.00
1.99
1.99
1.04
1.03
1.03
0.99
11.5
11.0
10.5
10.0
9.5
9.0
8.5
8.0
7.5
7.0
6.5
6.0
5.5
5.0
4.5
4.0
3.5
3.0
2.5
2.0
1.5
1.0
0.5
f1 (ppm)

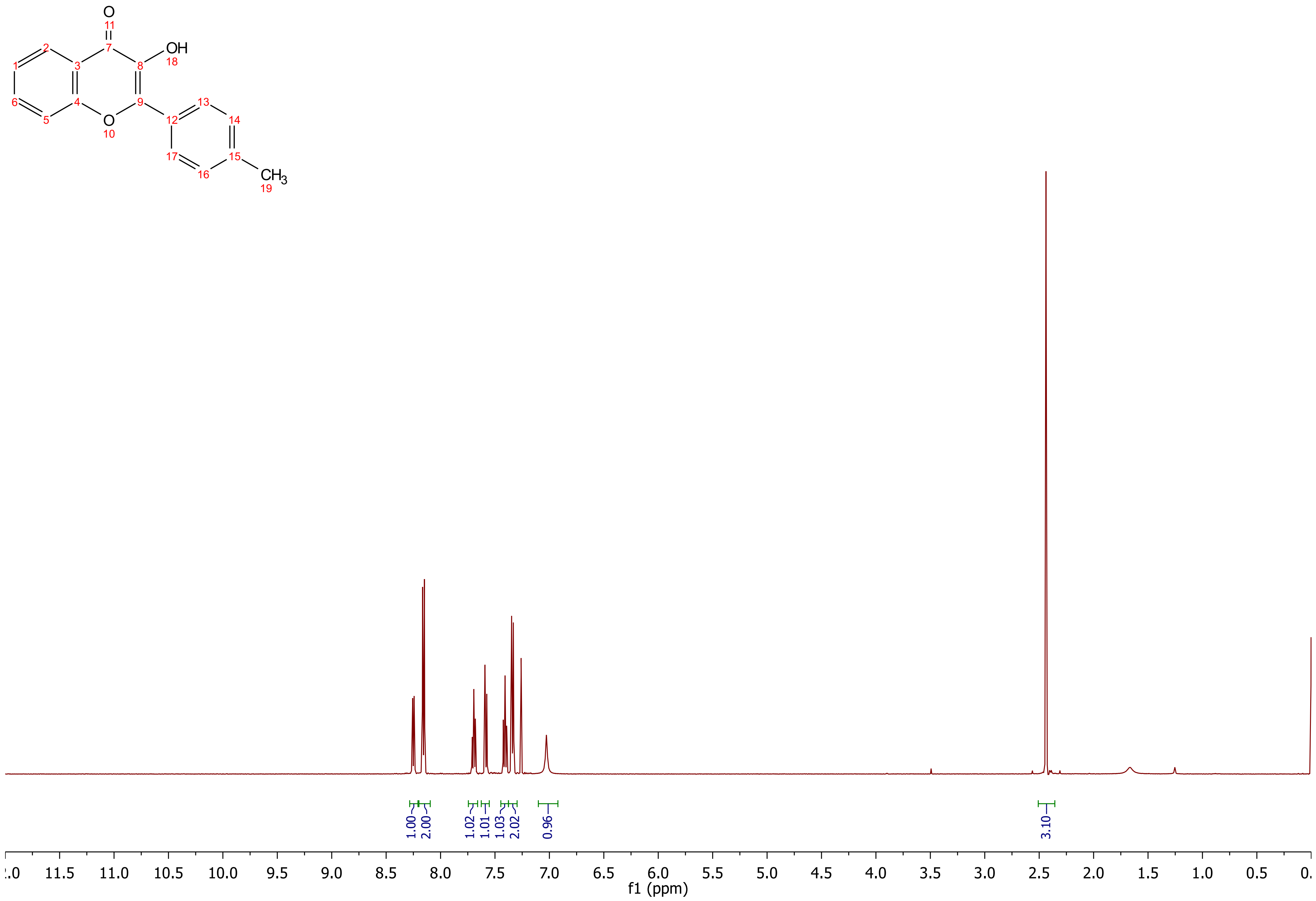
O
OH
O
CH3
1.00
2.00
1.02
1.01
1.03
2.02
0.96
3.10
11.5
11.0
10.5
10.0
9.5
9.0
8.5
8.0
7.5
7.0
6.5
6.0
5.5
5.0
4.5
4.0
3.5
3.0
2.5
2.0
1.5
1.0
0.5
f1 (ppm)

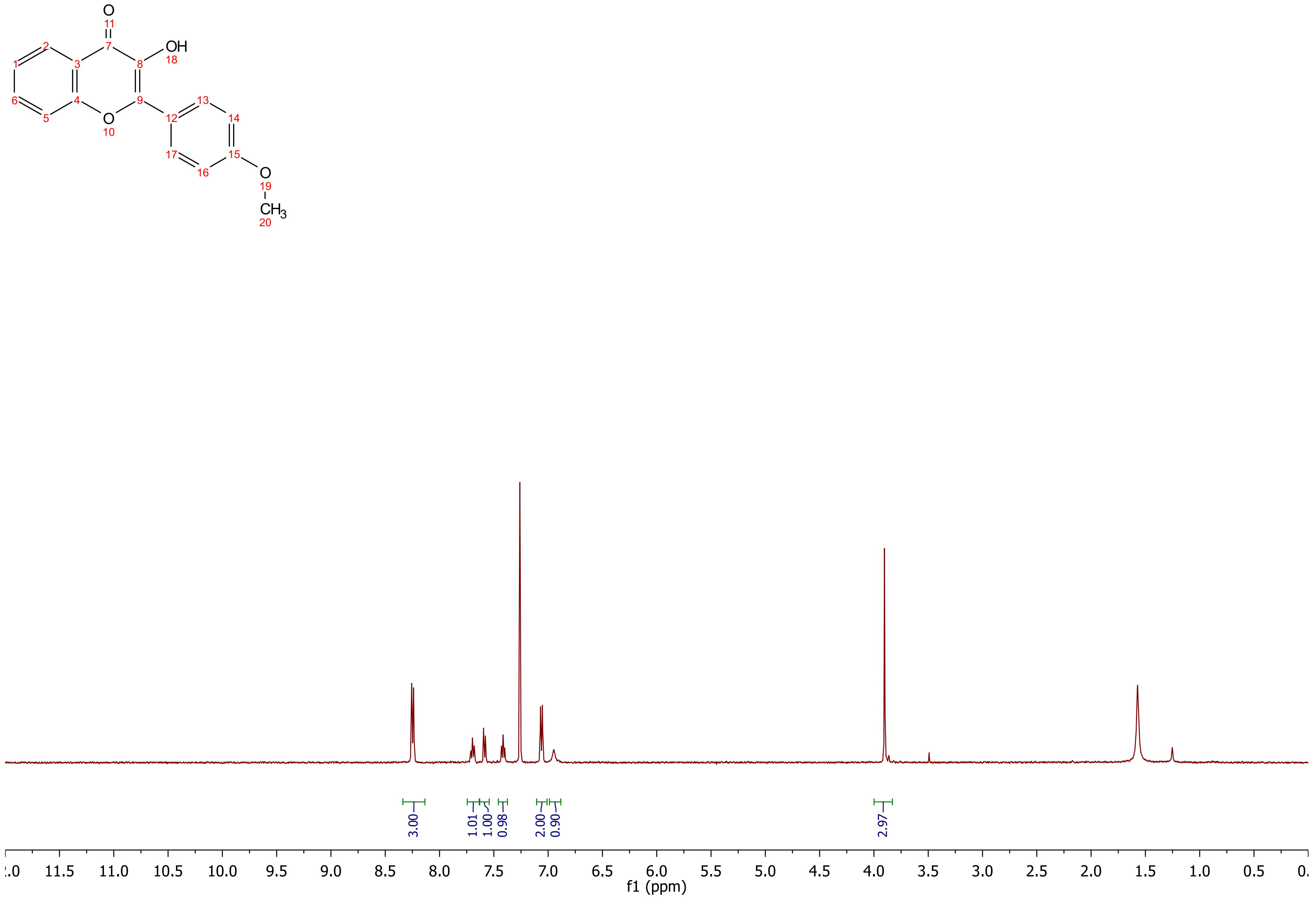

O
11
7
OH
18
8
9
1
2
3
4
5
6
O
10
12
13
14
15
16
17
O
19
CH3
20
3.00
1.01
1.00
0.98
2.00
0.90
2.97
11.5
11.0
10.5
10.0
9.5
9.0
8.5
8.0
7.5
7.0
6.5
6.0
5.5
5.0
4.5
4.0
3.5
3.0
2.5
2.0
1.5
1.0
0.5
f1 (ppm)

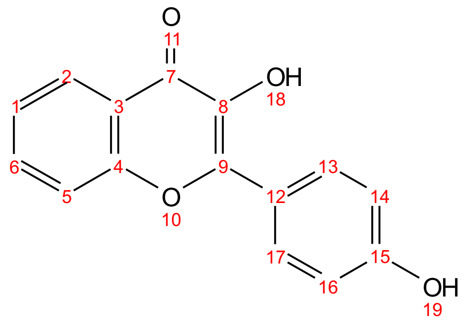

O
11
7
OH
18
8
9
1
2
3
4
5
6
O
10
12
13
14
15
16
17
OH
19

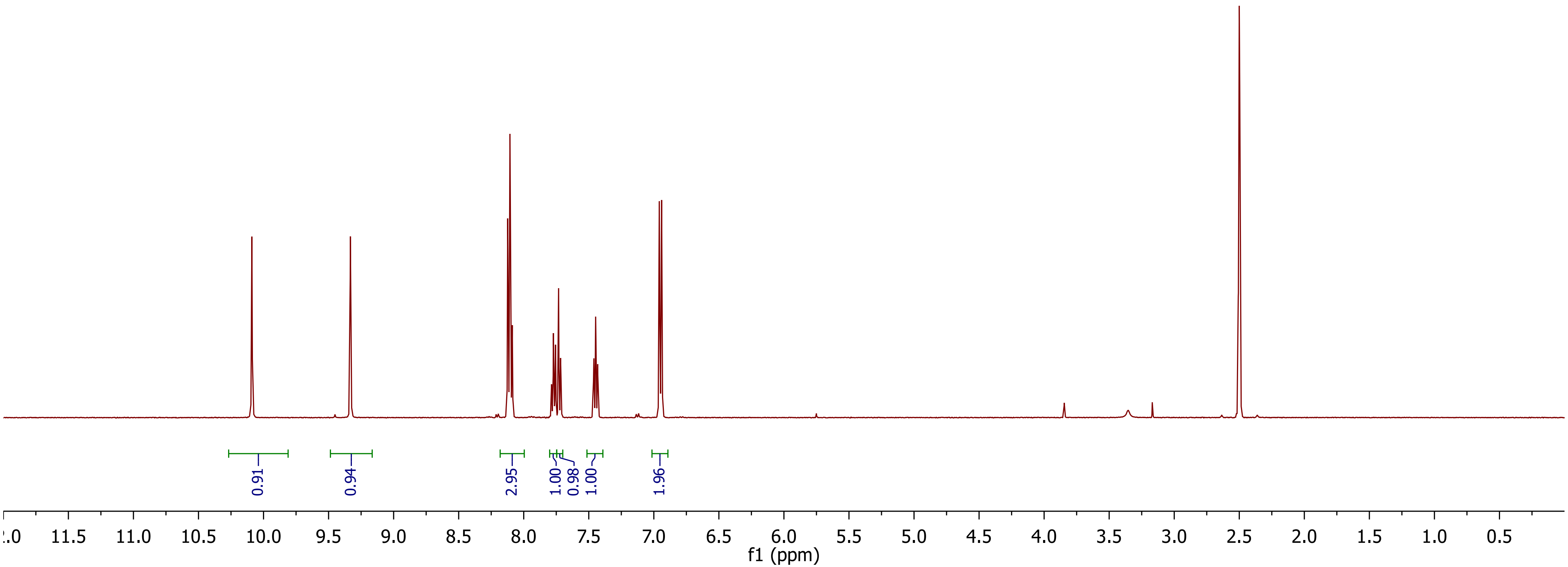

0.91
0.94
2.95
1.00
0.98
1.00
1.96
11.5
11.0
10.5
10.0
9.5
9.0
8.5
8.0
7.5
7.0
6.5
6.0
5.5
5.0
4.5
4.0
3.5
3.0
2.5
2.0
1.5
1.0
0.5
f1 (ppm)

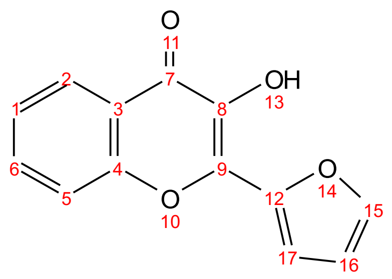
O
11
7
OH
13
8
9
O
14
15
16
17
12
O
10
1
2
3
4
5
6

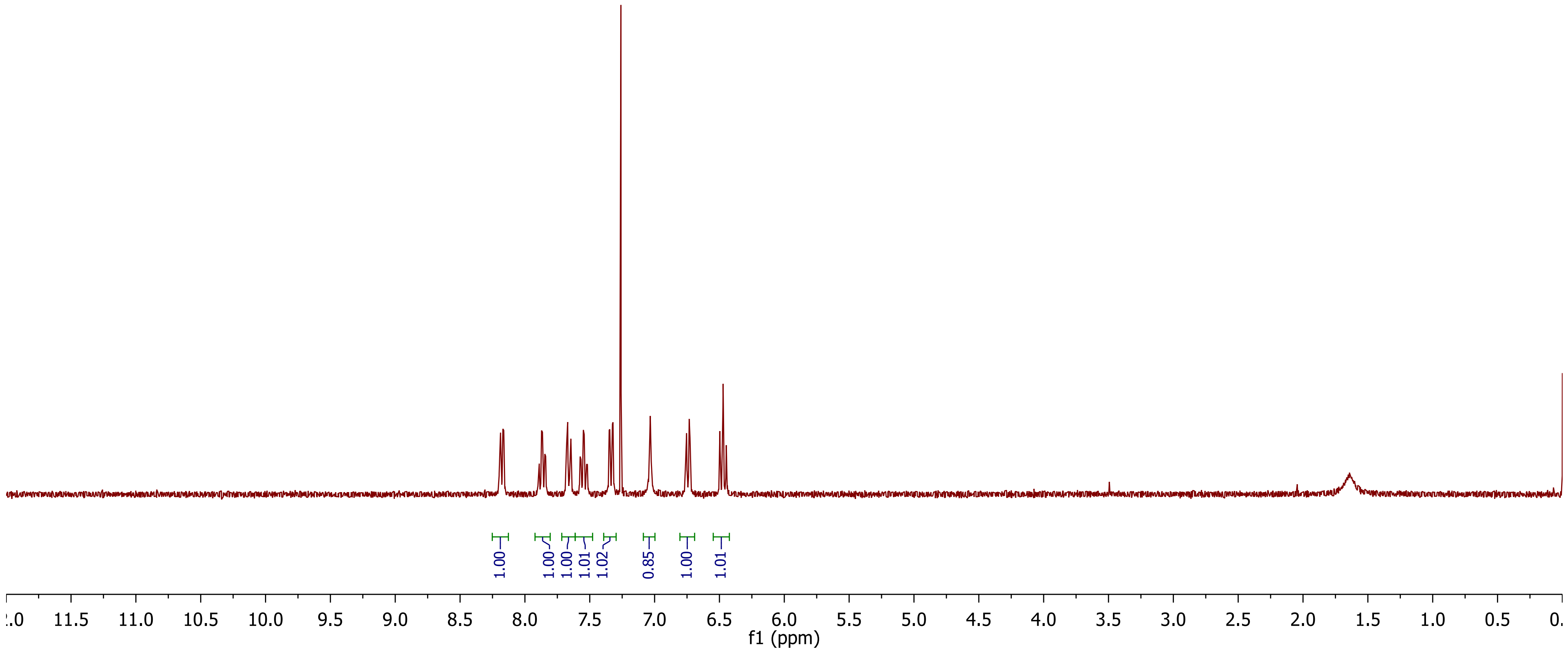
1.00
1.00
1.00
1.01
1.02
0.85
1.00
1.01
11.5
11.0
10.5
10.0
9.5
9.0
8.5
8.0
7.5
7.0
6.5
6.0
5.5
5.0
4.5
4.0
3.5
3.0
2.5
2.0
1.5
1.0
0.5
f1 (ppm)

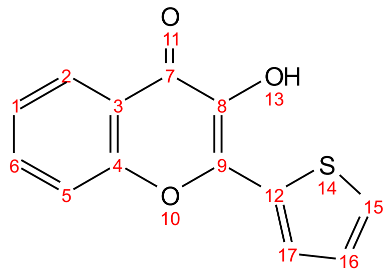
O
OH
S
O

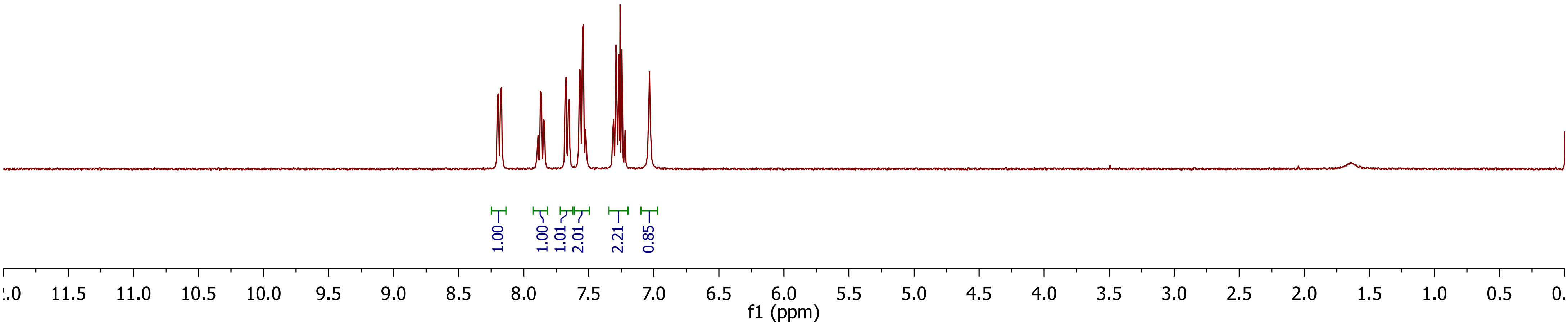
1.00
1.00
1.01
2.01
2.21
0.85
11.5
11.0
10.5
10.0
9.5
9.0
8.5
8.0
7.5
7.0
6.5
6.0
5.5
5.0
4.5
4.0
3.5
3.0
2.5
2.0
1.5
1.0
0.5
f1 (ppm)

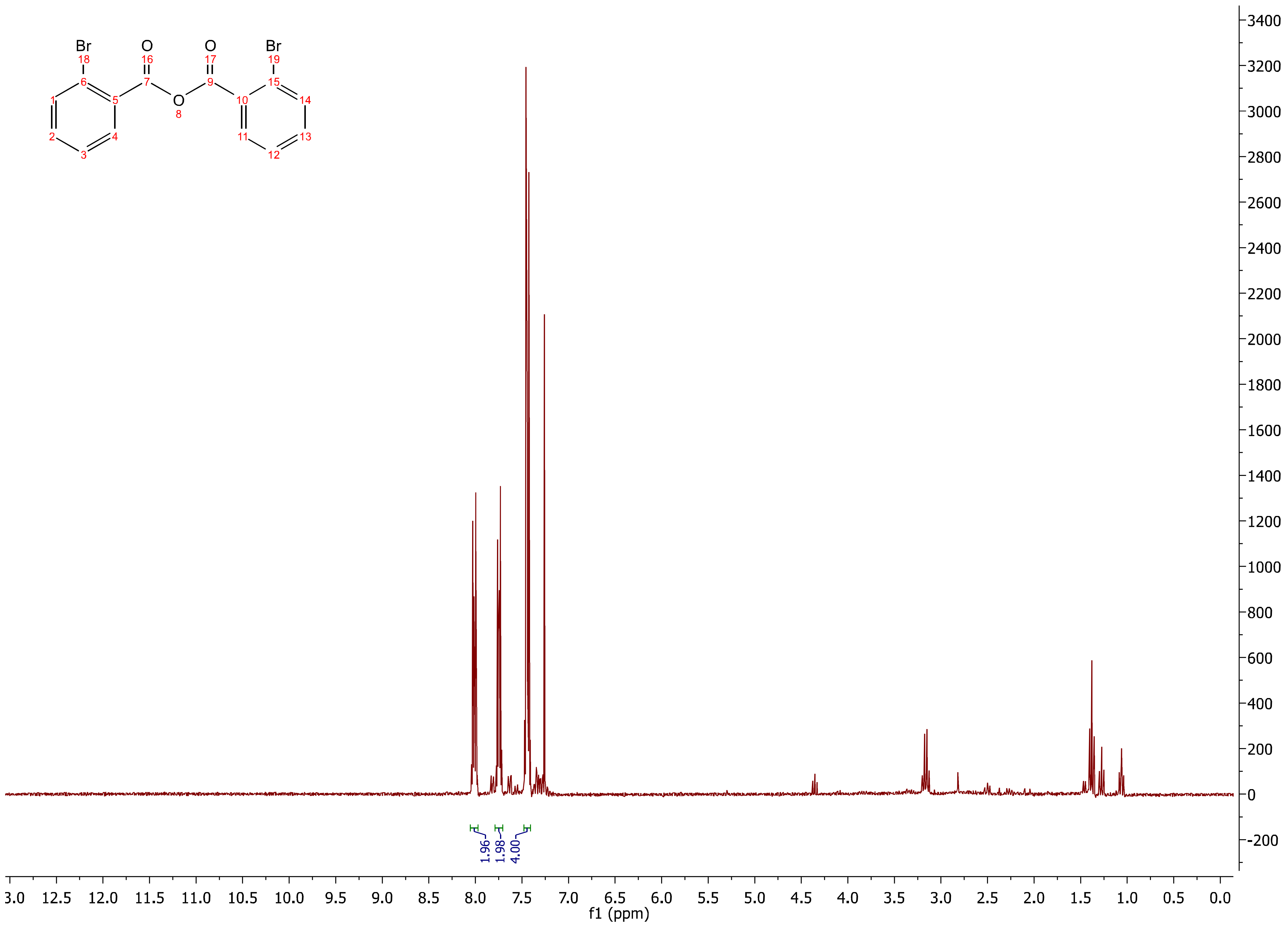

Br
O
O
Br
18
16
17
19
1
2
3
4
5
6
7
8
9
10
11
12
13
14
15
1.96
1.98
4.00
f1 (ppm)

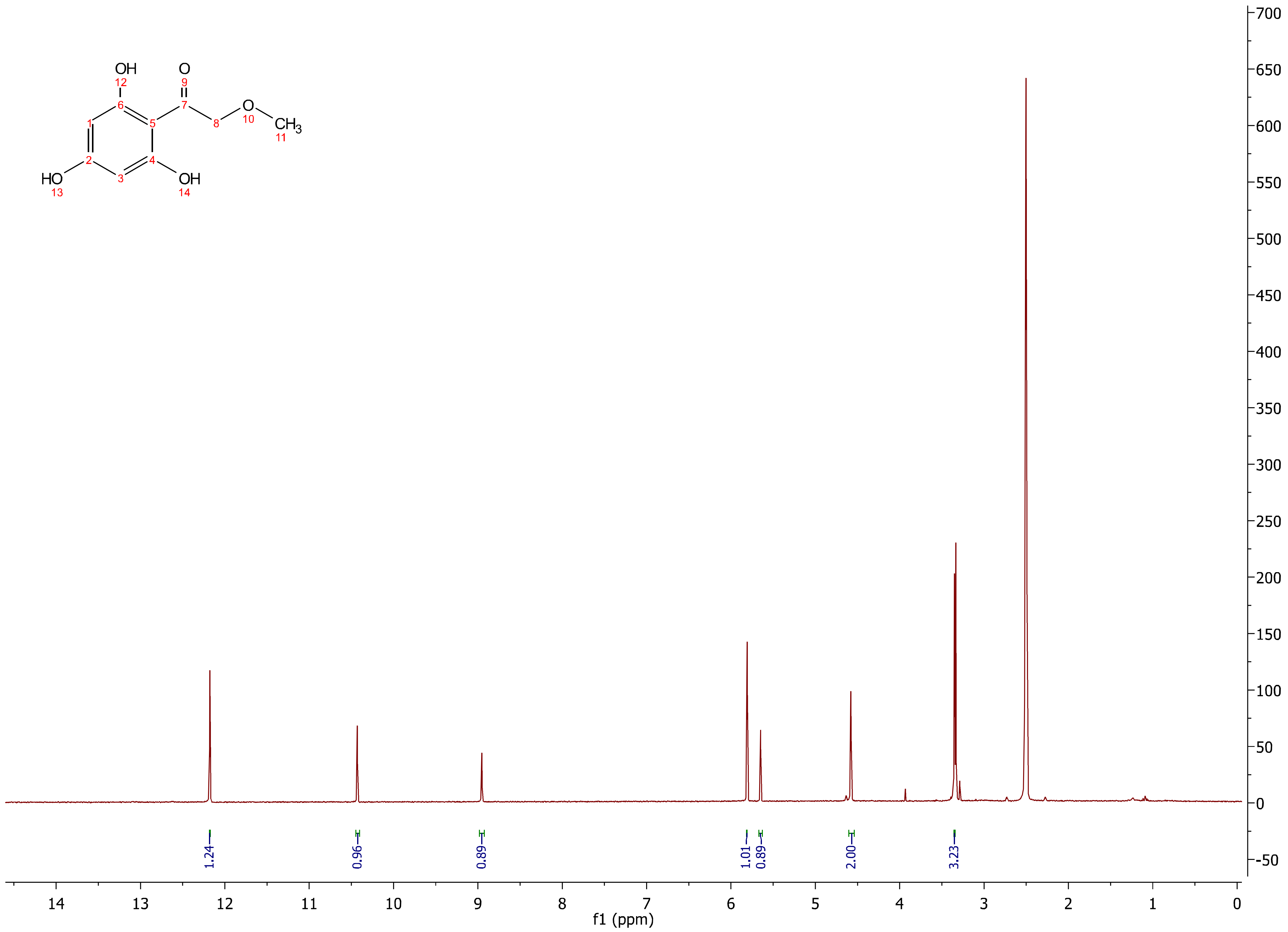

OH
O
HO
OH
CH3
1.24
0.96
0.89
1.01
0.89
2.00
3.23
f1 (ppm)

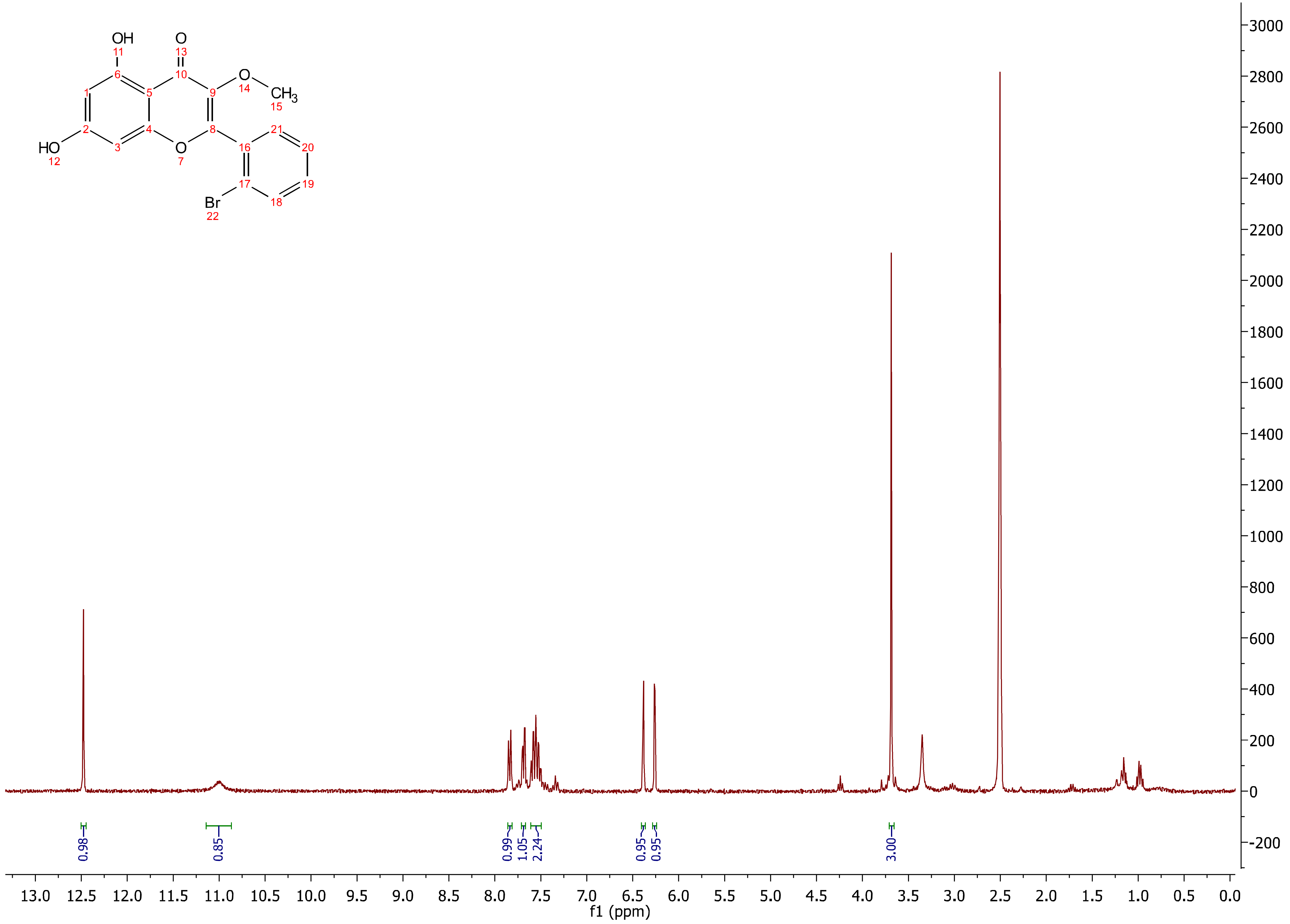

OH
O
O
CH3
HO
O
Br
0.98
0.85
0.99
1.05
2.24
0.95
0.95
3.00
f1 (ppm)

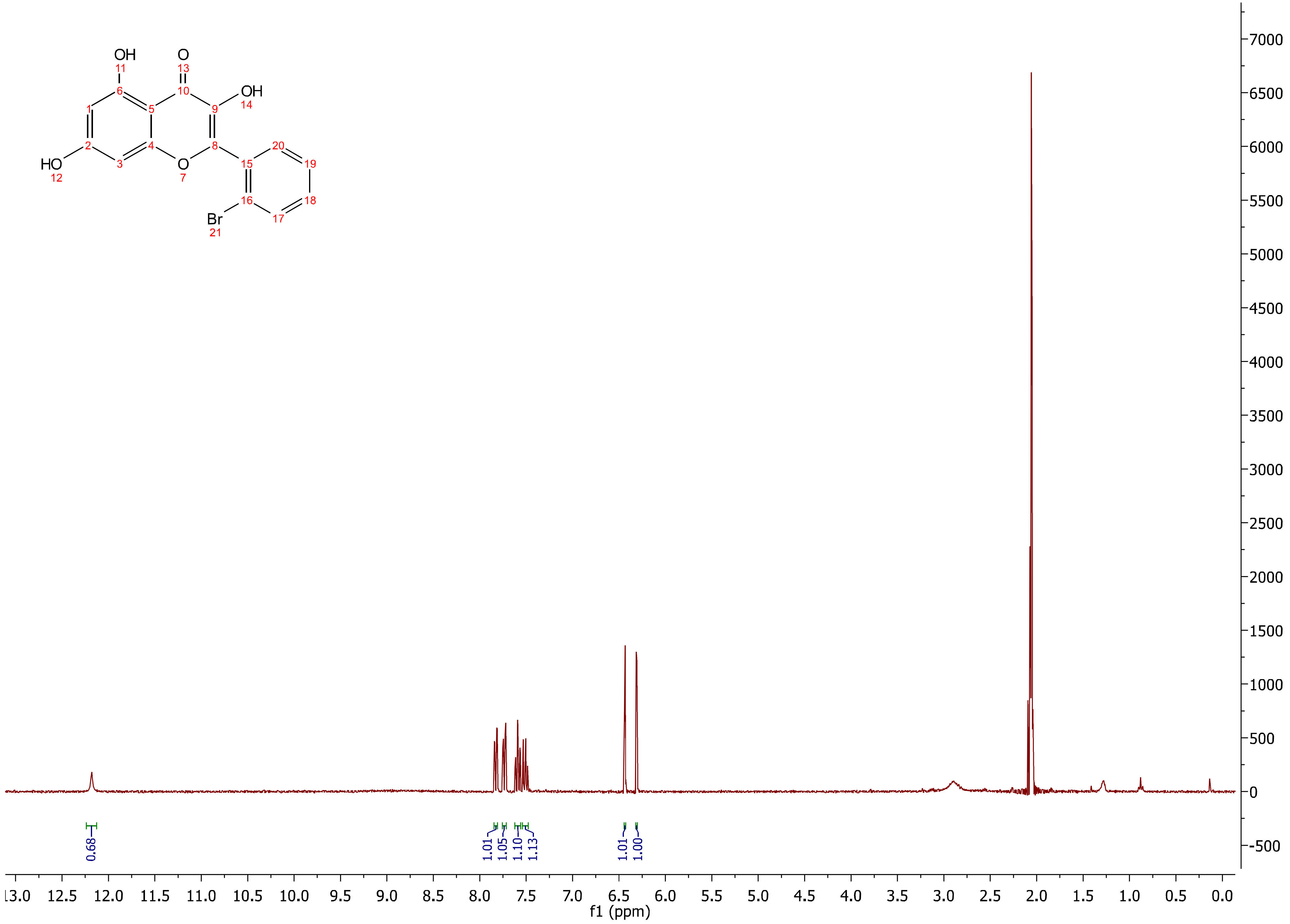

OH
O
OH
HO
O
Br
0.68
1.01
1.05
1.10
1.13
1.01
1.00
f1 (ppm)
